# Genome-wide characterization of FK506-binding proteins, parvulins and phospho-tyrosyl phosphatase activators in wheat and their regulation by heat stress

**DOI:** 10.3389/fpls.2022.1053524

**Published:** 2022-12-15

**Authors:** Anantika Suri, Harpreet Singh, Kirandeep Kaur, Anish Kaachra, Prabhjeet Singh

**Affiliations:** ^1^ Department of Biotechnology, Guru Nanak Dev University, Amritsar, India; ^2^ Department of Bioinformatics, Hans Raj Mahila Maha Vidyalaya, Jalandhar, India; ^3^ Biotechnology Division, Institute of Himalayan Bioresource Technology, Council of Scientific and Industrial Research, Palampur, HP, India

**Keywords:** FK506-binding proteins, parvulins, phospho-tyrosyl phosphatase activators, heat stress, wheat

## Abstract

Peptidyl-prolyl *cis-trans* isomerases (PPIases) are ubiquitous proteins which are essential for *cis-trans* isomerisation of peptide bonds preceding the proline residue. PPIases are categorized into four sub-families *viz*., cyclophilins, FK506-binding proteins (FKBPs), parvulins and protein phosphatase 2A phosphatase activators (PTPAs). Apart from catalysing the *cis-trans* isomerization, these proteins have also been implicated in diverse cellular functions. Though PPIases have been identified in several important crop plants, information on these proteins, except cyclophilins, is scanty in wheat. In order to understand the role of these genes in wheat, we carried out genome-wide identification using computational approaches. The present study resulted in identification of 71 FKBP (*TaFKBP*) 12 parvulin (*TaPar*) and 3 PTPA (*TaPTPA*) genes in hexaploid wheat genome, which are distributed on different chromosomes with uneven gene densities. The TaFKBP and TaPar proteins, besides PPIase domain, also contain additional domains, indicating functional diversification. *In silico* prediction also revealed that TaFKBPs are localized to ER, nucleus, chloroplast and cytoplasm, while the TaPars are confined to cytoplasm and nucleus. The TaPTPAs, on the contrary, appear to be present only in the cytoplasm. Evolutionary studies predicted that most of the *TaFKBP, TaPar* and *TaPTPA* genes in hexaploid wheat have been derived from their progenitor species, with some events of loss or gain. Syntenic analysis revealed the presence of many collinear blocks of *TaFKBP* genes in wheat and its sub-genome donors. qRT-PCR analysis demonstrated that expression of *TaFKBP* and *TaPar* genes is regulated differentially by heat stress, suggesting their likely involvement in thermotolerance. The findings of this study will provide basis for further functional characterization of these genes and their likely applications in crop improvement.

## Introduction

The plants cope up with different biotic and abiotic stresses through adjustments at physiological, biochemical and molecular levels, that include the production of antioxidative enzymes, accumulation of compatible solutes, signaling molecules, chaperones, etc. (Wang et al., 2003). The peptidyl-prolyl *cis-trans* isomerases (PPIases) are yet another class of proteins that have also been reported to play an important role in stress adaptation of plants (Sharma and Singh, 2003; Wang et al., 2003; Singh et al., 2019; Singh H et al., 2020; Thirumalaikumar et al., 2021). The PPIases are the only enzymes known that can catalyze the *cis-trans* transition of peptidyl-prolyl bonds. Though the peptide bonds in proteins occur in *trans* conformation, about 6.5% of the peptidyl-prolyl bonds occur in *cis* state due to the five-membered ring structure of prolyl residue (Galat and Rivière, 1998). The *cis-trans* transition of the peptidyl-prolyl bond is a rate-limiting step in correct folding of proteins and, hence, requires intervention of PPIases (Fischer et al., 1989) which are typical enzymes and follow the Michaelis-Menton kinetics. Depending upon their sensitivity towards immunosuppressive drugs, the PPIases are categorised into two sub-families, immunophilins and non-immunophilins. While the immunophilins comprise of cyclosporin A (CsA)-binding proteins, cyclophilins, and FK506-binding proteins, FKBPs, the non-immunophilin PPIases include structurally distinct parvulins and phospho-tyrosyl phosphatase activators (PTPAs), that are not sensitive to any of these immunosuppressive agents (Rahfeld et al., 1994). The cyclophilins and FKBPs, characterized by the presence of cyclophilin-like domain (CLD) and FK506-binding domain (FKBD), respectively, are ubiquitous proteins and have been reported in almost all organisms ranging from microbes to plants and animals (Göthel and Marahiel, 1999; Singh H et al., 2020; Yadav et al., 2022; Zhou et al., 2022). These proteins are localized in different sub-cellular organelles, *viz*., mitochondria, endoplasmic reticulum, cytosol and nucleus (Breiman et al., 1992; Luan et al., 1996; Xu et al., 1998; Carol et al., 2001). The cyclophilins and FKBPs are encoded by large gene families in plants. Genome-wide computational analysis revealed the presence of 21, 23, 24, 29, 30 and 38 different FKBPs in *Prunus persica*, *Arabidopsis thaliana*, *Solanum lycopersicum*, *Oryza sativa*, *Zea mays*, and *Malus domestica*, respectively (He et al., 2004; Gollan and Bhave, 2010; Wang W et al., 2012; Zhang Y et al., 2014; Dong et al., 2018; Waseem et al., 2018). Members of FKBPs have also been characterized in different fungal species such as *Aspergillus* spp. and *Penicillium* spp. (Joseph et al., 1999; Singh et al., 2021). Likewise, 28, 29, 30, 33, 38-78, 62, 83 and 91 cyclophilins have been reported in *O. sativa*, *A. thaliana*, *M. domestica*, *Medicago truncatula*, *Gossypium* spp., soybean, *Triticum aestivum* and *Brassica napus*, respectively (Gasser et al., 1990; Romano et al., 2004; Trivedi et al., 2012; Mainali et al., 2014; Hanhart et al., 2017; Chen et al., 2019; Singh et al., 2019; Ge et al., 2020; Wang et al., 2022). Contrary to the cyclophilins and FKBPs, the PTPAs and parvulins are less abundant and only one or two members have been reported in human, yeast and *Penicillium* spp. (Rahfeld et al., 1994; Lu et al., 1996; Jordens et al., 2006; Magnusdottir et al., 2006; Mueller et al., 2006; Singh et al., 2021).

In mammals, the cyclophilins and FKBPs are involved in immunosuppression, as interaction of these proteins with CsA and FK506, respectively, prevent transcription of genes encoding interleukin-2 (Liu et al., 1991; O'Keefe et al., 1992), leading to suppression of immune response. The immunophilins also regulate important cellular processes in plants, with the roles of cyclophilins discussed extensively in a recent review (Singh H et al., 2020 and references therein). Recently, an *Arabidopsis* cyclophilin, AtCYP18-1, was reported to play an important role in splicing of introns that are retained in response to heat stress during germination (Jo et al., 2022). Cyclophilin *ROC3* was implicated in regulation of ABA-induced stomatal closure and drought stress response in *Arabidopsis* (Liu et al., 2021). Similarly, ectopic expression of a pigeonpea cyclophilin (*CcCYP*) was shown to confer tolerance against multiple abiotic stresses in transgenic rice (Juturu et al., 2021). The FKBPs too have been shown to perform diverse cellular functions *viz*., signal transduction (Gopalan et al., 2004), transcription (Kang et al., 2008), assembly of multiprotein complexes (Geisler and Bailly, 2007; Nigam et al., 2008; Boudko et al., 2014). protein trafficking (Luan et al., 1994), apoptosis (Meiri et al., 2010), fertility (Yu et al., 2012), biosynthesis of long chain fatty acids (Harrar et al., 2003), regulation of gene expression (Li and Luan, 2010), photosynthetic membrane assembly in plants (Lima et al., 2006) and as histone chaperones (Singh A et al., 2020). Several FKBPs also exhibit chaperonic activity that is independent of PPIase activity. For example, one of the wheat FKBPs, FKBP73, exihbited chaperonic function despite abrogation of its PPIase activity (Kurek et al., 2002). Expression of *FKBP* genes, *FKBP62* and *FKBP65*, was enhanced in response to wounding, NaCl and malondialdehyde treatment in *Arabidopsis* (Vucich and Gasser, 1996; Weber et al., 2004; Aviezer-Hagai et al., 2007; Meiri and Breiman, 2009). Yu et al. (2017) demonstrated that constitutive expression of a *Z. mays FKBP* gene, *ZmFKBP20-1*, resulted in higher tolerance to drought and salt stress in the transgenic *Arabidopsis* plants. The role of a rice FKBP, OsFKBP20-1b, in stress adaptation was attributed to regulation of RNA processing through its interaction with a splicing factor OsSR45 (Park et al., 2020).

It is, thus, evident that different PPIases play important roles in growth and development processes of plants as well as in adaptation to different abiotic stresses. Earlier studies by our group demonstrated that the cyclophilin family in wheat consists of 83 genes, several of which are modulated under heat stress (Singh et al., 2019). However, except for cyclophilins (Singh et al., 2019), information on PPIases belonging to FKBPs, parvulins and PTPAs is scanty in this important crop plant. Therefore, the present study was undertaken to carry out *in silico* identification and characterization of FKBPs, parvulins and PTPAs in wheat and to study their regulation by thermal stress. The results of this study revealed that the wheat genome encodes for 71 FKBPs, 12 parvulins, and 3 PTPAs, and expression of several of these genes is modulated differentially by heat stress. These findings will provide impetus for further functional characterization of these gene families, and their likely applications in crop improvement through biotechnological and breeding strategies.

## Materials and methods

### Identification and retrieval of *FKBP*, *parvulin* and *PTPA* gene sequences

The different FKBPs, parvulins, and PTPAs encoded by the wheat genome were identified by using the coding (CDS) and protein sequences of different organisms for homology-based search. For FKBPs, sequences from *O. sativa*, *A. thaliana*, *P. persica* and *M. domestica*; for PTPAs, sequences from *Drosophila melanogaster*, *Homo sapiens*, *Saccharomyces cerevisiae* and *Saccharomyces pombe*; and for parvulins, sequences of *A. thaliana*, *H. sapiens*, *S. cerevisiae* and *Escherichia coli* were employed for this study. The CDS and protein sequences for different organisms were obtained from their respective databases (UniProt; Universal Protein knowledgebase; https://www.uniprot.org/, NCBI; National Centre for Biotechnology Information; https://www.ncbi.nlm.nih.gov/, RGAP; Rice Genome Annotation Project; http://rice.plantbiology.msu.edu/analyses_search_locus.shtml, TAIR; The Arabidopsis Information Resource; https://www.arabidopsis.org/, GDR; Genome Database for Rosaceae; https://www.rosaceae.org/retrieve/sequences) and from other publicly available sources (**Supplementary Table S1**). BLASTN and BLASTP (Ensembl Plants; https://plants.ensembl.org) programs were used for homology search with an e-value cut off of 10 and 1, respectively. The redundant sequences having the same transcript IDs were removed from the dataset. The matches based on score, coverage and percent identity were retrieved and selected for further analysis. For FKBPs, the retrieved protein sequences were used to build a Hidden Markov model (HMM; HMMER software package; Eddy, 2011). This model was used as a query for running HMM search to obtain more distantly homologous FKBPs from the wheat proteome (IWGSC RefSeq v2.0). Ensembl plants (https://plants.ensembl.org/Triticum_aestivum/Info/Index) was used to download the genomic sequences, CDS, untranslated regions (UTRs), introns, exons, cDNA as well as protein sequences of the corresponding matches.

### Conserved domain search and subcellular localization prediction

Domain analysis was performed using PFAM (http://pfam.xfam.org; Finn et al., 2016), Prosite (https://prosite.expasy.org/; Sigrist et al., 2012) and conserved domain database of NCBI (http://www.ncbi.nlm.nih.gov/Structure/cdd/wrpsb.cgi). Only those members which displayed the presence of typical functional domain of their respective PPIase family were subjected to additional characterization and investigation. Graphical representation of the domains and motifs was performed using IBS1.0 stand alone program (Liu W et al., 2015). The subcellular localization was predicted by using LocTree3 protein subcellular localization prediction system (https://rostlab.org/services/loctree3/; Goldberg et al., 2014).

### Sequence analysis

The deduced amino acid sequences were aligned with Clustal Omega (https://www.ebi.ac.uk/Tools/msa/clustalo/). The molecular weights and pIs of the putative PPIases were predicted by biosynthesis’s peptide property calculator web server Version 3.1 (https://www.biosyn.com/peptidepropertycalculator/peptidepropertycalculator.aspx). Amino acid sequences of the respective domains were extracted using EMBOSS: extractseq web interface (bioinformatics.nl/cgi-bin/emboss/extractseq; Olson, 2002). Active site residues of the aligned domain sequences were analysed by employing ESPript 3.0 (http://espript.ibcp.fr/ESPript/cgi-bin/ESPript.cgi; Robert and Gouet, 2014).

### Gene structure, motif analysis and phylogenetic analysis of FKBPs, parvulins, and PTPAs

Gene structures were analyzed using GSDS 2.0 server (http://gsds.cbi.pku.edu.cn/; Hu et al., 2015). Conserved motifs of the protein sequences were identified with MEME Suite (https://meme-suite.org/meme/; Bailey et al., 2015), and edited using TBtools (https://github.com/CJ-Chen/TBtools; Chen et al., 2020). Mega X was employed to construct phylogenetic trees by Neighbor Joining (NJ) method using 1000 bootstrap values. Respective protein sequences of FKBPs, parvulins and PTPAs from *T. aestivum*, *A. thaliana*, *O. sativa*, *Aegilops tauschii*, *Triticum dicoccoides* and *Triticum urartu* were used for the construction of phylogenetic trees. Constructed phylogenetic trees were subsequently edited using Interactive Tree of Life web server (https://itol.embl.de/upload.cgi).

### Chromosome mapping and syntenic analysis

Predicted wheat *FKBP*, *parvulin* and *PTPA* genes were mapped to the corresponding chromosomes using MapChart2.32 (Voorrips, 2002). The map obtained was further processed with InKscape (https://inkscape.org/) to highlight various features. Gene duplication events between *T. aestivum*, its progenitors (*T. urartu*, *T. dicoccoides* and *A. tauschii*), *O. sativa* and *A. thaliana* were analyzed with MCScanX (Multiple Collinearity Scan toolkit, https://github.com/wyp1125/MCScanX; Wang Y et al., 2012) keeping threshold values of <1e-4 and 5 for e-value and match size, respectively. The tandem gene duplications and collinear blocks identified with MCScanX were plotted using shinyCircos online application (https://venyao.shinyapps.io/shinyCircos/; Yu et al., 2017). Dual synteny plot was generated by TBtools software (https://github.com/CJ-Chen/TBtools; Chen et al., 2020).

### Phylogenetic analysis

Phylogenetic tree was generated to understand the evolutionary relationship among different FKBP, parvulin and PTPA proteins. Alignment of the amino acid sequences of all the proteins was carried out by Clustal Omega (http://www.ebi.ac.uk/Tools/msa/clustalo/; Sievers et al., 2011). The phylogenetic tree was generated with MEGA X (Molecular Evolutionary Genetics Analysis) across computing platforms (Kumar et al., 2018) using Neighbour-Joining method with 1000 bootstrap replicates. The tree was exported to the Interactive Tree of Life (https://itol.embl.de/; Letunic and Bork, 2016) for subsequent processing and annotation.

### Identification of cis-regulatory elements

The upstream 2 kb promoter regions of the gene sequences were retrieved from Ensembl plants database and used as inputs to search cis-regulatory elements in the database (HSEAT, Heat Shock Element Analysis Tool; https://sourceforge.net/projects/heast/).

### Effect of heat stress on expression of *FKBP*, *parvulin* and *PTPA* genes

Seeds of wheat (*T. aestivum* L.) cultivar HD2967 were surface sterilized using Tween 20 and subsequently sown in soil, and grown in the culture room at 25 ± 2 °C. For acclimation to heat stress, the seven days old seedlings were exposed to 37°C for 2 h. Heat stress was imposed by incubating the seedlings at lethal temperature of 50°C for 4.5 h with and without acclimation at 37°C for 2 h. Seedlings kept at 25 °C served as experimental control. Healthy seedlings after the treatments were collected at different time intervals and snap frozen in liquid nitrogen until further analysis.

Total RNA from the leaves was isolated using TRIZOL solution (Invitrogen, USA), and reverse transcription was performed with random hexamer primers using superscript III First-strand synthesis system kit following the manufacturer’s instructions (Invitrogen, USA). For expression analysis, primers binding to common regions of different homoeologs were designed with Primer-BLAST tool (https://www.ncbi.nlm.nih.gov/tools/primer-blast/index.cgi?LINK_LOC=BlastHome). The quantitative real-time PCR (qRT-PCR) was performed on Ariamx real-time PCR system (Agilent Technologies, USA) with Brillant III ultra-Fast SYBR green QPCR master mix (Agilent Technologies, USA). Expression analysis was carried out in three independent biological replicates. *Actin* was used as an internal control for normalization and the fold change was determined using 2^−ΔΔCt^ method (Livak and Schmittgen, 2001). The data obtained were subjected to one-way analysis of variance (ANOVA; graphpad prism).

For digital expression analysis (DGE), expression pattern of *TaFKBP* and *TaPar* genes was determined using WheatExp containing homoelogue-dependent gene expression profiles for wheat (https://wheat.pw.usda.gov/WheatExp/; Pearce et al., 2015). Protein sequences were searched against WheatExp database using tblastn and the matched hits were retrieved. RNAseq expression data from a previous work (Liu Z et al., 2015) was used as a reference for DGE analysis of the selected PPIases. The heat map of normalized (log10) expression values was generated using heatmap function from the NMF CRAN library (https://cran.r-project.org/web/packages/NMF/index.html) in RStudio version 4.0.2 (http://www.rstudio.com/; Myers et al., 2020). Hierarchical clustering of rows was performed with Euclidean distance and complete linkage.

### Protein-protein interaction network

The protein sequences of TaFKBPs, TaPars and TaPTPAs were submitted to STRING (v11.5) to predict the relationships between themselves and with other proteins (Szklarczyk et al., 2019) based on orthologs from *A. thaliana*. The parameter “max number of interactors to show” was set to “no more than 10 interactors” while the interacting partners were subjected to k-means clustering to obtain 3 clusters. The query protein (*T. aestivum*) names were used to label the nodes.

## Results

### Identification of gene families encoding wheat FKBPs, Parvulins and PTPAs

**Figure 1:**
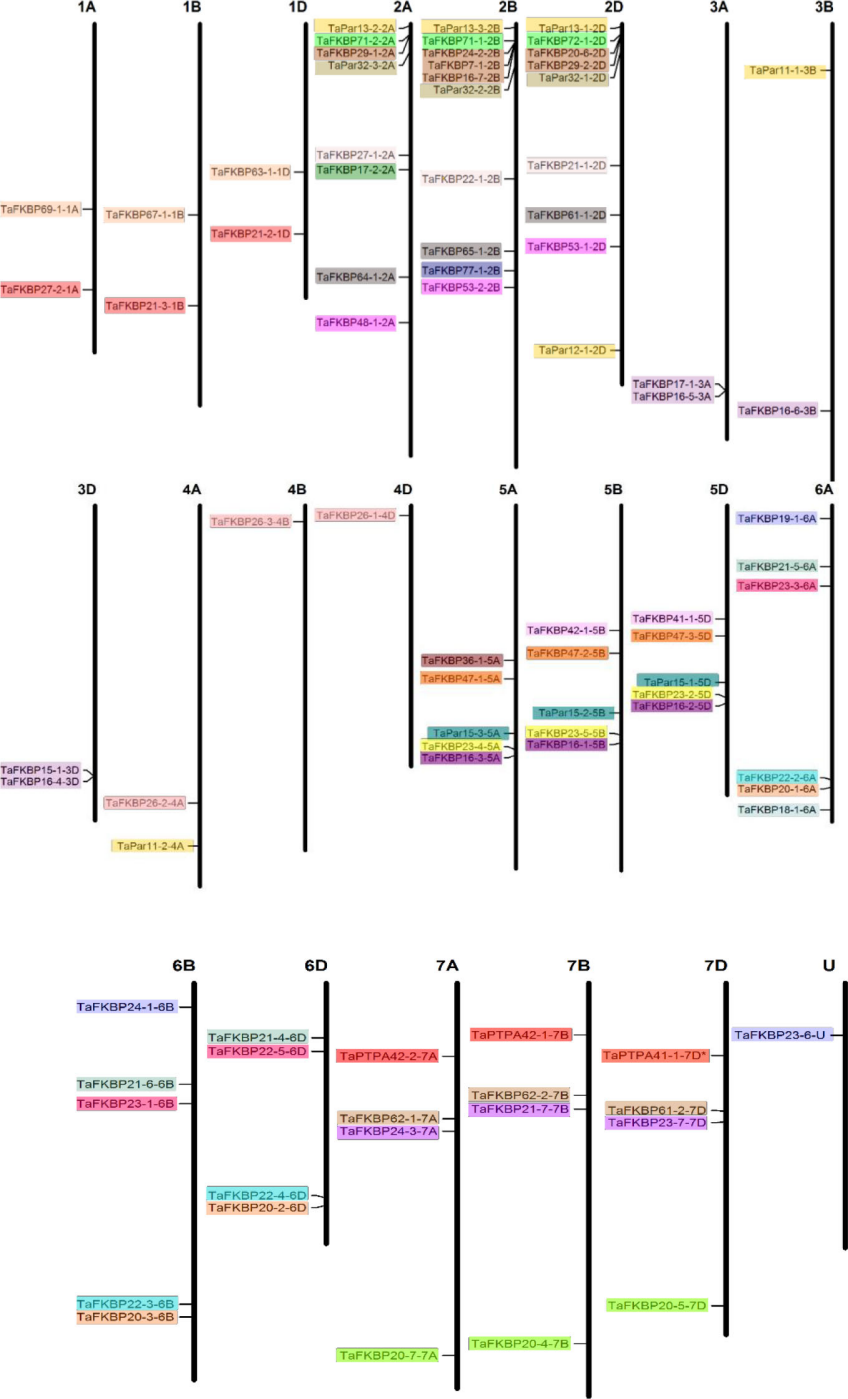
Chromosomal distribution of the genes encoding FKBPs, parvulins and PTPAs in wheat. The chromosome size was determined from the IWGSC1.0 assembly and is drawn to scale. Homeologous groups of genes are represented in same colors (U represents uncharacterized part of genome).

Bioinformatics analysis of wheat genome using Ensembl plants resulted in identification of 71 *TaFKBP*, 12 *TaPar* and 3 *TaPTPA* genes (**Supplementary Tables S1**, **S2**). The nomenclature adopted in this study is as used earlier for wheat cyclophilins (Singh et al., 2019). The wheat FKBP (TaFKBP), parvulin (TaPar) and PTPA (TaPTPA) proteins were suffixed with their predicted molecular weight and chromosomal localization. Localization analysis revealed that the *TaFKBP* genes are present on all the 21 wheat chromosomes, with the maximum number (8) on chromosome 2B and the lowest, one each, on chromosomes 3B, 4A, 4B and 4D (**Figure 1**). In contrast, only eight wheat chromosomes showed the presence of *TaPar* genes, with the chromosome 2D harbouring the maximum three members. The chromosomes 3B, 4A, 5A, 5B and 5D were predicted to contain only a single *TaPar* gene (**Figure 1**). Only a single homeologue triplet of *PTPA* genes was observed in wheat, which were localized on chromosome 7A, 7B and 7D, respectively. *In silico* analysis of one of the *FKBPs, TaFKBP23-6-U*, indicated the localization of this gene in an uncharacterized part of wheat genome, denoted by ‘*U*’ (**Figure 1**). Though majority of the identified genes were present as triplets due to the three progenitor genomes AA, BB and DD, contributed by the diploid species *T. urartu, Aegilops speltoides* and *A. tauschii*, respectively (Feldman and Levy, 2005), absence or existence of additional copies was also observed for some *TaFKBP* and *TaPar* genes. For example, *TaFKBP29-1-2A, TaFKBP24-2-2B and TaFKBP29-2-2D*, a homeologue triplet localized on chromosome 2A, 2B and 2D, depicted the presence of two additional copies on chromosome 2B (*TaFKBP7-1-2B; TaFKBP16-7-2B*) and one on 2D (*TaFKBP20-6-2D*). The gene encoding TaFKBP7-1-2B appears to be a paralogue containing partial FKBD sequence (missing N-terminal region) having more than 97℅ identities with TaFKBP29-1-2A, TaFKBP24-2-2B and TaFKBP29-2-2D proteins. Similarly, the *TaPar* genes, *TaPar13-2-2A, TaPar13-3-2B* and *TaPar13-1-2D* also exhibited additional copies on chromosomes 2D (*TaPar12-1-2D*), 3B (*TaPar11-1-3B*) and 4A (*TaPar11-2-4A*).

The number of amino acid residues in TaFKBPs and TaPars varies from 68 to 649, and 99 to 301, respectively, whereas the molecular weights of these sub-families range between 7.59 to 77.10 kDa, and 11.06 to 32.70 kDa, respectively (**Supplementary Table S1**). Genome-wide analysis revealed that only three PTPAs are encoded by the wheat genome, with TaPTPA41-1-7D* and TaPTPA42-1-7B consisting of 393 amino acid residues each, compared to 394 in TaPTPA42-2-7A. Since the CDS and amino acid sequences of TaPTPA41-1-7D* contain some unannotated residues, its exact molecular weight and pI could not be predicted. The molecular weights of TaPTPA42-1-7B and TaPTPA42-2-7A were computed as 42.46 and 42.57 kDa, respectively. Isoelectric points (pIs) of different TaFKBPs, TaPars and TaPTPAs range between 4.32 to 10.68, 6.15 to 9.92, and 6.9 to 7.1, respectively (**Supplementary Table S1**). While the TaFKBPs were predicted to localize to different subcellular compartments *viz.*, ER (1), nucleus (6), chloroplast (32) and cytoplasm (32), the TaPars are likely to localize only in cytoplasm (4) and nucleus (8). On the contrary, all the three TaPTPA proteins were predicted to be present only in the cytoplasm.

### Chromosome localization and synteny analysis of wheat *TaFKBP*, *TaPar* and *TaPTPA* genes

**Figure 2:**
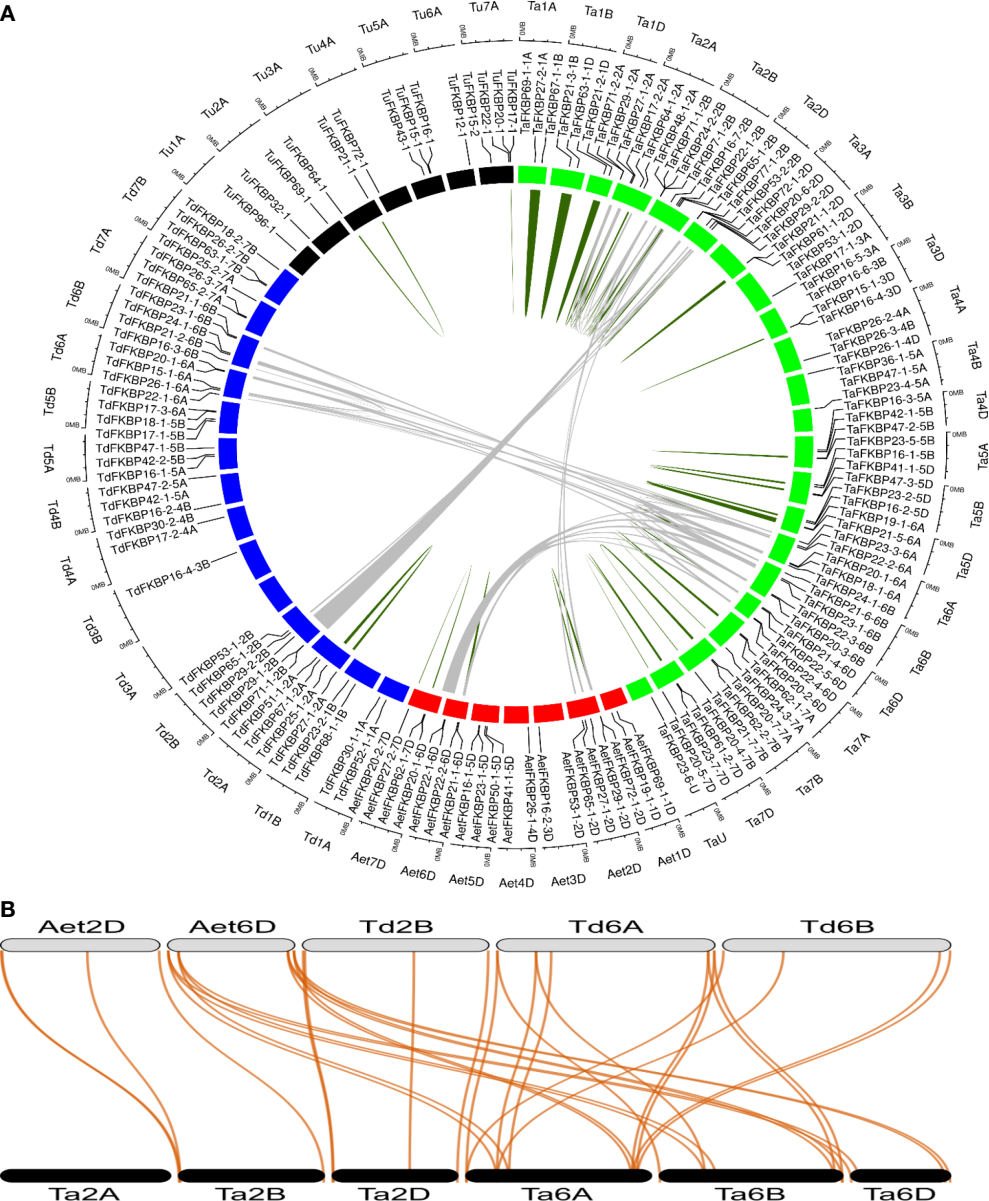
Collinearity analysis of *TaFKBP* genes using genome sequences of *T. aestivum* (Ta, AABBDD, green box) and progenitor species *A*. *tauschii* (AeT, DD, red box), *T. urartu* (Tu, AA, black box) and *T. dicoccoides* (Td, AABB, blue box). **(A)** A circular layout of *TaFKBP* orthologues with collinear blocks joined across the corresponding chromosomes in *T. aestivum* and progenitor species by grey lines. The tandemly duplicated genes are marked with dark green lines. The scale on the circle is in Megabases. **(B)** The specific collinear blocks between *Ta* (black color), *Aet* (grey color) and *Td* (grey color) are shown as a dual synteny plot. The collinear genes are connected by red lines.

Gene duplication events are vital for the amplification of gene families and their subsequent evolution in wheat. A tandem duplication event occurs when two or more similar genes are found located within a 200 kb of chromosomal region (Holub, 2001). Duplication events that gave rise to *TaFKBP*, *TaPar* and *TaPTPA* genes were studied in *O. sativa*, *A. thaliana*, *T. aestivum* (AABBDD) and its progenitors, *T. urartu* (AA), *T. dicoccoides* (AABB) and *A. tauschii* (DD) using MCScanX (**Supplementary Table S3**) (Wang Y et al., 2012). Due to absence of genome sequence of the B sub-genome donor *A. speltoides*, wild emmer wheat (*T. dicoccoides*) was used as the source of this sub-genome. Neither *T. aestivum* nor any of its progenitors showed syntenic relationship for parvulin and the PTPA genes. Syntenic relationships were also not observed for FKBP genes between wheat, *O. sativa*, *A. thaliana* and *T. urartu*. Several tandem duplicated pairs of *FKBP* genes were identified in *T. urartu* (2)*, T. dicoccoides* (2)*, A. tauschii* (3) and *T. aestivum* (29) (**Figure 2A**; **Supplementary Table S4**). Microcollinearity analyses of the genome segments across different progenitor species revealed the presence of several orthologous segments sharing collinear blocks of *FKBP* genes (**Figures 2A, B**; **Supplementary Table S5**). *T. aestivum* contains four blocks of *FKBP* genes collinear with each of its two progenitor species, *A. tauschii* and *T. dicoccoides.* In addition, six segmentally duplicated blocks of *FKBP* genes were also identified in *T. aestivum*, compared to a single block in *T. dicoccoides*. These results indicate that tandem as well as segmental gene duplication events might have played an important role in the evolution of *FKBP* gene family in wheat. Furthermore, our results also suggest that the wheat *FKBP* genes are entirely derived from its progenitors since most of the wheat chromosomes, except 1B, 2A, 2B, 2D and 3D, harbor the same number of genes as are present on the corresponding chromosomes of the sub-genome donors (**Supplementary Table S6**). However, the chromosomes 1B, 2A, 2B, 2D and 3D depicted the presence of higher number of *FKBP* genes relative to the sub-genome donors, indicating enrichment due to duplication events. Another reason for this difference could be the loss of these genes in *T. dicoccoides* and *A. tauschii* during evolution. Relative to *T. dicoccoides* and *T. aestivum*, the difference in *FKBP* genes in *T. urartu* may also be ascribed to similar events. In contrast, the wheat chromosomes 1D, 4B and 5D harbor fewer number of *FKBP* genes than its sub-genome donors, suggesting loss during the course of evolution. Similar trend was also observed for *TaPar* genes on chromosomes 2A, 2B and 2D (**Supplementary Table S7**). The *TaFKBP* genes (71) showed homology with 14, 40, 20, 16, 23, 16 and 18 counterparts in *T. urartu, T. dicoccoides, A. tauschii*, *Arabidopsis*, *O. sativa*, *P. persica* and *M. domestica*, respectively. Similarly, different homologues of *TaPars* (12) were also observed in *A. thaliana* (3), *E. coli* (1), human (2), *S. cerevisiae* (1)*, T. urartu* (3)*, T. dicoccoides* (9) and *A. tauschii* (5). The *A. thaliana*, *O. sativa*, *T. urartu* and *A. tauschii* also depicted the presence of a single homologue of *TaPTPA* gene. Absence of *PTPA* genes in one of the wheat progenitors, *T. dicoccoides* suggests, that the gene encoding TaPTPA42-1-7B might have been derived from the wheat B sub-genome donor *A. speltoides.* Alternately, it is also likely that these genes might have been lost during evolution, as observed for *14-3-3* gene family in wheat (Shao et al., 2021). This study provides further evidence that the genes encoding FKBPs, parvulins and PTPAs are conserved across major plant species and their abundance in the wheat genome may be attributed to various duplication events during the course of evolution.

### Gene structure organization

**Figure 3:**
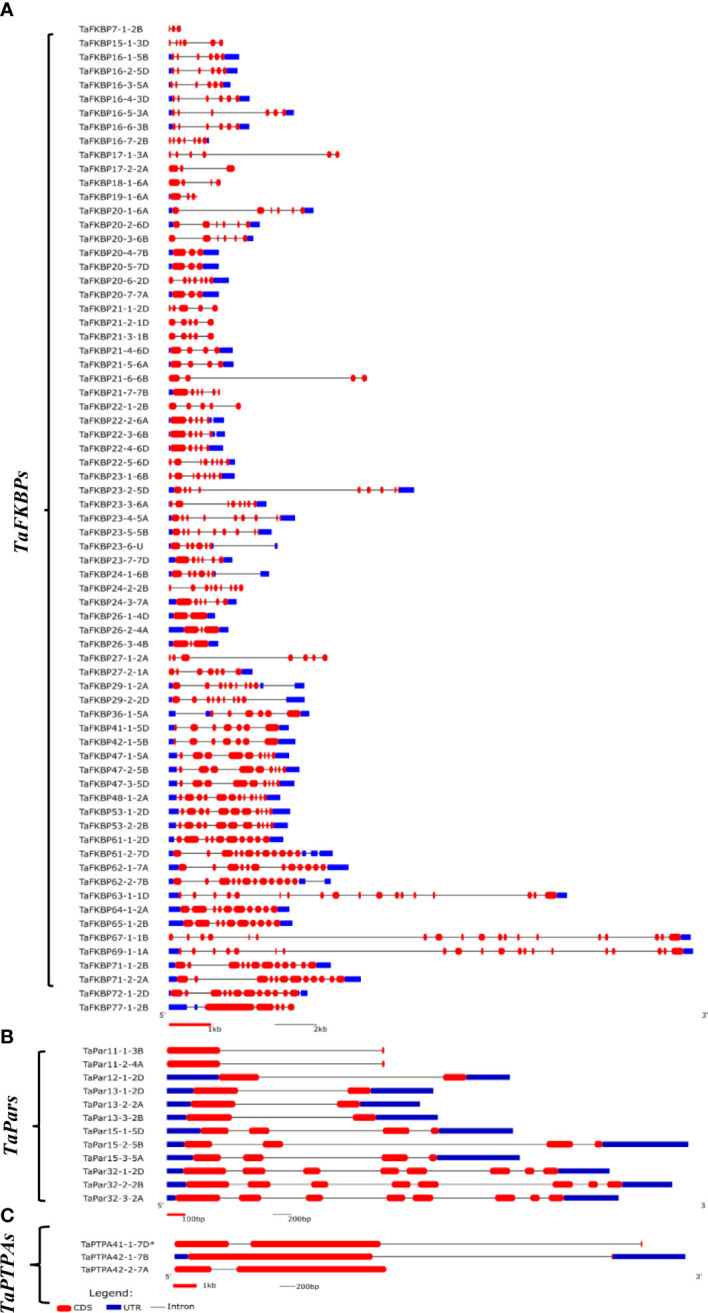
Structure of genes encoding FKBPs **(A)**, Parvulins **(B)** and PTPAs **(C)** in T. aestivum. * represents exact molecular weight and pI not determined.

Analysis of gene organization revealed significant variability in the exon-intron structure of different *TaFKBP, TaPar* and *TaPTPA* genes (**Figure 3**). Genes of all the three PPIase sub-families showed the presence of introns in their open reading frames (ORFs), with the number of introns varying significantly within each of the sub-families. For instance, the number of introns in the ORF of these genes ranges between 1-19 in *TaFKBPs*, 1-7 in *TaPars* and 1-2 in *TaPTPAs*. The genes encoding PTPAs and FKBPs exhibited homoeolog-dependent variability in the number of introns, as also observed earlier for the wheat cyclophilin genes (Singh et al., 2019). For example, *TaFKBP16-7-2B* and *TaFKBP20-6-2D* consist of six introns each in their ORFs compared to 2-10 in their other homeologues *TaFKBP7-1-2B*, *TaFKBP24-2-2B*, *TaFKBP29-1-2A* and *TaFKBP29-2-2D*. Similarly, *TaPTPA42-1-7B* and *TaPTPA42-2-7A* depicted a single intron in their ORFs, relative to two in the third homeolog, TaPTPA41-1-7D*. On the contrary, the *TaPar* genes showed conservation in the abundance of introns within members of the same homeologous groups. The length of introns also differs among different PPIase genes, with both the smallest (53 bp) and the largest (7738 bp) being observed in *TaFKBPs*, *TaFKBP15-1-3D* and *TaFKBP67-1-1B*, respectively. Within the *TaPTPA* gene family, the smallest (272 bp) and the largest (3302 bp) introns were observed in TaPTPA41-1-7D*. The homeologous group of *TaPar* genes, comprising of *TaPar32-1-2D*, *TaPar32-2-2B* and *TaPar32-3-2A*, depicted the smallest length (80 bp) for intron 4, while the largest (2833 bp) was observed in *TaPar15-2-5B*. While majority of the *TaFKBPs* (50) contain untranslated regions (UTRs) in both the 5’ and 3’ regions, the same could not be observed for 12 of these genes. Nine *TaFKBPs* exhibited introns only in their 3’ UTR and two in only 5’UTR. Absence of 5’ UTR or 3’ UTR was also observed among *TaFKBPs*. While 5’ UTR is lacking in six *TaFKBPs* (*TaFKBP16-7-2B*, *TaFKBP20-3-6B*, *TaFKBP20-6-2D*, *TaFKBP22-5-6D*, *TaFKBP27-2-1A* and *TaFKBP67-1-1B*), three *TaFKBPs* (*TaFKBP19-1-6A*, *TaFKBP21-7-7B*, *TaFKBP77-1-2B*) are devoid of 3’ UTR. Though UTR of several *TaFKBPs* depicted the presence of introns, none of the *TaPar* and *TaPTPA* genes exhibited this feature. The genes *TaPar11-1-3B*, *TaPar11-2-4A*, TaPTPA41-1-7D* and *TaPTPA42-2-7A* showed absence of both 5’ and 3’UTRs. Unlike the cyclophilin and FKBP gene families in lower organisms *viz., Leptosphaeria maculans* (Singh et al., 2014), that contain shorter introns and compact genomes, the length of introns is larger than exons in most of the *TaFKBP, TaPar* and *TaPTPA* genes.

### Domain architecture and active site residue analysis

**Figure 4:**
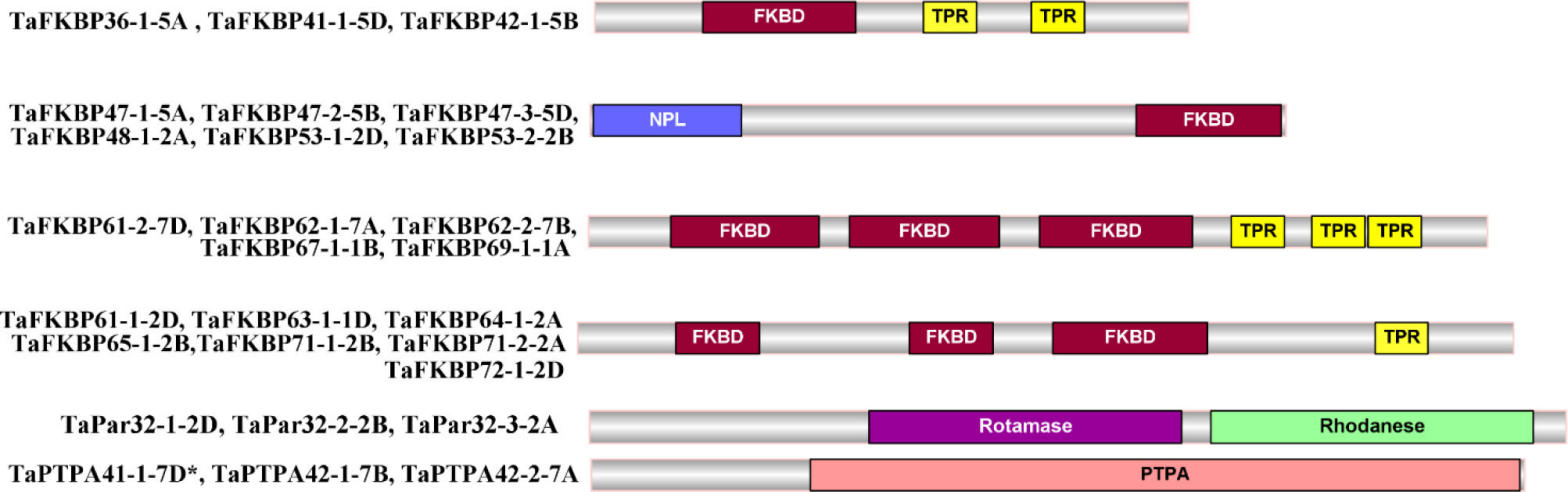
Schematic representation of multi-domain FKBP, parvulin and PTPA proteins of *T. aestivum*.

The FKBP, parvulin and PTPA proteins in hexaploid wheat contain the characteristic signature domains, with several members of former two families also containing additional domains (**Figure 4**). Unlike the TaFKBPs and TaPars, several of which are multi-domain proteins, the TaPTPAs consist of only the PTPA domain. Besides FKBD, 21 of the 71 TaFKBPs also demonstrated the presence of other domains, such as nucleoplasmin-like (NPL) and tetratricopeptide repeat (TPR) domain, implying diversification in their roles (**Supplementary Table S1**). Except for NPL domain-containing FKBPs, such as TaFKBP47-1-5A, TaFKBP47-2-5B, TaFKBP47-3-5D, TaFKBP48-1-2A, TaFKBP53-1-2D and TaFKBP53-2-2B, that exhibit FKBD at C-terminus, this domain in other multi-domain proteins is localized at the N-terminus. One of the single domain proteins, TaFKBP19-1-6A, contains a partial N-terminus FKBD. Twelve of the multi-domain FKBPs also contain TPR domain in addition to three FKBDs, indicating their likely role in protein-protein interactions. Since the NPL domain-containing FKBPs have been demonstrated to play an important role in chromatin remodelling and regulation of transcription in *A. thaliana* (Li and Luan, 2010), the presence of this domain in the wheat FKBPs (TaFKBP47-1-5A, TaFKBP47-2-5B, TaFKBP47-3-5D and TaFKBP48-1-2A, TaFKBP53-1-2D, TaFKBP53-2-2B) implies the role of these proteins in regulation of gene expression.

**Figure 5:**
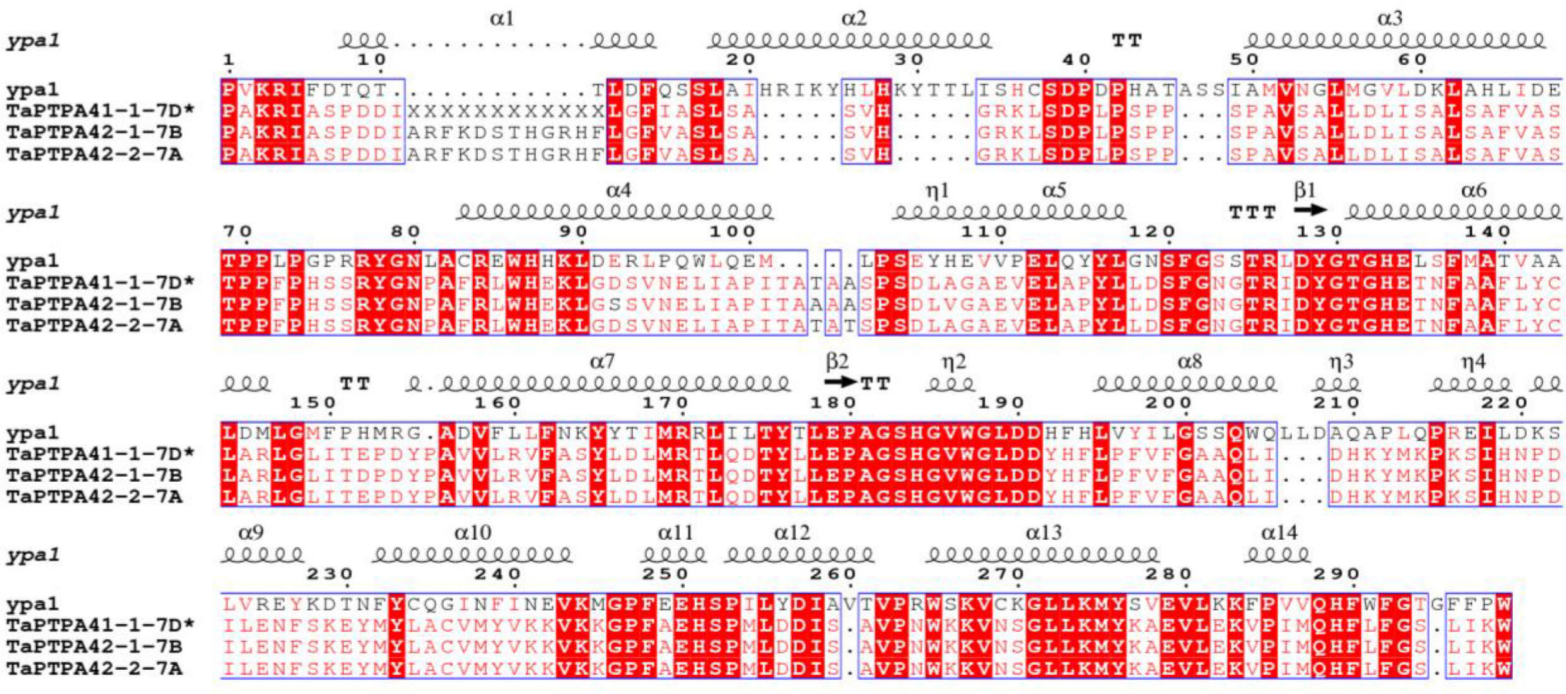
Multiple sequence alignment of PTPA domains of wheat PTPA proteins and yeast PTPA ypa1. * represents exact molecular weight and pI not determined.

Comparison of active site residues with the human FKBP, hFKBP12, revealed the absence of His (H-87) residue in all members of FKBP family in wheat (**Supplementary Table S8**; **Figure S1A**). Similarly, compared to their human counterpart hPar14, all wheat parvulins also lack the critical residue Asp (D-74; **Supplementary Table S9** and **Figure S1B**). Variability in other active site residues was also observed in TaFKBPs and TaPars. The parvulins TaPar11-1-3B (11.06 kDa) and TaPar11-2-4A (11.16 kDa) possess only two of the conserved active site residues i.e., His (H-42) and Ser (S-72), indicating that these proteins might be enzymatically inactive. This speculation, however, needs experimental validation. The higher molecular weight TaFKBPs, such as TaFKBP61-1-2D (61.35 kDa), TaFKBP61-2-7D (61.97 kDa), TaFKBP62-1-7A (62.01 kDa), TaFKBP62-2-7B (62.02kDa), TaFKBP63-1-1D (63.97 kDa), TaFKBP64-1-2A (64.89 kDa), TaFKBP65-1-2B (65.60 kDa), TaFKBP67-1-1B (67.96 kDa), TaFKBP69-1-1A (69.90 kDa), TaFKBP71-1-2B (71.30 kDa), TaFKBP71-2-2A (71.80 kDa) and TaFKBP72-1-2D (72.07 kDa), which contain multiple FKBDs, also depicted difference in the occurrence of active site residues in different FKBDs. This observation suggests that the FKBDs with the maximum number of conserved active site residues might be enzymatically active while others may not be functional. This speculation, though warrants experimental confirmation, is also supported by a previous study which showed that of the three FKBDs in the wheat FKBP, wFKBP73, only one of the domains was enzymatically active (Unger et al., 2010). Our analysis also revealed that the different FKBDs in TaFKBPs are conserved among different homeologues. High identity of more than 94.31% was also observed in PTPA domains of TaPTPA41-1-7D*, TaPTPA42-1-7B and TaPTPA42-2-7A (**Figure 5**), signifying conservation of these proteins.

### Motif analysis

**Figure 6:**
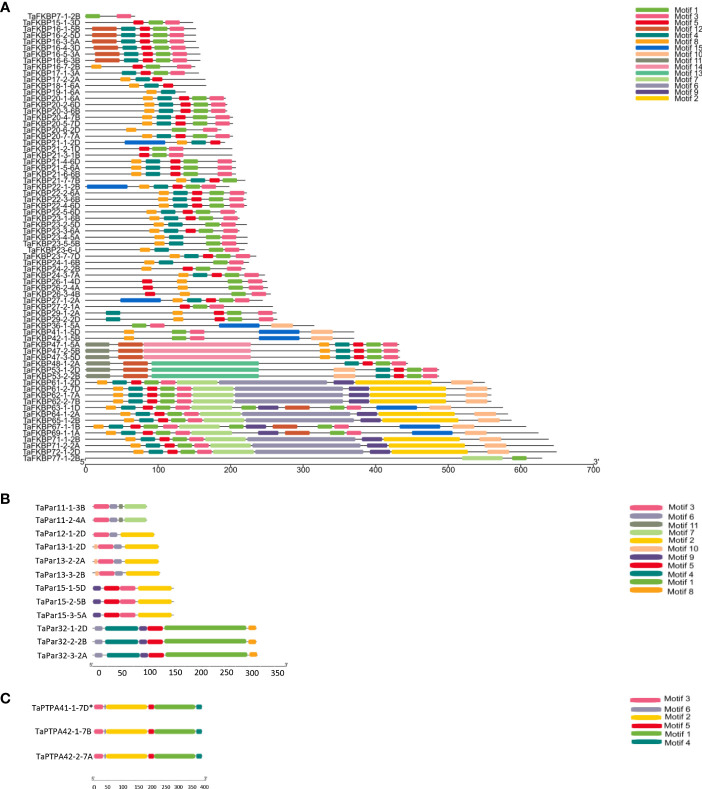
Organization of different conserved motifs in FKBP **(A)**, parvulin **(B)** and PTPA **(C)** proteins of *T. aestivum*.

Protein sequences of TaFKBPs, TaPars and TaPTPAs were also analysed for the presence of conserved motifs. A total of 15 motifs, varying in length from 15 to 150 amino acids, were identified in TaFKBPs (**Table 1**; **Figure 6A**). The FKBD comprises of motifs 1, 3, 4, 5, 6 and 7, with the motifs 1, 3, 4 and 5 being observed in maximum number of proteins (67, 65, 56 and 59, respectively). The motifs 1, 3 and 4 consist of 21 amino acid residues each, compared to 15 residues in motif 5, with all constituting a component of FKBD. The absence of motifs 6 and 7 in TaFKBPs might be due to deletion of these sequences in their C-termini. Presence of only two motifs (1 and 7) in the highest molecular weight protein TaFKBP77-1-2B (77.10 kDa), might be due to the presence of partial FKBD in this protein. Apart from the conserved FKBDs, *in silico* analysis also predicted the presence of motifs in TPR (motifs 2 and 15) and NPL domains (motifs 11 and 12) of the multi-domain FKBPs. The motifs 1, 3, 4, 5 (FKBP_C) and 8 (unknown function) were observed most commonly, with 36 TaFKBPs exhibiting the presence of this combination. However, exclusive presence of motif 13 (ASP_Protease) and motif 14 (Domain of unknown function) in two different homeologue triplets i.e., TaFKBP48-1-2A, TaFKBP53-1-2D and TaFKBP53-2-2B; and TaFKBP47-1-5A, TaFKBP47-2-5B and TaFKBP47-3-5D, respectively, might be associated with some discrete functions of these proteins, that are yet to be elucidated. In another homeologue triplet, TaFKBP24-1-6B, TaFKBP23-6-U and TaFKBP19-1-6A, the former two proteins harbour motifs 1, 3, 4 (FKBP_C) and 8 (unidentified motif), while TaFKBP19-1-6A consists of only motifs 4 and 8. The presence of other motifs such as LBR tudor, cytidylate kin 2, ASP_Protease, domain of unknown function and CDC45-like protein in few of the wheat FKBPs suggests novel cellular roles that are yet to be studied. Akin to TaFKBPs, homeolog-specific variations in motifs were also observed in TaPars (**Table 1**; **Figure 6B**). On the contrary, all the three PTPAs, TaPTPA41-1-7D*, TaPTPA42-1-7B and TaPTPA42-2-7A, exhibited the same motif architecture (**Table 1**; **Figure 6C**).

**Table 1:** Comparative analysis of different conserved motifs in FKBP, parvulin and PTPA proteins in *T. aestivum*.

Motif	Motif sequence	Amino acids	Putative function (Pfam/Prosite/CDD)	Proteins having this motif
**TaFKBPs**				
1	IKGMKVGEKRRLTIPPELGYG	21	FKBP_C	67
2	NNAEKIEAAAKKKDEGNAWFKMGKYARASKRYEKAAKFIEYDSSFSEDEKKQSKPLKISCKLNNAACKLKLKDYKEAEKLCTKVLELDSTNVKALYRRAQAYTELV	106	TPR1/TPR2	9
3	GSPPEIPPNATLIFDVELLSV	21	FKBP_C	65
4	GDKVEVHYTGTLADGTVFDSS	21	FKBP_C	56
5	PFKFRLGSGQVIKGW	15	FKBP_C	59
6	HFCPALSKAVKTMKKGEKVLLTVKPQYGFGEQGRPASEDEGAVPPNATLHIDLZLVSWKTVTEIGDDKKILKKVLKEGEGYERPNDGAVVKVKLIGKLDDGTVFVKKGHDGQEPFEFKTDEEQVIEGLDRAVLTMKKGEVALVTIPPEHA	150	FKBP_C	9
7	SVKDICKDGGIFKKILKEGEKWENPKDPDEVTVKYEARLEDGTVVSKSEGVEFTVKD	57	FKBP_C	12
8	TKSGLKYKDLKVGEG	15	–	55
9	DLAVVPPNSTVYYEVELVSFDKEKESWDL	29	LBR tudor	12
10	KKLKEKVKEYNKKDAKFYKNMFNKKPKPENE	31	cytidylate kin 2	17
11	SAFWGVEVKPGKPYTHSHNPRHGRLRJTQATLGA	34	NPL	6
12	VVGNKEPVLLCALAPKLADVCHLQIELEEKPEVFS	35	NPL	14
13	RSVHLAGYYVGDVYEDIGDSDTGSESLQGSDDDFLASDDDDVVIPVSHGQMNTDSEDDSDYDEDYDSEDDEDLMYNQGRGKSSVVIEEIQEDEKPVDDNSRLQLAVRTPPAESVESEDEDGFPVSESKKSSKGSSKKDKNLNNGTSTED	149	ASP_Protease	3
14	SVLGQSSVHLSGYYLRPGSRGNAGEEDSESYGEDVGESDTDQDYEGSEDSYESDFIDDGDNEVPEDSDVSDSMDDGDVCSTPDHRKQDSEKHARKVKRQRRLKKKQQVDSSADKIADSPSKPAARRKRGSIFDSASEDEDFLAQSEEEN	149	Domain of unknown function/CDC45 like protein	3
15	HLNVAACLJKQPRFDLAIAACAKVLTENPVNVKALYRRGKAYAEAGRAEDAKEDFLK	57	TPR/domain of unknown function	9
**TaPars**				
1	CPSKENGGMLGWVRRGQMVPEFEEAAFSAPLNKVVRCKTKFGWHLVQVLSERDQCLLQDIQPEELHEKMQDPSFIEEAQLIDVREPDEVERASLPGFKVLPLRQFGTWGPVMTDEFNPQKDTYVLCHHGMRSMQVAKWLQSQGFQKIYNV	150	PPIC-type PPIase domain, Rhodanese domain	3
2	EFADVAQZHSDCPSAKRGGDLGTFPRGKMQKPFZEAAYALKVGEISDIIDTESGVHIILRTG	62	PPIC-type PPIase domain	7
3	ASHILIKHEGSRREASWKDPEGRVISATT	29	PPIC-type PPIase domain	9
4	SLSGLARRAPLLAVASPAPSSPAALSLLASARPVSAAWGSAMRPAGEHPRPGTRVLCTAAS	61	–	3
5	DGEGGAKGGKGKGKGGKGGDDLGTLAVEK	29	–	6
6	RADAAARLGELRPQI	15	–	9
7	QHSARPPAAGLHQRADHARQIQDCLVMDKINPCGQQYWYQC	41	–	2
8	IHAYSVKADSSIPTY	15	–	3
9	GKPGEGKEVKGKGKL	15	–	6
10	TGEETVR	7	–	3
11	DCASFADL	8	–	2
12	CRQFGA	6	–	2
**TaPTPAs**				
1	NGTRIDYGTGHETNFAAFLYCLARLGLITEPDYPAVVLRVFASYLDLMRTLQDTYLLEPAGSHGVWGLDDYHFLPFVFGAAQLIDHKYMKPKSIHNPDILENFSKEYMYLACVMYVKKVKKGPFAEHSPMLDDISAVPNWKKVNSGLLKM	150	PTPA	3
2	WTGDSPPPAYRPIRMPAINAPTNTAAIVLSPVPQPLPVPPASPPFAFQAPAKRIASPDDIARFKDSTHGRHFLGFVASLSASVHGRKLSDPLPSPPSPAVSALLDLISALSAFVASTPPFPHSSRYGNPAFRLWHEKLGDSVNELIAPIT	150	PTPA	3
3	MSNPESSPQAAASSTSPPSHAGHIHTPLCRSCG	33	–	3
4	EVLEKVPIMQHFLFGSLIKWE	21	PTPA	3
5	SPSDLAGAEVELAPYLLDSFG	21	PTPA	3
6	APAPAP	6	–	3

The different motifs were identified using MEME tool.

### Phylogenetic analysis of wheat FKBP, parvulin and PTPA proteins

**Figure 7:**
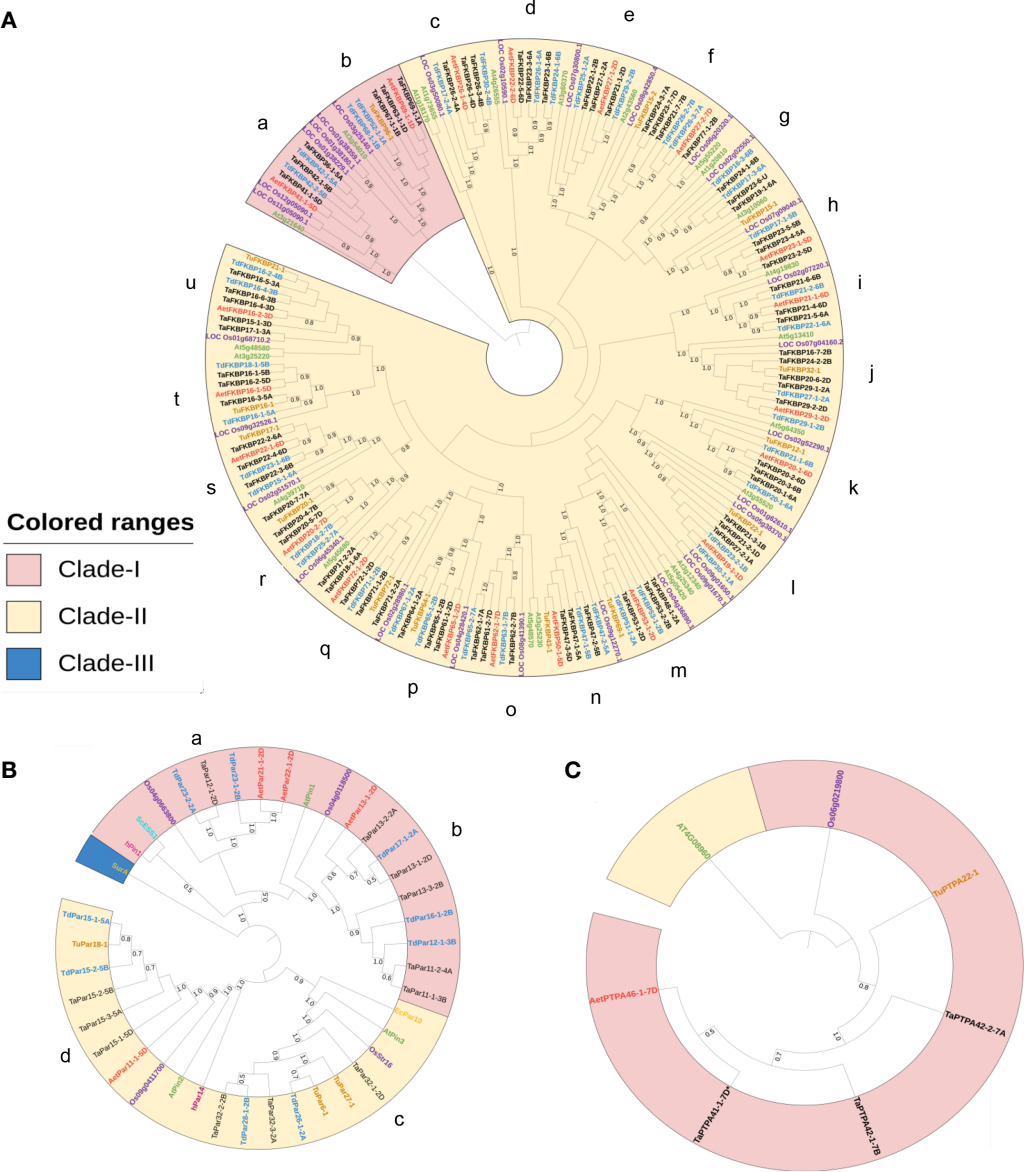
Phylogenetic analysis of FKBP **(A)**, Parvulin **(B)** and PTPA **(C)** proteins of *T. aestivum* (black), *T. urartu* (orange), *T. dicoccoides* (blue), *A*. *tauschii* (red), *A*. *thaliana* (green) and *O. sativa* (purple), *H*. *sapiens* (pink), *S. cerevisiae* (blue) and *E*. *coli* (yellow). Clustal Omega was used to perform the multiple sequence alignment. The phylogenetic tree was constructed by Neighbor-Joining (NJ) method of MEGA X standalone application with 1000 bootstrap replicates. The generated tree was annotated using iTOL online server. Different clades are highlighted with different colors.

To understand evolutionary relationships among FKBPs, parvulins and PTPAs of different plant species, we constructed separate phylogenetic tree for each of these protein families using alignments of amino acid sequences from wheat, its progenitors, and other plant species. For FKBPs, the phylogenetic tree constructed using NJ method in MEGA X was based on alignment of 196 FKBD-containing proteins from *T. aestivum* (70), *T. urartu* (14), *T. dicoccoides* (40), *A. tauschii* (20), *A. thaliana* (23) and *O. sativa* (29) (**Figure 7A**). Similarly, for parvulins also, the phylogenetic tree included 40 different parvulin protein sequences from *T. aestivum* (12), *T. urartu* (3), *T. dicoccoides* (9), *A. tauschii* (4), *A. thaliana* (3), *O. sativa* (4), *H. sapiens* (2), *S. cerevesiae* (1) and *E*. *coli* (2) (**Figure 7B**). For PTPAs, a total of seven proteins from *T. aestivum* (3), *T. urartu* (1), *A. tauschii* (1), *A. thaliana* (1) and *O. sativa* (1) were included for constructing the phylogenetic tree (**Figure 7C**).

Phylogenetic analysis classified the TaFKBPs into two different clades (Clades-I and II), which were further divided into various sub-groups consisting of homologous FKBPs from different plant species. Clade-I consisted of 21 members compared to 175 in Clade-II. The ultimate sub-groups (labeled a to u) comprised all the TaFKBP homeologue triads that were clustered together with their progenitor species and homologues from other plant species. The close clustering of homeologue triad was also observed for cyclophilins and 14-3-3 gene families in common wheat (Singh et al., 2019; Shao et al., 2021). While the sub-groups a, c, d, e, g, i and o lacked FKBP homologues from *T. urartu* (A genome), the sub-groups h, q and u showed the absence of FKBP homologues from *T. dicoccoides* (A genome). The clustering pattern depicted in the phylogenetic tree prompts us to hypothesise that the *FKBP* genes on chromosomes 1A, 2A, 4A, 5A, 6A and 7A of the common wheat either evolved from *T. dicoccoides,* or the corresponding genes present in *T. urartu* were later lost during the course of evolution. Conservation of most of the homeologue triplets implies that members of the *TaFKBP* gene family were not subjected to significant changes during the course of evolution and these genes in hexaploid wheat have been acquired from its progenitors due to polyploidy. The presence of FKBP homologues from *O. sativa* (monocot) and *A. thaliana* (dicot) in each of the sub-groups implies existence of these genes even before divergence of monocots and dicots. Furthermore, the grouping of FKBPs appears to be domain specific and also influenced by the localization pattern, and the arrangement of introns and exons in the corresponding genes. For example, Clade-I consisted of multi-domain FKBPs possessing TPR and FKBDs. One of its two sub-clades contained proteins with three FKBDs and one TPR domain, with their localization predicted to be in the nucleus. However, all other members with similar domain architecture, but localized to cytosol, formed a separate sub-clade in Clade-II. Members containing multiple domains formed different sub-groups in Clade-II (for example sub-groups m, n, o, p, q). On the other hand, most of the FKBPs consisting of only a single FKBD were clustered together based on their localization pattern. This implies that the localization signal (**Supplementary Table S1**) and conservation of the underlying structure (**Figure 3A**) might have played an important role in the functional divergence of FKBPs in common wheat.

The phylogenetic tree of TaPars depicted three major clades *viz.*, Clades-I, II and III. The Clades I and II comprised of 19 and 20 members, respectively, while the third clade contained only a single parvulin, surA, from *E. coli*, and was differentiated from other two clades that consisted of various eukaryotic parvulins. Both Clade-I and Clade-II were further divided into sub-clades (labeled a-d), with all the sub-groups, except sub-group a that consisted of only a single member TaPar12-1-2D from D genome of *T. aestivum*, represented by triplet homeologues. The sub-group b consisted of two additional proteins from *T. aestivum* (TaPar11-1-3B and TaPar11-2-4A), encoded by a gene each on chromosomes 3B and 4A. Except for sub-group d, none of the other sub-groups depicted the presence of members representing each of the three wheat sub-genomes. For example, sub-groups a and b lacked representatives from *T. urartu*, while sub-group c was devoid of corresponding homologue from *A. tauschii*. Computational analysis categorised the PTPA proteins into two groups, Clades-I and II, with the former comprising of closely clustered PTPA proteins from different species (*T. aestivum*, *T. urartu*, *A. tauschii* and *O. sativa*), while Clade-II included PTPA from dicot *A. thaliana*. These observations suggest that in contrast to *FKBP* genes, the *PTPA* genes might have diverged after differentiation of monocots from dicots.

### Expression analysis of *TaFKBP*, *TaPar* and *TaPTPA* genes in response to heat stress

**Figure 8:**
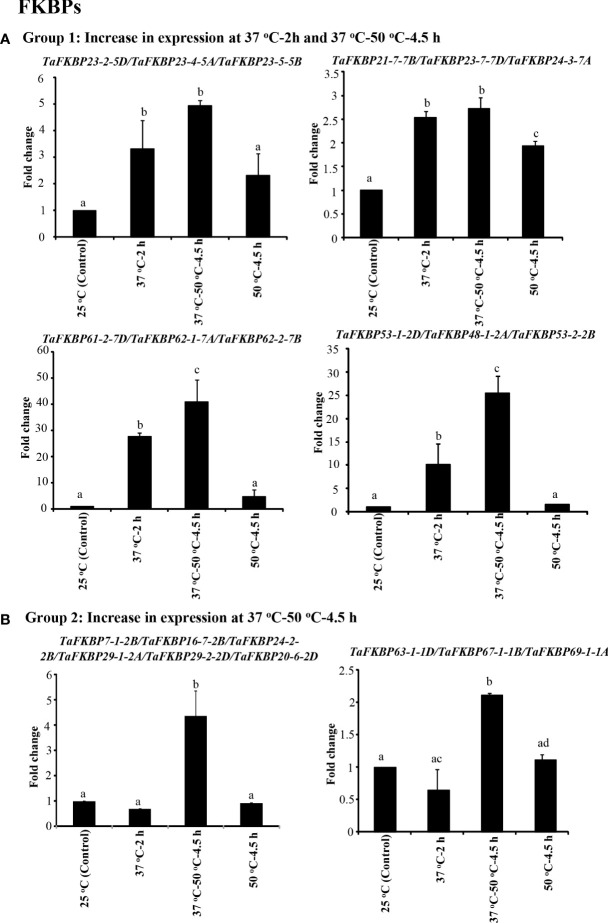
qRT-PCR analysis of *FKBP* genes in the seven days old wheat seedlings. **(A)** Genes whose expression was induced during acclimation phase (37°C-2 h) and was maintained for further 4.5 h on subsequent exposure to 50 °C. **(B)** Genes that registered an increase in expression only at 37 °C-50 °C-4.5 h. *Actin* was used as reference. The values depict the mean of three biological replicates ± standard error and the different lowercase letters denote significant difference between the treatments at P ≤ 0.001 (Tukey-HSD test; α=0.05) (/represents homeologs).

**Figure 9:**
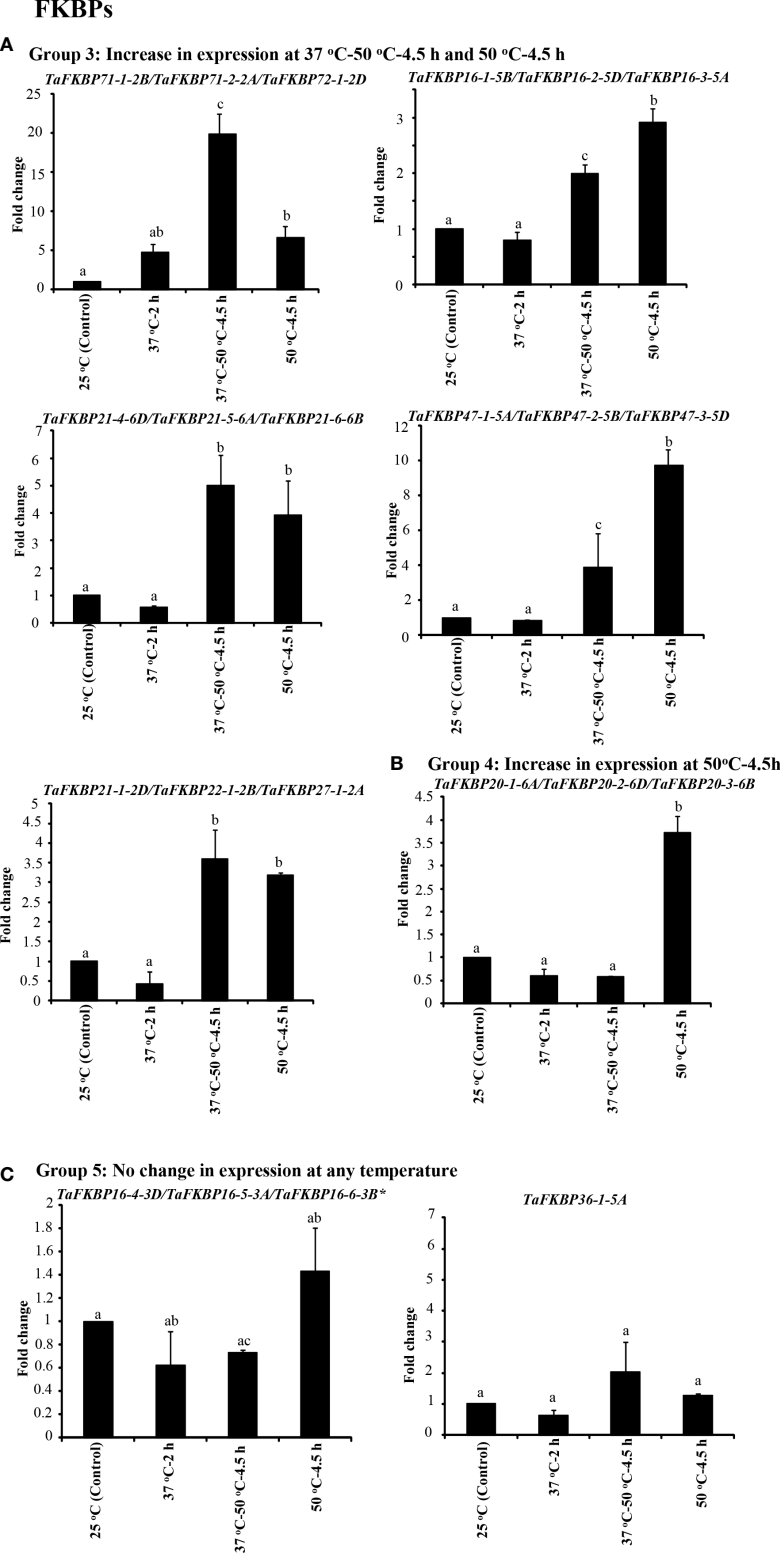
qRT-PCR analysis for studying the effect of heat stress on expression of *TaFKBP* genes in the seven days old wheat seedlings. **(A)** Genes showing upregulation in response to both direct heat stress (50 °C) and after acclimation (37 °C-50 °C-4.5 h), **(B)** only direct heat stress (50 °C) and **(C)** unaffected by heat stress. *Actin* was used as reference. The values depict the mean of three biological replicates ± standard error and the different lowercase letters denote significant difference between the treatments at P ≤ 0.001 and P ≤ 0.05* (Tukey-HSD test; α=0.05) (/represents homeologs).

**Figure 10:**
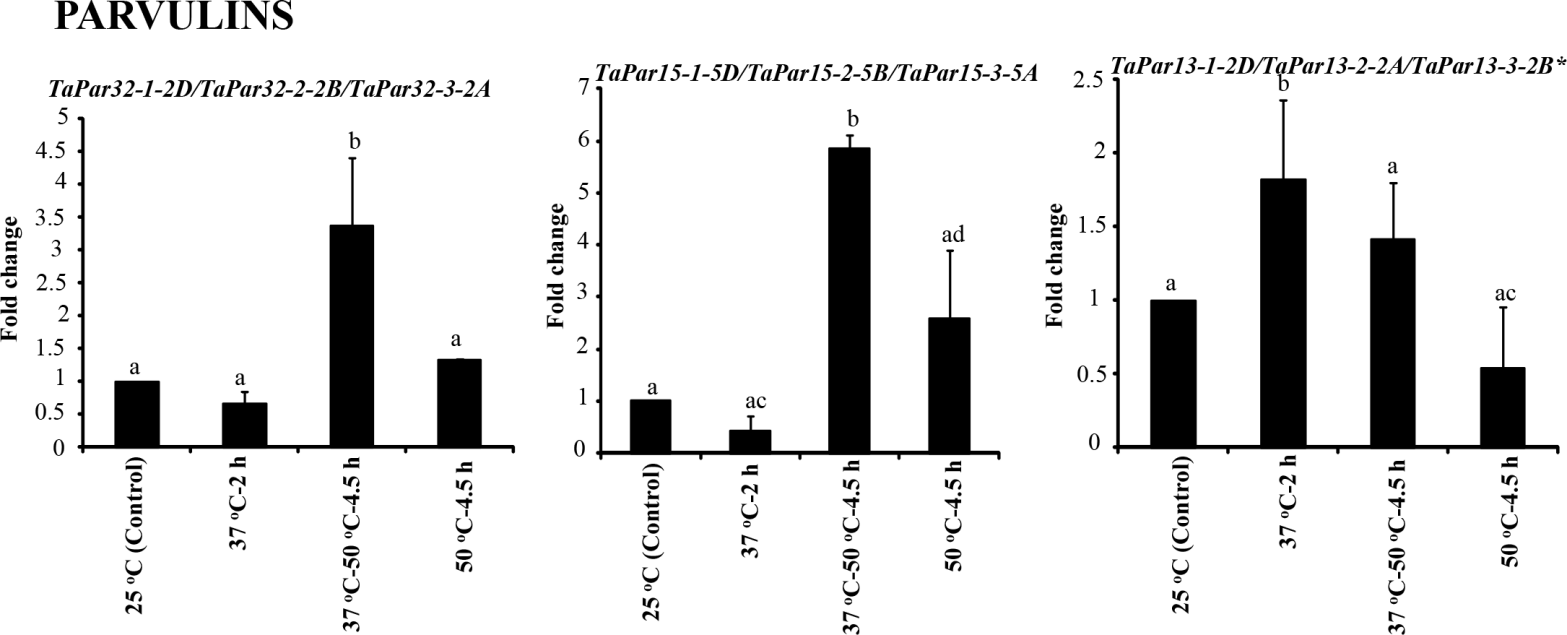
Effect of heat stress on expression of genes encoding parvulins in seven days old wheat seedlings using *Actin* as reference. The values depict the mean of three biological replicates ± standard error and the different lowercase letters denote significant difference between the treatments at P ≤ 0.001 and P ≤ 0.05* (Tukey-HSD test; α=0.05) (/represents homeologs).

The effect of heat stress on expression of *TaFKBP*, *TaPar* and *TaPTPA* genes was investigated in response to various heat stress treatments using qRT-PCR (**Figures 8**–**10**). The survivability of the wheat seedlings was followed, as described earlier, after exposure to lethal temperature (50 °C) without and with acclimation of seedlings at 37 °C for 2 h (Singh et al., 2019). The seedlings that were acclimated at 37°C for 2 h before heat stress at 50°C for 4.5 h showed higher survival (80%) as compared to the seedlings that were exposed to lethal heat stress at 50°C without acclimation (19%), indicating acquisition of thermotolerance at sub-lethal temperature (Singh et al., 2019). For expression analysis, common primers for 28 different homeolog *TaFKBP* groups (71 genes), five for *TaPars* (12 genes) and a single primer pair for the *TaPTPAs* (3 genes) were designed and validated by conventional PCR. Of these, 14 and nine primer pairs, representing 43 and nine different *TaFKBPs* and *TaPars*, respectively, from different homeolog groups depicted amplification and were used further for qRT-PCR analysis (**Supplementary Table S10**). The *TaPTPA* genes, however, did not show amplification.

On the basis of expression profile, the *TaFKBP* genes were classified into five different groups (**Figures 8**, **9**). The expression of Group 1 genes, except *TaFKBP53-1-2D/TaFKBP48-1-2A/TaFKBP53-2-2B* and *TaFKBP61-2-7D/TaFKBP62-1-7A/TaFKBP62-2-7B*, after increasing during acclimation (37 °C for 2 h) was also maintained under subsequent lethal heat stress (37 °C-50 °C-4.5 h), indicating the likely role of these genes in thermotolerance (**Figure 8A**). Among the Group 1 genes, highest expression after acclimation (27.7-fold) and in response to subsequent lethal heat stress (40.8-fold) was observed for *TaFKBP61-2-7D/TaFKBP62-1-7A* and *TaFKBP62-2-7B*, respectively. On the contrary, *TaFKBP21-7-7B/TaFKBP23-7-7D* and *TaFKBP24-3-7A* registered the lowest increase in transcript level after acclimation (2.5-fold) and at 37 °C-50 °C-4.5 h (2.7-fold). However, these genes were the only members of Group 1 which depicted significantly higher expression compared to control 25 °C when exposed to direct heat stress at 50 °C for 4.5 h (**Figure 8A**).

The expression of Group 2 genes, compared to control, was enhanced significantly only when the thermal stress (50 °C-4.5 h) was imposed after acclimation (**Figure 8B**). Exposure to acclimation temperature (37 °C-2 h) and direct heat stress had no appreciable effect on the expression of these genes (**Figure 8B**). The genes that depicted an increase in expression in response to both direct heat stress and after acclimation were categorised in Group 3 (**Figure 9A**). Among the Group 3 genes, the increase in transcript abundance by heat stress at 50 °C ranged from 2.9-fold (*TaFKBP16-1-5B/TaFKBP16-2-5D/TaFKBP16-3-5A*) to 9.7-fold (*TaFKBP47-1-5A/TaFKBP47-2-5B/TaFKBP47-3-5D*) without acclimation, and from 1.9-fold (*TaFKBP16-1-5B/TaFKBP16-2-5D/TaFKBP16-3-5A*) to 19.9-fold (*TaFKBP71-1-2B/TaFKBP71-2-2A/TaFKBP72-1-2D*) after acclimation (37 °C-50 °C-4.5 h). The expression of these genes was not affected by sub-lethal temperature (37 °C for 2 h).

Contrary to Group 1, 2 and 3, the Group 4 comprised a single *TaFKBP* triplet, *TaFKBP20-1-6A/TaFKBP20-2-6D/TaFKBP20-3-6B*, that showed increase in transcript level (3.7-fold) only in response to direct exposure to 50 °C but was unaffected by any other temperature regime (**Figure 9B**). The genes *TaFKBP16-4-3D/TaFKBP16-5-3A/TaFKBP16-6-3B* and *TaFKBP36-1-5A*, whose expression was not responsive to any of the temperature treatments, were grouped into Group 5 (**Figure 9C**). Expression analysis of *TaPar* genes revealed that compared to 25 °C control, the transcript levels of *TaPar32-1-2D/TaPar32-2-2B/TaPar32-3-2A* and *TaPar15-1-5D/TaPar15-2-5B/TaPar15-3-5A* were upregulated by heat stress after acclimation (37 °C-50 °C-4.5 h), but were not responsive to direct heat stress (50 °C for 4.5 h) (**Figure 10**). On the contrary, the expression of *TaPar13-1-2D/TaPar13-2-2A/TaPar13-3-2B* was enhanced only during acclimation (37 °C for 2 h), but was not affected by direct thermal stress.

**Figure 11:**
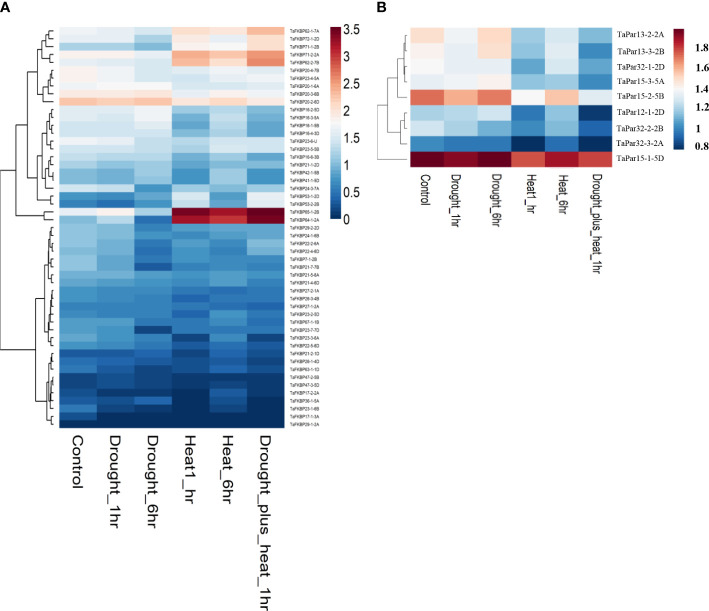
Heatmap showing expression of wheat FKBP **(A)** and parvulin **(B)** encoding genes under heat and drought stress conditions. Expression values were derived from the data available at WheatExp database (https://wheat.pw.usda.gov/WheatExp/) pertaining to treatment of one week old seedlings with heat stress at 40°C, drought or both for 1 h and 6 h duration (Liu Z et al., 2015). The scaled color gradient varies from blue (downregulation) to red (upregulation).

Expression analysis of *TaFKBPs* and *TaPars* was also performed digitally under heat (40 °C for 1 h and 6 h) and drought stress conditions using RNAseq expression data (**Figure 11**; Liu Z et al., 2015). Heatmap analysis revealed differential modulation of several members of the *TaFKBP* gene family under heat stress. Of the 71 different *TaFKBP* genes, *TaFKBP64-1-2A* and *TaFKBP65-1-2B* showed substantial upregulation at 40 °C over control (**Figure 11A**). The significant increase in expression of *TaFKBP62-1-7A/TaFKBP62-2-7B* during acclimation phase (37 °C-50 °C-4.5 h) and their subsequent downregulation at higher temperature (50 °C-4.5 h), as observed in our study, is also supported by heatmap analysis which showed upregulation of these genes only when exposed to 40 °C for only 1 h, with prolonged stress for 6 h resulting in a decrease (**Figure 11A**). Induction of Group 3 genes *TaFKBP71-2-2A/TaFKBP72-1-2D* (**Figure 9A**) during acclimation is also supported by digital expression analysis, which showed higher expression after heat stress. Similarly, decrease in expression of *TaPar13-2-2A* and *TaPar13-3-2B* genes in response to direct heat stress (50 °C) is also consistent with the results of digital analysis that revealed downregulation of these genes at 40 °C (**Figure 11B**). Difference in the expression profile of several other genes, such as *TaFKBP20-1-6A/TaFKBP20-2-6D/TaFKBP20-3-6B*, observed between the present study and digital analysis, may be ascribed to difference in the temperature regime and the genotype used.

Heat shock elements have been implicated in regulation of genes by thermal stress (Schὂffl et al., 1998). Therefore, to understand the role of cis-regulatory elements in the stress-responsiveness of different genes, 2.0 kb upstream regions of these genes were examined for the identification of heat stress responsive elements (**Supplementary Table S11**). These analyses resulted in identification of different heat shock elements (HSEs), such as stress response element (STRE), TTC rich type 1, TTC rich type 2, TTC rich type 3, TTC rich type 4, Gap type 1, Gap type 2, Gap type 3 and Perfect HSE (Qazi et al., 2020), which with the exception of few members, were observed in varying number (0-23) in the upstream regions of all genes. The perfect HSEs (Shakeel et al., 2011) were identified in the upstream regions of only seven *TaFKBP* genes (TaFKBP20-1-6A, TaFKBP22-1-2B, TaFKBP23-5-5B, TaFKBP61-1-2D, TaFKBP77-1-2B, TaPTPA41-1-7D* and *TaPTPA42-2-7A*), out of which significant heat-induced increase in expression was observed only for *TaFKBP20-1-6A* (Group 4) on exposure to direct heat stress at 50 °C, and for *TaFKBP22-1-2B* (Group 3) at both 37 °C-50 °C-4.5 h and 50 °C-4.5 h (**Figure 8**). Similarly, the increase in expression observed at 37 °C-50 °C-4.5 h for the *TaPar* homeolog triplet *TaPar15-1-5D*/*TaPar15-2-5B* and *TaPar15-3-5A* could be due to the presence of HSEs present as STREs. Though the Group 5 genes did not show any change in transcript levels under any of the heat stress conditions, the upstream region of these genes also showed the presence of stress responsive cis elements, suggesting their likely role under different stress conditions.

### Protein-protein interaction network

**Figure 12:**
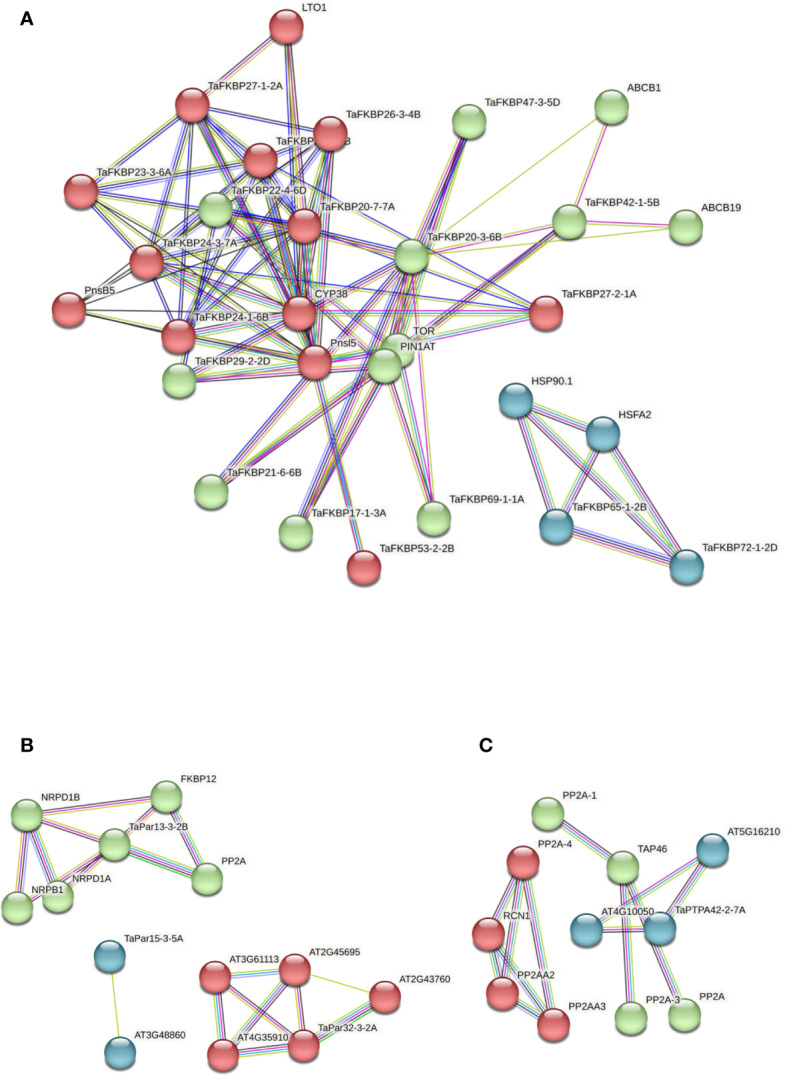
Protein-protein interaction (PPI) networks of wheat FKPBs **(A)**, parvulins **(B)**, and PTPAs **(C)**, constructed using the STRING database version 11.5 (https://string-db.org/; Szklarczyk et al., 2019). The clusters obtained using k-means clustering in each of the protein family are colored in red, green and blue. The network nodes represent proteins and the edges represent associations. The color of the edge line indicates the type of evidence i.e., violet: fusion, green: neighborhood, blue: cooccurrence, purple: experimental, yellow: text mining, light blue: database, black: coexpression.

The PPI networks of TaFKPBs, TaPars and TaPTPAs (**Figure 12**) were constructed using the STRING database (Szklarczyk et al., 2019). In total, TaFKBPs matched to 19 distinct *A. thaliana* orthologs, with identities ranging from 34.9 to 91.1% (**Supplementary Table S12**). As observed, the PPI network of FKBPs consisted of 29 nodes and 170 edges (**Figure 12A**), with the former showing varied degree of interaction ranging from 2 to 23 (**Supplementary Table S13**). Among TaFKBPs, the highest degree of interaction (19) was exhibited by TaFKBP20-3-6B and TaFKBP20-7-7A, while the lowest (6) was shown by TaFKBP47-3-5D and TaFKBP53-2-2B, suggesting that the FKBP proteins were highly linked with other proteins and are likely to play important roles in different biological processes. The k-means clustering grouped all the interacting partners into three clusters of size 13, 12 and 4 (**Supplementary Table S14**). The 12 TaPars matched to 3 distinct *A. thaliana* orthologs, with identities of 47.5 to 90.6% (**Supplementary Table S12**). The PPI network of TaPars (**Figure 12B**) consisted of 13 nodes and 24 edges, and the degree of interaction of nodes varied from 1 to 6 (**Supplementary Table S13**). The highest degree of interaction (7) among these proteins was exhibited by TaPar15-3-5A, and the lowest (4) by TaPar32-3-2A. The k-means clustering grouped all the interacting partners into three clusters of size 13, 12 and 4 (**Supplementary Table S14**). On the other hand, the three TaPTPA proteins matched a single *A. thaliana*, homologue with identity ranging from 57.8 to 59.1% (**Supplementary Table S12**). The PPI network of PTPAs (**Figure 12C**) consisted of 11 nodes and 47 edges with varied degree of interaction (**Supplementary Table S13**). TaPTPAs were clustered into three groups containing 3 to 4 members. As observed for TaFKBPs and TaPars, the PPI network of TaPTPAs also formed clusters with interacting partners, indicating their probable functions in the biological systems (**Supplementary Table S14**).

## Discussion

The FKBPs, parvulins and PTPAs are imperative for *cis-trans* isomerisation of the peptidyl-prolyl bonds, and several studies have revealed their essential role in diverse cellular processes (Hanes et al., 1989; Luan et al., 1994; Lu et al., 1996; Janssens and Goris, 2001; Van Hoof and Goris, 2003; Gopalan et al., 2004; Meiri et al., 2010; Yu et al., 2012; Singh et al., 2021). The availability of whole genome sequences has enabled detailed *in silico* analysis and characterization of these gene families in different plant species. Though previous studies characterized FKBPs in several plants (He et al., 2004; Gollan and Bhave, 2010; Wang W et al., 2012; Zhang X et al., 2014; Dong et al., 2018; Waseem et al., 2018), information on this important family of genes, until this study, was lacking in wheat. The present analysis revealed that the hexaploid wheat genome encodes for 71 different *TaFKBP* genes, which are unevenly distributed on the 21 different chromosomes. On the contrary, the *TaPTPA* and *TaPar* genes are confined to a single or only few chromosomes, respectively. The expansion of *FKBP* gene family in wheat appears to be the result of different duplication events, such as whole genome, segmental and tandem duplications. Synteny analysis indicated the presence of many collinear blocks of *TaFKBP* genes in wheat and its sub-genome donors. In addition, the presence of triplet homeologues for most of the *TaFKBPs* suggest that whole genome duplication due to polyploidization is likely the major force behind evolution of *FKBP* gene family in wheat, which was also reported for cyclophilin, 14-3-3 and invertase genes (Singh et al., 2019; Shao et al., 2021; Wang et al., 2021). However, compared to their corresponding sub-genome donors, the difference in prevalance of *FKBP* genes on some hexaploid wheat chromosomes indicates either a gain due to duplication or loss during the course of evolution (Shao et al., 2021).

Phylogenetic analysis showed strong clustering of homoeologue triplets of TaFKBP, TaPar and TaPTPA proteins, with overall grouping influenced by localization patterns and arrangement of sequence motifs. The monocot and dicot homologues of FKBPs and parvulins were clustered together in each of the sub-groups, indicating the existence of these genes in plants even before divergence of the two (**Figures 7A, B**). Similar observation was also reported for the invertase gene family in common wheat (Wang et al., 2021). On the contrary, the monocot and dicot PTPAs were clustered separately, indicating the acquisition of these genes by wheat genome after differentiation of dicots and monocots. Structural analysis of the genes revealed that the length of exons, which is variable in *TaFKBP* genes, could be related to their orthologs. This is evident, since the length of exon 3 (34 bp), 4 (62 bp) and 5 (53 bp) in homeologues *TaFKBP20-1-6A*, *TaFKBP20-2-6D*, and *TaFKBP20-3-6B* is similar to the length of exon 2, 3 and 4 in their *A. thaliana* ortholog *AtFKBP12* (Dong et al., 2018). Further, the length of these exons is also consistent with their other orthologs in *B. rapa, O. sativa, P. persica, S. lycopersicum* and *M. domestica* (Dong et al., 2018). The uniform pattern of exons was also observed for all the *TaPar* genes, and it corresponds with their *A. thaliana* orthologs *AtPIN1*, *AtPIN2* and *AtPIN3* (He et al., 2004). As reported for *FKBP* genes of *S. lycopersicum* (Waseem et al., 2018), considerable diversity was also noticed in the 5’ and 3’ UTR regions of *TaFKBPs*.

As observed earlier for cyclophilin proteins in wheat, the FKBPs also show variability in their domain architecture and localization, implying functional diversification (Singh et al., 2019). The presence of TPR domains in majority of the multi-domain FKBPs suggests their likely role in protein-protein interactions (Kurek et al., 2002). Two of the homeologous groups of FKBPs, TaFKBP47-1-5A/TaFKBP47-2-5B/TaFKBP47-3-5D, and TaFKBP48-1-2A/TaFKBP53-1-2D/TaFKBP53-2-2B also contain an NPL-domain along with the FKBD. It is speculated that as reported for AtFKBP53, which contains an NPL-domain (Li and Luan, 2010), these wheat FKBPs might also be acting as histone chaperones, which however, requires experimental validation. Different types of structural motifs observed in TaFKBPs indicate towards their functional specificities. For instance, the presence of motifs 2 and 15 suggests their likely participation in protein-protein interactions, since other multi-domain FKBPs *viz.*, wFKBP73 and wFKBP77 in wheat (Reddy et al., 1998), AtFKBP62 in *A. thaliana* (Meiri and Breiman, 2009) and human FKBP52 (Pirkl and Buchner, 2001), which contain these sequences, were implicated in interaction with the molecular chaperone HSP90 *via* their TPR domain. Similarly, the existence of the motifs 11 and 12 in the N-terminus NPL-domain of two groups of homeologues (TaFKBP47-1-5A/TaFKBP47-2-5B/TaFKBP47-3-5D, and TaFKBP48-1-2A/TaFKBP53-1-2D/TaFKBP53-2-2B) suggests that these proteins may be acting as histone chaperons, as observed for their orthologous proteins AtFKBP53 in *A. thaliana* (Li and Luan, 2010), and FPR3 and FPR4 in *S. cerevisiae* (Benton et al., 1994; Kuzuhara and Horikoshi, 2004). The motif 10 (cytidylate kinase 2) was observed in several (17) FKBPs implying their likely role in pyrimidine biosynthesis, as reported for the cytidylate kinase protein Cmk (Wojtkiewicz et al., 2020). The Aspartic_Protease (ASP_Protease) motif predicted in a homeolog triplet TaFKBP48-1-2A/TaFKBP53-1-2D/TaFKBP53-2-2B indicate the role of these proteins in biotic and abiotic stress responses in plants, since these proteases are known to regulate the pathogenesis-related proteins (Rodrigo et al., 1989; Rodrigo et al., 1991), and nitrogen remobilization under water stress conditions (de Carvalho et al., 2001). The homeologues TaFKBP47-1-5A/TaFKBP47-2-5B/TaFKBP47-3-5D, predicted to harbour motif 14 (Domain of unknown function/CDC45 like protein), may be involved in cell cycle regulation, as demonstrated for the AtCDC45 in *A. thaliana* (Stevens et al., 2004).

All wheat parvulins showed the presence of the characteristic rotamase domain, but the multi-domain parvulins TaPar32-1-2D/TaPar32-2-2B/TaPar32-3-2A also contain an additional rhodanese-like domain towards C-terminus, akin to their *A. thaliana* ortholog AtPin3 (He et al., 2004). The different TaPars exhibited homeolog-dependent differences with respect to the different motifs. For instance, the occurrence of motif 12 was observed only in TaPar32-1-2D and TaPar32-3-2A, but not in their third homeolog TaPar32-2-2B. Similarly, motifs 3 (PPIC-type PPIase domain) and 6 (function not known) were observed most commonly (TaPar11-1-3B, TaPar11-2-4A, TaPar12-1-2D, TaPar13-1-2D, TaPar13-2-2A, TaPar13-3-2B), while Motifs 7 and 11 (functions not known) were noted only in few of these proteins (TaPar11-1-3B, TaPar11-2-4A). It is likely that the differential presence of the motifs might signify functional divergence in the roles of different parvulin homeologues.

### Expression analysis of *TaFKBP*, *TaPar* and *TaPTPA* genes

The expression of *TaFKBP, TaPar* and *TaPTPA* genes was analysed in response to heat stress to understand their likely role in thermotolerance of wheat seedlings. These results revealed that the expression of different *TaFKBP* and *TaPar* genes in wheat is modulated differentially by heat stress, which is in accordance with the earlier studies in *O. sativa*, *Z. mays*, *M. domestica*, and *Penicillium* (Nigam et al., 2008; Yu et al., 2012; Dong et al., 2018; Waseem et al., 2018; Singh et al., 2021). For instance, the heat stress-induced increase in *TaFKBP61-2-7D/TaFKBP62-1-7A/TaFKBP62-2-7B* (Group 1) was also observed for their maize ortholog, *ZmFKBP62a* (Yu et al., 2012). Similarly, the increase in expression of *TaFKBP63-1-1D/TaFKBP67-1-1B/TaFKBP69-1-1A* (Group 2) after exposure to lethal heat stress following acclimation (37 °C-50 °C-4.5 h) is consistent with the temperature-induced increase in expression of *ZmFKBP72* in maize (Yu et al., 2012). Apart from the heat-upregulated members, the *TaFKBP* and *TaPar* families in wheat also possess genes that are downregulated under thermal stress, indicating functional divergence in their roles, which might be contributing to maintenance of homeostasis under stress conditions. The identification of commonly found heat shock elements in the promoter regions of heat-stress inducible genes, such as *TaFKBP20-1-6A* (Group 4) and *TaFKBP21-1-2D*/*TaFKBP27-1-2A* (Group 3), suggests their putative role in stress response, that, however, needs to be explored further by overexpression and knockdown approaches. Of the different *FKBP* genes analyzed, *TaFKBP61-2-7D/TaFKBP62-1-7A/TaFKBP62-2-7B* (Group 1) showed highest increase in transcript levels during acclimation (27.7-fold) and lethal heat stress following acclimation (40.8-fold). These proteins are approximately 98.03% identical with the wFKBP73, that in addition to its PPIase activity was also shown to have chaperonic function (**Supplementary Figure S2A**; Kurek et al., 2002). Therefore, it is likely that as observed for the wheat cyclophilin *TaCYPA-1* (Kaur et al., 2016), the enhanced levels of *TaFKBP61-2-7D/TaFKBP62-1-7A/TaFKBP62-2-7B* under heat stress might be providing protection against heat-induced damage by preventing protein aggregation and mediating protein folding through their chaperone/PPIase activity. As observed for *TaFKBPs*, expression of *TaPars* was also affected differentially by heat stress, prompting us to speculate that besides developmental regulation, these proteins might have an important role in stress response of plants. Though expression of none of the three *TaPTPA* homeologues was detected in the present study, role of these genes can’t be ruled out and, hence, requires further analysis.

### Protein-Protein interaction network

The PPI networks of TaFKPBs, TaPars and TaPTPAs demonstrated that these proteins interact among themselves as well as with other proteins, suggesting their involvement in diverse biological functions (**Figure 12**). The PPI network of FKBPs showed that the TaFKBP65-1-2B and TaFKBP72-1-2D might be playing essential roles in response to abiotic stress (**Figure 12A**), since both of them share the network cluster with heat shock protein 90-1 (HSP90.1) and heat shock transcription factor A-2 (HSFA2), indicating their likely role in regulation of thermotolerance in plants, as reported for ROF1 in *A. thaliana* (AtFKBP62) (Meiri and Breiman, 2009). The role of TaFKBP65-1-2B and TaFKBP72-1-2D in thermotolerance is also substantiated by their enhanced expression in heat stress, as revealed by heatmap analysis (**Figure 11A**). Similarly, the putative interaction of TaFKBP42-1-5B with ABC transporter B family members ABCB1 and ABCB19 is consistent with the previous study reporting interaction of *Arabidopsis* ortholog AtFKBP42 (TWD1) with vacuolar ABC transporters, which is important for auxin transport (Geisler et al., 2003; Geisler et al., 2004).

Computational analysis also predicted the interaction between TaPar13-3-2B and serine/threonine- protein phosphatase 2α (PP2A), implying its role in regulation of cell cycle in plants (Yeh and Means, 2007; **Figure 12B**). The putative interaction between TaPTPA42-2-7A and HEAT (huntingtin-elongation-A subunit-TOR) repeat-containing protein (AT5G16210) indicates its association with the PP2A scaffolding subunit that comprises of 15 tandem repeats of HEAT motif (Walter et al., 1989; Hemmings et al., 1990; **Figure 12C**). Though the PPI network analyses indicate the role of TaFKBPs, TaPars and TaPTPA in different cellular functions, understanding the mechanisms underlying these processes requires detailed analysis of *in vivo* interactions by BiFC/FRET approaches.

## Conclusion

To conclude, the genes encoding different FKBP, parvulin and PTPA proteins were identified for the first time in wheat genome. Further, characterization of gene structure, domain architecture, motif analysis, cis*-*regulatory elements and evolutionary analysis was also carried out. The results of this study suggest functional divergence of these proteins, that needs to be elucidated by experimental approaches. The expression profiling of these genes at the seedling stage revealed that the *TaFKBP* and *TaPar* genes in wheat are differentially regulated in response to heat stress, implying their role in stress response. The findings of this study will provide the basis for further functional characterization of these gene families in wheat, that may lead to their application in improvement of crop plants through breeding and biotechnological approaches.

## Data availability statement

The datasets presented in this study can be found in online repositories. The names of the repository/repositories and accession number(s) can be found in the article/**Supplementary Material**.

## Author contributions

PS: conceived idea, designed the experiments, reviewing and editing of the manuscript; HS: designed the bioinformatic study, participated in writing of manuscript, data analysis; AS: conducted bioinformatic analysis and performed qRT-PCR analysis of genes, writing of manuscript, data curation and analysis; KK: helped in qRT-PCR analysis of genes, data analysis; AK: contributed in writing of manuscript. All authors contributed to the article and approved the submitted version.
