# Supplementary material for: Genome-wide characterization of FK506-binding proteins, parvulins and phospho-tyrosyl phosphatase activators in wheat and their regulation by heat stress
Source: Front Plant Sci. 2022 Dec 15;13:1053524. doi: 10.3389/fpls.2022.1053524 (PMC9797600; doi:10.3389/fpls.2022.1053524)
Supplement: Supplementary file 1 [file DataSheet_1.pdf]

Table S1. The different FK506-binding proteins (FKBPs), parvulins and phospho-tyrosyl phosphatase activators (PTPAs) encoded by wheat genome (MW: molecular weight; pI: isoelectric point; Loc: subcellular localization; N.D: not determined; C: cytoplasm; Ch: chloroplast; N: nucleus; E.R: endoplasmic reticulum; * exact molecular weight and pI not determined).

**TaFKBPs**										
**S. No**	**Protein**	**Amino acids**	**MW (kDa)**	**pI**	**Loc**	**Domain type**	**Orthologs**			
							O. sativa	***Arabidopsis***	***P. persica***	***M. domestica***
1	TaFKBP7-1-2B	68	7.596	10.13	Ch	FKBP	OsFKBP19	FKBP19	FKBP19	
2	TaFKBP15-1-3D	148	15.782	8.69	C	FKBP	OsFKBP15-2	FKBP15-2	FKBP15-1	MdFKBP15-1, MdFKBP15-2
3	TaFKBP16-1-5B	152	16.021	5.94	C	FKBP	OsFKBP15-1	FKBP15-1	FKBP15-1	MdFKBP15-1, MdFKBP15-2
4	TaFKBP16-2-5D	152	16.061	5.68	C	FKBP	OsFKBP15-1	FKBP15-1	FKBP15-1	MdFKBP15-1, MdFKBP15-2
5	TaFKBP16-3-5A	152	16.137	5.68	C	FKBP	OsFKBP15-1	FKBP15-1	FKBP15-1	MdFKBP15-1, MdFKBP15-2
6	TaFKBP16-4-3D	156	16.514	8.23	Ch	FKBP	OsFKBP15-2	FKBP15-2	FKBP15-1	MdFKBP15-1, MdFKBP15-2
7	TaFKBP16-5-3A	158	16.689	9.08	C	FKBP	OsFKBP15-2	FKBP15-2	FKBP15-1	MdFKBP15-1, MdFKBP15-2
8	TaFKBP16-6-3B	158	16.714	9.28	C	FKBP	OsFKBP15-2	FKBP15-2	FKBP15-1	MdFKBP15-1, MdFKBP15-2
9	TaFKBP16-7-2B	151	16.83	5.49	Ch	FKBP	OsFKBP19	FKBP19	FKBP19	
10	TaFKBP17-1-3A	156	17.526	5.67	Ch	FKBP	OsFKBP15-2	FKBP15-2	FKBP15-1	MdFKBP15-1, MdFKBP15-2
11	TaFKBP17-2-2A	165	17.689	4.86	C	FKBP				
12	TaFKBP18-1-6A	166	18.046	4.32	C	FKBP				
13	TaFKBP19-1-6A	138	19.525	8.15	Ch	FKBP	OsFKBP18	FKBP18	FKBP18	
14	TaFKBP20-1-6A	193	20.078	8.84	C	FKBP	OsFKBP12	FKBP12	FKBP12	MdFKBP12
15	TaFKBP20-2-6D	195	20.443	9.56	C	FKBP	OsFKBP12	FKBP12	FKBP12	MdFKBP12
16	TaFKBP20-3-6B	195	20.486	9.56	C	FKBP	OsFKBP12	FKBP12	FKBP12	MdFKBP12
17	TaFKBP20-4-7B	203	20.857	7.99	Ch	FKBP	OsFKBP13	FKBP13	FKBP13	MdFKBP13
18	TaFKBP20-5-7D	203	20.901	8.32	Ch	FKBP	OsFKBP13	FKBP13	FKBP13	MdFKBP13
19	TaFKBP20-6-2D	187	20.909	8.83	Ch	FKBP				
20	TaFKBP20-7-7A	203	20.927	8.32	Ch	FKBP	OsFKBP13	FKBP13	FKBP13	MdFKBP13
21	TaFKBP21-1-2D	192	21.026	8.33	Ch	FKBP	OsFKBP20-2	FKBP20-2	FKBP20-2	MdFKBP20-2a,MdFKBP20-2b,MdFKBP20-2c
22	TaFKBP21-2-1D	202	21.739	6.99	C	FKBP	OsFKBP20-1a	FKBP 20-1		MdFKBP20-1a,MdFKBP20-1b
23	TaFKBP21-3-1B	202	21.783	6.99	C	FKBP	OsFKBP20-1a	FKBP 20-1		MdFKBP20-1a,MdFKBP20-1b
24	TaFKBP21-4-6D	207	21.842	10.01	Ch	FKBP	OsFKBP17-1	FKBP17-1	FKBP17-1	MdFKBP17-1
25	TaFKBP21-5-6A	207	21.855	10.01	C	FKBP	OsFKBP17-1	FKBP17-1	FKBP17-1	MdFKBP17-1
26	TaFKBP21-6-6B	207	21.885	10.17	Ch	FKBP	OsFKBP17-1	FKBP17-1	FKBP17-1	MdFKBP17-1
27	TaFKBP21-7-7B	220	21.938	7.09	Ch	FKBP	OsFKBP16-3	FKBP16-3	FKBP16-3	MdFKBP16-3
28	TaFKBP22-1-2B	198	22.133	9.05	Ch	FKBP	OsFKBP20-2	FKBP20-2	FKBP20-2	MdFKBP20-2a, MdFKBP20-2b, MdFKBP20-2c
29	TaFKBP22-2-6A	222	22.458	9.13	Ch	FKBP	OsFKBP16-2	FKBP16-2	FKBP16-2	MdFKBP16-2
30	TaFKBP22-3-6B	221	22.465	9.13	Ch	FKBP	OsFKBP16-2	FKBP16-2	FKBP16-2	MdFKBP16-2
31	TaFKBP22-4-6D	222	22.49	9.13	Ch	FKBP	OsFKBP16-2	FKBP16-2	FKBP16-2	MdFKBP16-2
32	TaFKBP22-5-6D	208	22.66	9.39	Ch	FKBP	OsFKBP16-1	FKBP16-1	FKBP16-1	
33	TaFKBP23-1-6B	212	23.08	8.91	Ch	FKBP	OsFKBP16-1	FKBP16-1	FKBP16-1	

Continued…

34	TaFKBP23-2-5D	222	23.24	10.68	Ch	FKBP	OsFKBP16-4	FKBP16-4	FKBP16-4. FKBP 18	MdFKBP18
35	TaFKBP23-3-6A	212	23.242	8.91	Ch	FKBP	OsFKBP16-1	FKBP16-1	FKBP16-1	
36	TaFKBP23-4-5A	223	23.293	10.49	Ch	FKBP	OsFKBP16-4	FKBP16-4	FKBP16-4. FKBP 18	MdFKBP18
37	TaFKBP23-5-5B	223	23.424	10.58	Ch	FKBP	OsFKBP16-4	FKBP16-4	FKBP16-4. FKBP 18	MdFKBP18
38	TaFKBP23-6-U	219	23.588	9.95	C	FKBP	OsFKBP18	FKBP18	FKBP18	
39	TaFKBP23-7-7D	235	23.761	6.17	Ch	FKBP	OsFKBP16-3	FKBP16-3	FKBP16-3	MdFKBP16-3
40	TaFKBP24-1-6B	225	24.015	9.95	Ch	FKBP	OsFKBP18	FKBP18	FKBP18	
41	TaFKBP24-2-2B	220	24.282	9.37	C	FKBP	OsFKBP19	FKBP19	FKBP19	
42	TaFKBP24-3-7A	247	24.846	7.13	Ch	FKBP	OsFKBP16-3	FKBP16-3	FKBP16-3	MdFKBP16-3
43	TaFKBP26-1-4D	250	26.249	5.92	Ch	FKBP	OsFKBP17-2	FKBP17-2	FKBP17-2	
44	TaFKBP26-2-4A	251	26.395	5.14	Ch	FKBP	OsFKBP17-2	FKBP17-2	FKBP17-2	
45	TaFKBP26-3-4B	255	26.758	5.51	Ch	FKBP	OsFKBP17-2	FKBP17-2	FKBP17-2	
46	TaFKBP27-1-2A	244	27.136	9.69	Ch	FKBP	OsFKBP20-2	FKBP20-2	FKBP20-2	MdFKBP20-2a, MdFKBP20-2b, MdFKBP20-2c
47	TaFKBP27-2-1A	258	27.835	9.6	C	FKBP	OsFKBP20-1a	FKBP 20-1		MdFKBP20-1a, MdFKBP20-1b
48	TaFKBP29-1-2A	263	29.041	10.32	Ch	FKBP	OsFKBP19	FKBP19	FKBP19	
49	TaFKBP29-2-2D	264	29.091	10.25	Ch	FKBP	OsFKBP19	FKBP19	FKBP19	
50	TaFKBP36-1-5A	315	36.276	9.71	E.R	FKBP, TPR	OsFKBP42b	FKBP42		
51	TaFKBP41-1-5D	370	41.927	5.95	C	FKBP, TPR	OsFKBP42a	FKBP42	FKBP42a, FKBP42b	MdFKBP42a, MdFKBP42b, MdFKBP42c, MdFKBP42d, MdFKBP42e, MdFKBPf
52	TaFKBP42-1-5B	370	42.102	6.32	C	FKBP, TPR	OsFKBP42a	FKBP42	FKBP42a, FKBP42b	MdFKBP42a, MdFKBP42b, MdFKBP42c, MdFKBP42d, MdFKBP42e, MdFKBPf
53	TaFKBP47-1-5A	432	47.59	5.25	N	FKBP, NPL	OsFKBP53	FKBP53	FKBP53a	
54	TaFKBP47-2-5B	433	47.753	5.05	N	FKBP, NPL	OsFKBP53	FKBP53	FKBP53a	
55	TaFKBP47-3-5D	433	47.802	5.37	C	FKBP, NPL	OsFKBP53	FKBP53	FKBP53a	
56	TaFKBP48-1-2A	444	48.832	5.95	N	FKBP, NPL	OsFKBP58			
57	TaFKBP53-1-2D	487	53.441	5.34	C	FKBP, NPL	OsFKBP58			
58	TaFKBP53-2-2B	487	53.466	5.56	C	FKBP, NPL	OsFKBP58			
59	TaFKBP61-1-2D	550	61.355	6.38	C	3 FKBPs, TPR	OsFKBP62b			
60	TaFKBP61-2-7D	559	61.978	5	C	3 FKBPs, TPR	OsFKBP62 a			
61	TaFKBP62-1-7A	559	62.01	5	C	3 FKBPs, TPR	OsFKBP62 a			
62	TaFKBP62-2-7B	559	62.02	5.1	C	3 FKBPs, TPR	OsFKBP62 a			
63	TaFKBP63-1-1D	575	63.973	5.28	N	3 FKBPs, TPR	OsFKBP72			
64	TaFKBP64-1-2A	582	64.895	4.96	C	3 FKBPs, TPR	OsFKBP62b			
65	TaFKBP65-1-2B	587	65.604	4.88	C	3 FKBPs, TPR	OsFKBP62b			
66	TaFKBP67-1-1B	607	67.968	5.5	N	3 FKBPs, TPR	OsFKBP72			
67	TaFKBP69-1-1A	624	69.603	5.64	N	3 FKBPs, TPR	OsFKBP72			
68	TaFKBP71-1-2B	638	71.304	5.29	C	3 FKBPs, TPR	OsFKBP62c			
69	TaFKBP71-2-2A	645	71.806	5.05	C	3 FKBPs, TPR	OsFKBP62c			
70	TaFKBP72-1-2D	649	72.074	4.98	C	3 FKBPs, TPR	OsFKBP62c			

Continued…

71	TaFKBP77-1-2B	629	77.105	5.9	C	FKBP				

TaPars

S. No	Protein	Amino acids	MW (kDa)	pI	Loc	Domain type	Orthologs			
							Arabidopsis	***E. coli***	**Humans**	***S. cerevisiae***
1	TaPar11-1-3B	99	11.0628	6.46	C	Rotamase	At2g18040 (AtPin1)			
2	TaPar11-2-4A	99	11.1669	6.15	N	Rotamase	At2g18040 (AtPin1)			
3	TaPar12-1-2D	113	12.1752	7.35	N	Rotamase	At2g18040 (AtPin1)		Pin1	Ptf1/ScESS1
4	TaPar13-1-2D	121	13.0052	9.41	N	Rotamase	At2g18040 (AtPin1)		Pin1	Ptf1/ScESS1
5	TaPar13-2-2A	121	13.0512	8.89	N	Rotamase	At2g18040 (AtPin1)		Pin1	Ptf1/ScESS1
6	TaPar13-3-2B	123	13.2164	9.92	N	Rotamase	At2g18040 (AtPin1)		Pin1	Ptf1/ScESS1
7	TaPar15-1-5D	148	15.4212	9.73	N	Rotamase	AT1G26550 (AtPin2)		Pin1	Ptf1/ScESS1
8	TaPar15-2-5B	148	15.435	9.73	N	Rotamase	AT1G26550 (AtPin2)	EcPar10	Pin1	Ptf1/ScESS1
9	TaPar15-3-5A	148	15.465	9.73	N	Rotamase	AT1G26550 (AtPin2)	EcPar10	Pin1	Ptf1/ScESS1
10	TaPar32-1-2D	299	32.514	7.05	C	Rotamase, Rhodanese	At5g19370 (AtPin3)	EcPar10	Par14	
11	TaPar32-2-2B	299	32.5609	7.08	C	Rotamase, Rhodanese	At5g19370 (AtPin3)	EcPar10	Par14	
12	TaPar32-3-2A	301	32.704	7.08	C	Rotamase, Rhodanese	At5g19370 (AtPin3)	EcPar10	Par14	

TaPTPAs

S. No	Protein	Amino acids	MW (kDa)	pI	Loc	Domain type	Orthologs
1	TaPTPA41-1-7D*	393	>41.058	-	C	PTPA	***D. melanogaster*** PTPA (Q24466),<br>***S. cerevisiae*** YPTPA1 (P40454.1); YPTPA2 (Q12461),<br>***S. pombe*** (Q9P7H4)
2	TaPTPA42-1-7B	393	42.466	6.91	C	PTPA	
3	TaPTPA42-2-7A	394	42.576	7.1	C	PTPA	

Table S2. Nomenclature and transcript IDs of *TaFKBPs, TaPars and TaPTPAs*.

S. No	Nomenclature	Transcript IDs
FKBPs		
1	*TaFKBP7-1-2B*	TRAESCS2B02G071600LC.1
2	*TaFKBP15-1-3D*	TRAESCS3D02G502100LC.1
3	*TaFKBP16-1-5B*	TRAESCS5B02G278800.1
4	*TaFKBP16-2-5D*	TRAESCS5D02G286500.1
5	*TaFKBP16-3-5A*	TRAESCS5A02G279500.1
6	*TaFKBP16-4-3D*	TRAESCS3D02G417400.1
7	*TaFKBP16-5-3A*	TRAESCS3A02G422000.1
8	*TaFKBP16-6-3B*	TRAESCS3B02G457500.1
9	*TaFKBP16-7-2B*	TRAESCS2B02G067200.1
10	*TaFKBP17-1-3A*	TRAESCS3A02G421300.1
11	*TaFKBP17-2-2A*	TRAESCS2A02G274800LC.1
12	*TaFKBP18-1-6A*	TRAESCS6A02G566500LC.1
13	*TaFKBP19-1-6A*	TRAESCS6A02G050000.1
14	*TaFKBP20-1-6A*	TRAESCS6A02G314100.1

15	*TaFKBP20-2-6D*	TRAESCS6D02G293400.1
16	*TaFKBP20-3-6B*	TRAESCS6B02G344100.1
17	*TaFKBP20-4-7B*	TRAESCS7B02G388800.1
18	*TaFKBP20-5-7D*	TRAESCS7D02G472600.1
19	*TaFKBP20-6-2D*	TRAESCS2D02G053100.1
20	*TaFKBP20-7-7A*	TRAESCS7A02G485300.1
21	*TaFKBP21-1-2D*	TRAESCS2D02G239700.1
22	*TaFKBP21-2-1D*	TRAESCS1D02G282500.1
23	*TaFKBP21-3-1B*	TRAESCS1B02G292400.1
24	*TaFKBP21-4-6D*	TRAESCS6D02G132400.2
25	*TaFKBP21-5-6A*	TRAESCS6A02G143200.1
26	*TaFKBP21-6-6B*	TRAESCS6B02G171400.1
27	*TaFKBP21-7-7B*	TRAESCS7B02G166000.1
28	*TaFKBP22-1-2B*	TRAESCS2B02G353500LC.1
29	*TaFKBP22-2-6A*	TRAESCS6A02G302500.1
30	*TaFKBP22-3-6B*	TRAESCS6B02G331700.1
31	*TaFKBP22-4-6D*	TRAESCS6D02G282000.1
32	*TaFKBP22-5-6D*	TRAESCS6D02G150200.1
33	*TaFKBP23-1-6B*	TRAESCS6B02G189300.1
34	*TaFKBP23-2-5D*	TRAESCS5D02G272100.1
35	*TaFKBP23-3-6A*	TRAESCS6A02G162000.1
36	*TaFKBP23-4-5A*	TRAESCS5A02G264500.1
37	*TaFKBP23-5-5B*	TRAESCS5B02G263800.1
38	*TaFKBP23-6-U*	TRAESCSU02G108900.1
39	*TaFKBP23-7-7D*	TRAESCS7D02G268300.1
40	*TaFKBP24-1-6B*	TRAESCS6B02G066300.1
41	*TaFKBP24-2-2B*	TRAESCS2B02G067100.1
42	*TaFKBP24-3-7A*	TRAESCS7A02G266500.1
43	*TaFKBP26-1-4D*	TRAESCS4D02G041200.1
44	*TaFKBP26-2-4A*	TRAESCS4A02G270400.1
45	*TaFKBP26-3-4B*	TRAESCS4B02G043700.1
46	*TaFKBP27-1-2A*	TRAESCS2A02G226800.1
47	*TaFKBP27-2-1A*	TRAESCS1A02G427400LC.1
48	*TaFKBP29-1-2A*	TRAESCS2A02G053200.1
49	*TaFKBP29-2-2D*	TRAESCS2D02G053400.1
50	*TaFKBP36-1-5A*	TRAESCS5A02G134500.1
51	*TaFKBP41-1-5D*	TRAESCS5D02G139000.1
52	*TaFKBP42-1-5B*	TRAESCS5B02G131200.1
53	*TaFKBP47-1-5A*	TRAESCS5A02G158900.1
54	*TaFKBP47-2-5B*	TRAESCS5B02G156700.1

55	*TaFKBP47-3-5D*	TRAESCS5D02G164000.1
56	*TaFKBP48-1-2A*	TRAESCS2A02G314800.1
57	*TaFKBP53-1-2D*	TRAESCS2D02G313000.1
58	*TaFKBP53-2-2B*	TRAESCS2B02G333300.1
59	*TaFKBP61-1-2D*	TRAESCS2D02G276000.1
60	*TaFKBP61-2-7D*	TRAESCS7D02G257300.1
61	*TaFKBP62-1-7A*	TRAESCS7A02G257100.1
62	*TaFKBP62-2-7B*	TRAESCS7B02G153100.1
63	*TaFKBP63-1-1D*	TRAESCS1D02G192100.1
64	*TaFKBP64-1-2A*	TRAESCS2A02G277100.1
65	*TaFKBP65-1-2B*	TRAESCS2B02G294500.1
66	*TaFKBP67-1-1B*	TRAESCS1B02G192700.1
67	*TaFKBP69-1-1A*	TRAESCS1A02G184600.1
68	*TaFKBP71-1-2B*	TRAESCS2B02G063900.1
69	*TaFKBP71-2-2A*	TRAESCS2A02G050600.1
70	*TaFKBP72-1-2D*	TRAESCS2D02G050300.1
71	*TaFKBP77-1-2B*	TRAESCS2B02G490400LC.3
Parvulins		
1	*TaPar11-1-3B*	TRAESCS3B02G116600.1
2	*TaPar11-2-4A*	TRAESCS4A02G388100.1
3	*TaPar12-1-2D*	TRAESCS2D02G491300.1
4	*TaPar13-1-2D*	TRAESCS2D02G009700.1
5	*TaPar13-2-2A*	TRAESCS2A02G007900.1
6	*TaPar13-3-2B*	TRAESCS2B02G012800.1
7	*TaPar15-1-5D*	TRAESCS5D02G238100.1
8	*TaPar15-2-5B*	TRAESCS5B02G228100.1
9	*TaPar15-3-5A*	TRAESCS5A02G229400.1
10	*TaPar32-1-2D*	TRAESCS2D02G096900.1
11	*TaPar32-2-2B*	TRAESCS2B02G113300.1
12	*TaPar32-3-2A*	TRAESCS2A02G097600.1
PTPAs		
1	*TaPTPA41-1-7D**	TRAESCS7D02G179900.1
2	*TaPTPA42-1-7B*	TRAESCS7B02G083800.1
3	*TaPTPA42-2-7A*	TRAESCS7A02G178100.1

Table S3. Nomeclature, transcript IDs, molecular weights and genomic locations of *FKBP*, parvulin and *PTPA* genes of different wheat sub-genome donors. (*Triticum urartu*: A genome; *Aegilops tauschii*: D genome; *Triticum dicoccoides*: AB genome).

S. No	Nomenclature	Ensembl ID	Molecular weight (kDa)	Genomic location

FKBPs				
T. urartu				
1	*TuFKBP12-1*	TRIUR3_12181-P1	12.014	6A:512708296:512710032
2	*TuFKBP15-1*	TRIUR3_04597-P1	15.294	5A:449949879:449954788
3	*TuFKBP15-2*	TRIUR3_16286-P1	15.637	7A:265435576:265436969
4	*TuFKBP16-1*	TRIUR3_19464-P1	16.137	5A:468377786:468375757
5	*TuFKBP17-1*	TRIUR3_05164-P1	17.07	7A:658607155:658608367
6	*TuFKBP20-1*	TRIUR3_28895-P1	20.909	7A:658606703:658607550
7	*TuFKBP21-1*	TRIUR3_20703-P1	21.422	3A:651506085:651508806
8	*TuFKBP22-1*	TRIUR3_07834-P1	22.818	7A:625381652:625384798
9	*TuFKBP32-1*	TRIUR3_14867-P1	32.52	2A:22222915:22226655
10	*TuFKBP43-1*	TRIUR3_34328-P1	43.972	5A:323592903:323596201
11	*TuFKBP64-1*	TRIUR3_28777	64.982	3A:22681040:22683900
12	*TuFKBP69-1*	TRIUR3_20435-P1	69.692	2A:520299609:520304267
13	*TuFKBP72-1*	TRIUR3_23001	72.67	4A:52329053:52333737
14	*TuFKBP96-1*	TRIUR3_33562	96.216	1A:334911285:334934374
T. dicoccoides				
1	*TdFKBP15-1-6A*	TRIDC6AG045670.1	15.204	6A:535902360:535903740
2	*TdFKBP16-1-5A*	TRIDC5AG041980.2	16.137	5A:482898715:482901068
3	*TdFKBP16-2-4B*	TRIDC4BG059810.2	16.736	4B:652402353:652405423
4	*TdFKBP16-3-6B*	TRIDC6BG008570.2	16.877	6B:43317032:43318381
5	*TdFKBP16-4-3B*	TRIDC3BG067640.1	16.998	3B:714320577:714323532
6	*TdFKBP17-1-5B*	TRIDC5BG043690.3	17.198	5B:455335799:455340025
7	*TdFKBP17-2-4A*	TRIDC4AG042120.1	17.274	4A:575010990:575012286
8	*TdFKBP17-3-6A*	TRIDC6AG006100.1	17.545	6A:25292626:25293953
9	*TdFKBP18-1-5B*	TRIDC5BG045950.2	18.1126	5B:470700071:470702344
10	*TdFKBP18-2-7B*	TRIDC7BG061560.2	18.295	7B:662129065:662130038
11	*TdFKBP20-1-6A*	TRIDC6AG047230.1	20.078	6A:550580147:550586195
12	*TdFKBP21-1-6B*	TRIDC6BG055010.1	21.101	6B:592392264:592395599
13	*TdFKBP21-2-6B*	TRIDC6BG025600.1	21.441	6B:188543493:188552164
14	*TdFKBP22-1-6A*	TRIDC6AG019570.2	22.341	6A:118990098:118992592
15	*TdFKBP23-1-6B*	TRIDC6BG053280.2	23.532	6B:567287204:567289117
16	*TdFKBP23-2-1B*	TRIDC1BG048100.4	23.577	1B:515736419:515740542
17	*TdFKBP24-1-6B*	TRIDC6BG028420.2	24.728	6B:221225154:221227598
18	*TdFKBP25-1-2A*	TRIDC2AG031120.4	25.324	2A:268170992:268177757
19	*TdFKBP25-2-7A*	TRIDC7AG067670.2	25.944	7A:671453651:671454641
20	*TdFKBP26-1-6A*	TRIDC6AG022950.7	26.007	6A:155736234:155740197
21	*TdFKBP26-2-7B*	TRIDC7BG026200.1	26.669	7B:244927932:244931604
22	*TdFKBP26-3-7A*	TRIDC7AG034890.4	26.83	7A:266858287:266860167
23	*TdFKBP27-1-2A*	TRIDC2AG005770.2	27.757	2A:25070595:25075983
24	*TdFKBP29-1-2B*	TRIDC2BG006520.1	29.139	2B:31096186:31141837
25	*TdFKBP29-2-2B*	TRIDC2BG035180.1	29.362	2B:295061120:295071173
26	*TdFKBP30-1-1A*	TRIDC1AG042210.3	30.441	1A:482863129:482915896

27	*TdFKBP30-2-4B*	TRIDC4BG006840.1	30.573	4B:30229926:30230974
28	*TdFKBP42-1-5A*	TRIDC5AG022730.1	42.071	5A:298875525:298880877
29	*TdFKBP42-2-5B*	TRIDC5BG023220.2	42.102	5B:251706729:251710963
30	*TdFKBP47-1-5B*	TRIDC5BG027500.1	47.737	5B:297524481:297528667
31	*TdFKBP47-2-5A*	TRIDC5AG026300.3	47.99	5A:336422859:336426618
32	*TdFKBP51-1-2A*	TRIDC2AG045790.1	51.754	2A:535450643:535454150
33	*TdFKBP52-1-1A*	TRIDC1AG027960.3	52.057	1A:336672369:336693548
34	*TdFKBP53-1-2B*	TRIDC2BG048690.6	53.48	2B:475137042:475140687
35	*TdFKBP63-1-7B*	TRIDC7BG024260.1	63.739	7B:219750891:219756338
36	*TdFKBP65-1-2B*	TRIDC2BG043040.2	65.502	2B:412620263:412623662
37	*TdFKBP65-2-7A*	TRIDC7AG033300.4	65.792	7A:244568999:244574766
38	*TdFKBP67-1-2A*	TRIDC2AG039800.6	67.019	2A:452372887:452376276
39	*TdFKBP68-1-1B*	TRIDC1BG032200.4	68.094	1B:351962506:351984892
40	*TdFKBP71-1-2B*	TRIDC2BG005970.2	71.304	2B:26942882:26948242
A. tauschii				
1	*AetFKBP16-1-5D*	AET5Gv20651200	16.0612	5D:393835593:393838028
2	*AetFKBP16-2-3D*	AET3Gv20939000	16.456	3D:537760492:537763565
3	*AetFKBP19-1-1D*	AET1Gv20678400	19.955	1D:385824419:385831780
4	*AetFKBP20-1-6D*	AET6Gv20774300	20.443	6D:428004306:428032288
5	*AetFKBP20-2-7D*	AET7Gv21186300.3	20.901	7D:591177701:591179050
6	*AetFKBP21-1-6D*	AET6Gv20369100	21.842	6D:123416625:123422087
7	*AetFKBP22-1-6D*	AET6Gv20750800	22.49	6D:413258225:413259780
8	*AetFKBP22-2-6D*	AET6Gv20407800	22.66	6D:148079538:148082212
9	*AetFKBP23-1-5D*	AET5Gv20621000	23.24	5D:382180892:382191157
10	*AetFKBP26-1-4D*	AET4Gv20081900	26.249	4D:21176217:21179152
11	*AetFKBP27-1-2D*	AET2Gv20500200	27.507	2D:227655708:227659129
12	*AetFKBP27-2-7D*	AET7Gv20655200.1	27.518	7D:254023231:254025728
13	*AetFKBP29-1-2D*	AET2Gv20091000.3	29.133	2D:21477515:21482647
14	*AetFKBP41-1-5D*	AET5Gv20353500	41.927	5D:227782793:227787077
15	*AetFKBP50-1-5D*	AET5Gv20402300	50.916	5D:262242790:262246746
16	*AetFKBP53-1-2D*	AET2Gv20714700.1	53.46	2D:401635256:401638948
17	*AetFKBP62-1-7D*	AET7Gv20630200.12	62.9	7D:234530195:234536089
18	*AetFKBP65-1-2D*	AET2Gv20626600.3	65.55	2D:344353974:344357364
19	*AetFKBP69-1-1D*	AET1Gv20488600.4	69.649	1D:273351097:273367209
20	*AetFKBP72-1-2D*	AET2Gv20084600.3	72.074	2D:19389021:19393800
Parvulins				
T. urartu				
1	*TuPar6-1*	TRIUR3_12387-P1	6.716	2A:5781301:5782585
2	*TuPar18-1*	TRIUR3_00979-P1	18.133	5A:422895627:422897801
3	*TuPar27-1*	TRIUR3_19936-P1	27.87	2A:45693028:45696150
T. dicoccoides				
1	*TdPar12-1-3B*	TRIDC3BG016320.1	12.294	3B:92232767:92233356
2	*TdPar15-1-5A*	TRIDC5AG036500.1	15.404	5A:443015172:443017489

3	*TdPar15-2-5B*	TRIDC5BG038500.1	15.435	5B:415728942:415737130
4	*TdPar16-1-2B*	TRIDC2BG000800.2	16.163	2B:3308408:3310506
5	*TdPar17-1-2A*	TRIDC2AG000780.3	17.11	2A:2880521:2882404
6	*TdPar23-1-2B*	TRIDC2BG075010.1	23.571	2B:709139623:709142465
7	*TdPar23-2-2A*	TRIDC2AG069340.1	23.946	2A:717022050:717024654
8	*TdPar26-1-2A*	TRIDC2AG011290.1	26.696	2A:54237209:54240566
9	*TdPar28-1-2B*	TRIDC2BG014100.1	28.5	2B:85598812:85602711
A. tauschii				
1	*AetPar11-1-5D*	AET5Gv20555700.1	11.906	5D:353217130:353219504
2	*AetPar13-1-2D*	AET2Gv20017500.2	13.019	2D:4199237:4201483
3	*AetPar14-1-2D*	AET2Gv20195400.1	14.83	2D:50075093:50078367
4	*AetPar21-1-2D*	AET2Gv21082500.1	21.249	2D:588262994:588265572
5	*AetPar22-1-2D*	AET2Gv21082300.1	22.203	2D:588250007:588252637
PTPAs				
T. urartu				
1	*TuPTPA22-1*	TRIUR3_14294-P1	22.015	-
A. tauschii				
1	*AetPTPA46-1-7D*	AET7Gv20461700.1	46.22	7D:134899868:134905144

Table S4: Table representing pairs of tandemly duplicated genes.

S. No	Gene pairs	
	A. tauschii	
1	*AetFKBP27-2-7D*	*AetFKBP20-2-7D*
2	*AetFKBP21-1-6D*	*AetFKBP22-2-6D*
3	*AetFKBP29-1-2D*	*AetFKBP27-1-2D*
	T. dicoccoides	
1	*TdFKBP21-2-6B*	*TdFKBP24-1-6B*
2	*TdFKBP27-1-2A*	*TdFKBP25-1-2A*
	T. urartu	
1	*TuFKBP20-1*	*TuFKBP17-1*
2	*TuFKBP64-1*	*TuFKBP21-1*

	T. aestivum	
1	*TaFKBP15-1-3D*	*TaFKBP16-4-3D*
2	*TaFKBP23-5-5B*	*TaFKBP16-1-5B*
3	*TaFKBP65-1-2B*	*TaFKBP77-1-2B*
4	*TaFKBP61-1-2D*	*TaFKBP53-1-2D*
5	*TaFKBP64-1-2A*	*TaFKBP48-1-2A*
6	*TaFKBP69-1-1A*	*TaFKBP27-2-1A*
7	*TaFKBP29-1-2A*	*TaFKBP27-1-2A*
8	*TaFKBP62-1-7A*	*TaFKBP24-3-7A*
9	*TaFKBP24-2-2B*	*TaFKBP7-1-2B*
10	*TaFKBP23-2-5D*	*TaFKBP16-2-5D*
11	*TaFKBP23-4-5A*	*TaFKBP16-3-5A*
12	*TaFKBP61-2-7D*	*TaFKBP23-7-7D*
13	*TaFKBP47-2-5B*	*TaFKBP23-5-5B*
14	*TaFKBP47-3-5D*	*TaFKBP23-2-5D*
15	*TaFKBP22-1-2B*	*TaFKBP65-1-2B*
16	*TaFKBP62-2-7B*	*TaFKBP21-7-7B*
17	*TaFKBP21-6-6B*	*TaFKBP23-1-6B*
18	*TaFKBP21-5-6A*	*TaFKBP23-3-6A*
19	*TaFKBP21-4-6D*	*TaFKBP22-5-6D*
20	*TaFKBP67-1-1B*	*TaFKBP21-3-1B*
21	*TaFKBP24-3-7A*	*TaFKBP20-7-7A*
22	*TaFKBP20-6-2D*	*TaFKBP29-2-2D*

23	*TaFKBP23-7-7D*	*TaFKBP20-5-7D*
24	*TaFKBP21-7-7B*	*TaFKBP20-4-7B*
25	*TaFKBP17-2-2A*	*TaFKBP64-1-2A*
26	*TaFKBP17-1-3A*	*TaFKBP16-5-3A*
27	*TaFKBP16-7-2B*	*TaFKBP22-1-2B*
28	*TaFKBP7-1-2B*	*TaFKBP16-7-2B*
29	*TaFKBP63-1-1D*	*TaFKBP21-2-1D*

Table S5. Collinear blocks of genes present in (a) *A. tauschii* and *T. aestivum*; (b) *T. aestivum* and *T. dicoccoides*; (c) *T. aestivum*; (d) *T. dicoccoides.*

S. No	Gene blocks	
**(a)**	***A. tauschii***	***T. aestivum***
1	*AetFKBP72-1-2D*<br>*AetFKBP29-1-2D*<br>*AetFKBP27-1-2D*<br>*AetFKBP53-1-2D*	*TaFKBP71-1-2B*<br>*TaFKBP24-2-2B*<br>*TaFKBP16-7-2B*<br>*TaFKBP53-2-2B*
2	*AetFKBP21-1-6D*<br>*AetFKBP22-2-6D*<br>*AetFKBP22-1-6D*<br>*AetFKBP20-1-6D*	*TaFKBP21-5-6A*<br>*TaFKBP23-3-6A*<br>*TaFKBP22-2-6A*<br>*TaFKBP20-1-6A*
3	*AetFKBP21-1-6D*<br>*AetFKBP22-2-6D*<br>*AetFKBP22-1-6D*<br>*AetFKBP20-1-6D*	*TaFKBP21-6-6B*<br>*TaFKBP23-1-6B*<br>*TaFKBP22-3-6B*<br>*TaFKBP20-3-6B*
4	*AetFKBP21-1-6D*<br>*AetFKBP22-2-6D*<br>*AetFKBP22-1-6D*<br>*AetFKBP20-1-6D*	*TaFKBP21-4-6D*<br>*TaFKBP22-5-6D*<br>*TaFKBP22-4-6D*<br>*TaFKBP20-2-6D*
**(b)**	***T. aestivum***	***T. dicoccoides***

1	*TaFKBP72-1-2D*<br>*TaFKBP20-6-2D*<br>*TaFKBP21-1-2D*<br>*TaFKBP53-1-2D*	*TdFKBP71-1-2B*<br>*TdFKBP29-1-2B*<br>*TdFKBP29-2-2B*<br>*TdFKBP53-1-2B*
2	*TaFKBP19-1-6A*<br>*TaFKBP21-5-6A*<br>*TaFKBP23-3-6A*<br>*TaFKBP22-2-6A*<br>*TaFKBP20-1-6A*	*TdFKBP17-3-6A*<br>*TdFKBP22-1-6A*<br>*TdFKBP26-1-6A*<br>*TdFKBP15-1-6A*<br>*TdFKBP20-1-6A*
3	*TaFKBP19-1-6A*<br>*TaFKBP21-5-6A*<br>*TaFKBP22-2-6A*<br>*TaFKBP20-1-6A*	*TdFKBP16-3-6B*<br>*TdFKBP21-2-6B*<br>*TdFKBP23-1-6B*<br>*TdFKBP21-1-6B*
4	*TaFKBP24-1-6B*<br>*TaFKBP21-6-6B*<br>*TaFKBP22-3-6B*<br>*TaFKBP20-3-6B*	*TdFKBP17-3-6A*<br>*TdFKBP22-1-6A*<br>*TdFKBP15-1-6A*<br>*TdFKBP20-1-6A*
**(c)**	***T. aestivum***	***T. aestivum***
1	*TaFKBP71-2-2A*<br>*TaFKBP29-1-2A*<br>*TaFKBP27-1-2A*<br>*TaFKBP48-1-2A*	*TaFKBP71-1-2B*<br>*TaFKBP24-2-2B*<br>*TaFKBP22-1-2B*<br>*TaFKBP53-2-2B*
2	*TaFKBP71-2-2A*<br>*TaFKBP29-1-2A*<br>*TaFKBP27-1-2A*<br>*TaFKBP48-1-2A*	*TaFKBP72-1-2D*<br>*TaFKBP20-6-2D*<br>*TaFKBP29-2-2D*<br>*TaFKBP53-1-2D*
3	*TaFKBP71-1-2B*<br>*TaFKBP24-2-2B*<br>*TaFKBP22-1-2B*<br>*TaFKBP65-1-2B*<br>*TaFKBP53-2-2B*	*TaFKBP72-1-2D*<br>*TaFKBP20-6-2D*<br>*TaFKBP29-2-2D*<br>*TaFKBP61-1-2D*<br>*TaFKBP53-1-2D*
4	*TaFKBP19-1-6A*<br>*TaFKBP21-5-6A*<br>*TaFKBP23-3-6A*<br>*TaFKBP22-2-6A*<br>*TaFKBP20-1-6A*	*TaFKBP24-1-6B*<br>*TaFKBP21-6-6B*<br>*TaFKBP23-1-6B*<br>*TaFKBP22-3-6B*<br>*TaFKBP20-3-6B*
5	*TaFKBP21-5-6A*<br>*TaFKBP23-3-6A*<br>*TaFKBP22-2-6A*<br>*TaFKBP20-1-6A*	*TaFKBP21-4-6D*<br>*TaFKBP22-5-6D*<br>*TaFKBP22-4-6D*<br>*TaFKBP20-2-6D*

6	*TaFKBP21-6-6B*<br>*TaFKBP23-1-6B*<br>*TaFKBP22-3-6B*<br>*TaFKBP20-3-6B*	*TaFKBP21-4-6D*<br>*TaFKBP22-5-6D*<br>*TaFKBP22-4-6D*<br>*TaFKBP20-2-6D*
**(d)**	***T. dicoccoides***	***T. dicoccoides***
1	*TdFKBP17-3-6A*<br>*TdFKBP22-1-6A*<br>*TdFKBP26-1-6A*<br>*TdFKBP15-1-6A*<br>*TdFKBP20-1-6A*	*TdFKBP16-3-6B*<br>*TdFKBP21-2-6B*<br>*TdFKBP24-1-6B*<br>*TdFKBP23-1-6B*<br>*TdFKBP21-1-6B*

Table S6. Location of *FKBP* genes on different chromosomes of *T. aestivum* and its progenitors. Green color boxes depict the *FKBP* genes whose number is conserved between *T. aestivum* and corresponding sub-genome donors. The red and blue color indicate the loss or gain of *FKBP* genes, respectively in the sub-genome donors compared to corresponding chromosomes in *T. aestivum*.

**Chromosome Number**	***T. aestivum***	***T. urartu***	***T. dicoccoides***	***A. tauschii***
1A	2	2	2	-
1B	2	-	1	-
1D	2	-	-	4
2A	6	4	4	-
2B	8	-	5	-
2D	6	-	-	5
3A	2	1	-	-
3B	1	-	1	-
3D	2	-	-	1
4A	1		1	-
4B	1	-	2	-
4D	1	-	-	1
5A	4	3	4	-
5B	4	-	5	-
5D	4	-	-	4
6A	6	2	6	-
6B	5	-	5	-
6D	4	-	-	4

7A	3	2	3	-
7B	3	-	3	-
7D	3	-	-	3

Table S7. Comparative analysis of parvulin *and PTPA* genes on different chromosomes of *T. aestivum* and its progenitors. Green color boxes indicate the number of genes conserved between the *T. aestivum* and the corresponding sub-genome donors. The red and blue color signify the loss and gain of genes, respectively in the sub-genomes relative to *T. aestivum*. Chromosomes not harbouring any *parvulin* or *PTPA* gene were not included in this table.

Chromosome Number	*T. aestivum*	*T. urartu*	*T. dicoccoides*	*A. tauschii*
***Parvulin* genes**				
2A	2	2	3	-
2B	2	-	3	-
2D	3	-	-	4
3B	1	-	1	-
4A	1	-	-	-
5A	1	1	1	-
5B	1	-	1	-
5D	1	-	-	1
***PTPA* genes**				
7A	1	1	-	-
7B	1	-	-	-
7D	1	-	-	1

Table S8. The active site residues present in FKBP-like domain of wheat FKBPs relative to the mammalian hFKBP12 [Van Duyne et al. (1993)]. (+ and – represent identical residue and residue not present, respectively).

S. No	Human hFKBP12	Active site residue as found in multiple sequence alignment with human FKBP12												
		Y(26)	F(36)	D(37)	R(42)	F(46)	F(48)	Q(53)	E(54)	I(56)	W(59)	Y(82)	H(87)	F(99)
1	TaFKBP7-1-2B	—	—	—	—	—	—	—	—	—	—	+	L	+
2	TaFKBP15-1-3D	+	I	V	+	+	+	G	Q	G	L	+	S	+
3	TaFKBP16-1-5B	+	+	+	+	I	+	G	Q	+	+	+	S	+
4	TaFKBP16-2-5D	+	+	+	+	I	+	G	Q	+	+	+	S	+
5	TaFKBP16-3-5A	+	+	+	+	I	+	G	Q	+	+	+	S	+
6	TaFKBP16-4-3D	+	+	+	+	+	+	G	Q	+	+	+	S	+
7	TaFKBP16-5-3A	+	+	+	+	+	+	G	Q	+	+	+	S	+
8	TaFKBP16-6-3B	+	+	+	+	+	+	G	Q	+	+	+	S	+
9	TaFKBP16-7-2B	W	+	E	G	+	+	G	Q	+	F	+	L	+
10	TaFKBP17-1-3A	+	+	+	+	+	+	G	Q	+	+	+	S	+
11	TaFKBP17-2-2A	+	+	+	T	+	+	G	K	-	—	-	-	-
12	TaFKBP18-1-6A	+	+	+	H	+	+	D	F	+	G	-	-	-
13	TaFKBP19-1-6A	F	V	S	I	-	-	-	-	-	—	-	-	-
14	TaFKBP20-1-6A	C	+	W	G	+	+	G	S	+	+	+	F	+
15	TaFKBP20-2-6D	C	+	W	G	+	+	G	S	+	+	+	F	+
16	TaFKBP20-3-6B	C	+	W	G	+	+	G	S	+	+	+	F	+
17	TaFKBP20-4-7B	+	+	+	+	L	I	G	+	+	+	+	A	+
18	TaFKBP20-5-7D	+	+	+	+	L	I	G	+	+	+	+	A	+
19	TaFKBP20-6-2D	W	+	E	-	_	-	-	-	+	F	+	L	+
20	TaFKBP20-7-7A	+	+	+	+	L	I	G	+	+	+	+	A	+
21	TaFKBP21-1-2D	+	I	+	Q	A	I	K	S	V	K	-	-	-
22	TaFKBP21-2-1D	+	+	+	D	+	+	G	A	+	+	+	S	+
23	TaFKBP21-3-1B	+	+	+	D	+	+	G	A	+	+	+	S	+
24	TaFKBP21-4-6D	+	+	+	T	+	+	G	K	+	I	+	Q	+
25	TaFKBP21-5-6A	+	+	+	T	+	+	G	K	+	I	+	Q	+
26	TaFKBP21-6-6B	+	+	+	T	+	+	R	K	+	I	+	Q	+
27	TaFKBP21-7-7B	C	+	+	K	Y	+	G	Q	+	L	I	S	-
28	TaFKBP22-1-2B	+	I	+	Q	A	I	K	S	V	F	P	-	+
29	TaFKBP22-2-6A	+	+	+	+	L	M	G	K	L	L	+	A	Y
30	TaFKBP22-3-6B	+	+	+	+	L	M	G	K	L	L	+	A	Y
31	TaFKBP22-4-6D	+	+	+	+	L	M	G	K	L	L	+	A	Y
32	TaFKBP22-5-6D	+	V	H	E	V	L	+	+	+	L	+	L	+
33	TaFKBP23-1-6B	+	V	H	E	V	L	+	+	+	L	+	L	+
34	TaFKBP23-2-5D	+	M	T	-	Y	+	S	+	G	L	+	V	L
35	TaFKBP23-3-6A	+	V	H	E	V	L	+	+	+	L	+	L	+
36	TaFKBP23-4-5A	+	M	T	-	Y	+	S	+	G	L	+	V	L

Continued…

37	TaFKBP23-5-5B	+	M	T	-	Y	+	S	+	G	L	+	V	L
38	TaFKBP23-6-U	F	V	S	I	Y	+	L	P	K	M	+	Q	L
39	TaFKBP23-7-7D	C	+	+	K	Y	+	G	Q	+	L	F	T	+
40	TaFKBP24-1-6B	F	V	S	I	Y	+	L	P	K	M	+	Q	L
41	TaFKBP24-2-2B	W	+	E	G	+	+	G	Q	+	F	+	L	+
42	TaFKBP24-3-7A	C	+	+	K	Y	+	G	Q	+	L	F	T	+
43	TaFKBP26-1-4D	L	+	V	G	L	L	R	P	T	M	F	A	Y
44	TaFKBP26-2-4A	L	+	V	G	L	L	R	P	T	M	F	A	Y
45	TaFKBP26-3-4B	L	+	V	G	L	L	R	P	T	M	F	A	Y
46	TaFKBP27-1-2A	+	I	+	Q	A	I	K	S	V	F	P	-	+
47	TaFKBP27-2-1A	+	+	+	D	+	+	G	A	+	+	+	S	+
48	TaFKBP29-1-2A	W	+	E	G	+	+	G	Q	+	F	+	L	+
49	TaFKBP29-2-2D	W	+	E	G	+	+	G	Q	+	F	+	L	+
50	TaFKBP36-1-5A	-	-	N	N	L	L	K	K	M	L	+	S	Y
51	TaFKBP41-1-5D	+	+	E	H	I	V	K	K	M	L	+	S	Y
52	TaFKBP42-1-5B	+	+	E	H	I	V	K	K	M	L	+	S	Y
53	TaFKBP47-1-5A	+	V	H	E	Y	+	E	K	M	+	D	L	Y
54	TaFKBP47-2-5B	+	V	H	E	Y	+	E	K	M	+	D	S	Y
55	TaFKBP47-3-5D	+	V	H	E	Y	+	E	K	M	+	N	S	Y
56	TaFKBP48-1-2A	+	+	+	G	+	+	G	Q	+	+	+	A	+
57	TaFKBP53-1-2D	+	+	+	G	+	+	G	Q	+	+	+	A	+
58	TaFKBP53-2-2B	+	+	+	G	+	+	G	Q	+	+	+	A	+
59	TaFKBP61-1-2D.1	+	+	+	+	+	+	G	Q	+	+	+	S	+
60	TaFKBP61-1-2D.2	+	V	S	-	I	+	G	Y	C	L	F	R	I
61	TaFKBP61-1-2D.3	L	+	T	G	+	+	E	Q	+	L	F	T	Y
62	TaFKBP61-2-7D.1	+	+	+	+	+	+	G	Q	+	+	+	A	+
63	TaFKBP61-2-7D.2	+	V	S	_	V	+	G	H	C	L	F	R	I
64	TaFKBP61-2-7D.3	I	+	L	E	+	+	E	A	+	L	+	S	Y
65	TaFKBP62-1-7A.1	+	+	+	+	+	+	G	Q	+	+	+	S	+
66	TaFKBP62-1-7A.2	+	V	S	-	V	+	G	H	C	L	F	R	I
67	TaFKBP62-1-7A.3	I	+	L	E	+	+	E	A	+	L	+	S	Y
68	TaFKBP62-2-7B.1	+	+	+	+	+	+	G	Q	+	+	+	S	+
69	TaFKBP62-2-7B.2	+	V	S	-	V	+	G	H	C	L	F	R	I
70	TaFKBP62-2-7B.3	I	+	L	E	+	+	E	A	+	L	+	S	y
71	TaFKBP63-1-1D.1	C	V	N	K	L	+	S	K	+	F	+	C	+
72	TaFKBP63-1-1D.2	I	I	L	K	Y	+	S	+	P	L	T	M	+
73	TaFKBP63-1-1D.3	+	Y	+	D	L	+	G	L	P	F	+	R	W
74	TaFKBP64-1-2A.1	+	+	+	+	+	+	G	Q	+	+	+	S	+

Continued…

75	TaFKBP64-1-2A.2	+	V	S	-	I	+	G	Y	C	L	F	R	I
76	TaFKBP64-1-2A.3	L	+	T	G	+	+	E	Q	+	L	F	T	Y
77	TaFKBP65-1-2B.1	+	+	+	+	+	+	G	Q	+	+	+	S	+
78	TaFKBP65-1-2B.2	+	V	S	-	I	+	G	Y	C	L	F	R	I
79	TaFKBP65-1-2B.3	L	+	T	G	+	+	E	Q	+	L	F	T	Y
80	TaFKBP67-1-1B.1	C	V	N	K	L	+	S	K	+	F	+	C	+
81	TaFKBP67-1-1B.2	I	I	L	K	Y	+	S	+	P	L	T	M	+
82	TaFKBP67-1-1B.3	+	Y	+	D	L	+	G	L	P	F	+	R	W
83	TaFKBP69-1-1A.1	C	V	N	K	L	+	S	K	+	F	+	C	+
84	TaFKBP69-1-1A.2	I	I	L	K	Y	+	S	+	P	L	T	M	+
85	TaFKBP69-1-1A.3	+	Y	+	D	L	+	G	L	P	F	+	R	W
86	TaFKBP71-1-2B.1	+	+	+	+	+	+	G	Q	+	+	+	S	+
87	TaFKBP71-1-2B.2	+	I	T	_	I	+	G	H	C	I	F	R	I
88	TaFKBP71-1-2B.3	L	+	V	G	+	+	D	Q	+	L	F	T	Y
89	TaFKBP71-2-2A.1	+	+	+	+	+	+	G	Q	+	+	+	S	+
90	TaFKBP71-2-2A.2	+	V	T	_	V	+	G	H	C	I	F	R	I
91	TaFKBP71-2-2A.3	L	+	V	G	+	+	D	Q	+	L	F	T	Y
92	TaFKBP72-1-2D.1	+	+	+	+	+	+	G	Q	+	+	+	S	+
93	TaFKBP72-1-2D.2	+	I	T	-	I	+	G	H	C	I	F	R	I
94	TaFKBP72-1-2D.3	L	+	V	G	+	+	D	Q	+	L	F	T	Y
95	TaFKBP77-1-2B	+	I	E	S	H	+	G	M	F	+	+	S	-

Table S9. Different active site residues present in rotamase domain of wheat parvulin proteins relative to human parvulin hPar14 [Sekerina et al. (2000)]. (+ and – represent identical residue and residue not present, respectively).

Sr. no	Human Parvulin14 (hPar14)	Active site residue as found in multiple sequence alignment with hPar14							
		H(42)	S(72)	D(74)	L(82)	M(90)	F(94)	T(118)	H(123)
1	TaPar11-1-3B	+	+	P	R	I	-	-	-
2	TaPar11-2-4A	+	+	P	R	I	-	-	-
3	TaPar12-1-2D	+	+	N	+	+	+	+	+
4	TaPar13-1-2D	+	+	S	+	+	+	+	+
5	TaPar13-2-2A	+	+	S	+	+	+	+	+
6	TaPar13-3-2B	+	+	S	+	+	+	+	+
7	TaPar15-1-5D	+	+	P	+	+	+	S	+
8	TaPar15-2-5B	+	+	P	+	+	+	S	+
9	TaPar15-3-5A	+	+	P	+	+	+	S	+
10	TaPar32-1-2D	+	+	P	+	+	+	+	+
11	TaPar32-2-2B	+	+	P	+	+	+	+	+
12	TaPar32-3-2A	+	+	P	+	+	+	+	+

Table S10. Primer pairs used for qRT-PCR analysis of *TaFKBP* and *TaPar* genes of *T. aestivum.*

S. No.	Gene name	Primer Sequences (F, R [5'-3'])
TaFKBPs		
1	*TaFKBP23-2-5D*	CCTCGGTGTCACTGGTGGAA, ACCAACCTCTGCCCTCCAAC
	TaFKBP23-4-5A	
	TaFKBP23-5-5B	
2	*TaFKBP7-1-2B*	CATGCGGCTAGGTGGAGTT, ATCTAGGGCTCTTTGTCCCG
	TaFKBP16-7-2B	
	TaFKBP24-2-2B	
	TaFKBP29-1-2A	
	TaFKBP29-2-2D	
	TaFKBP20-6-2D	
3	*TaFKBP48-1-2A*	ACCAGGGTAGAGGCAAGAGT, ATTCAGCAGGTGGGGTCCTA
	TaFKBP53-1-2D	
	TaFKBP53-2-2B	
4	*TaFKBP63-1-1D*	TCCTTGGCTTTGCTGAAGGCT, TGGGAAGCCATCTGGAGGTGT
	TaFKBP67-1-1B	
	TaFKBP69-1-1A	
5	*TaFKBP61-2-7D*	ACACATTCAAGTTCAAGCTGGG, GCCGGAATAGTTGGAGGTGA
	TaFKBP62-1-7A	
	TaFKBP62-2-7B	
6	*TaFKBP21-7-7B*	CGCCCATTGGCTTCCAGGTT, ATCACCTGTCCAGCGCCAAC

	TaFKBP23-7-7D	
	TaFKBP24-3-7A	
7	*TaFKBP71-1-2B*	TGTGGCCTCTAGCGCCTTTC, GCAACACAAAGCGCGACCAA
	TaFKBP71-2-2A	
	TaFKBP72-1-2D	
8	*TaFKBP16-1-5B*	ACCGTGGAACACTTACCGAT, CACCAATGCACATGCCCAAA
	TaFKBP16-2-5D	
	TaFKBP16-3-5A	
9	*TaFKBP21-4-6D*	TCCATGAGAGTCGGTGGTCT, GCCATTTGCAAGGCGTGT
	TaFKBP21-5-6A	
	TaFKBP21-6-6B	
10	*TaFKBP47-1-5A*	AACAAGTTGACAGCTCCGCT, AGCCAAGGAAACAGGCGTAG
	TaFKBP47-2-5B	
	TaFKBP47-3-5D	
11	*TaFKBP21-1-2D*	CTACGCCCACGCTCTCACTG, GCACGGTTGGCTTCCTGGAT
	TaFKBP22-1-2B	
	TaFKBP27-1-2A	
12	*TaFKBP20-1-6A*	TCAGAAGCCACCACGCCAAC, CGGTGACCTTCTGGCCCTTG
	TaFKBP20-2-6D	
	TaFKBP20-3-6B	
13	*TaFKBP16-4-3D*	CGTCCTTCTGACCGCTTCGG, CGCCTTTGTGAGCTTGTAGGGT
	TaFKBP16-5-3A	
	TaFKBP16-6-3B	
14	*TaFKBP36-1-5A*	ACGAAACCAACGTGAAAGCG, TTCCGGGGAGTGTTTCTTGG
TaPars		

1	*TaPar32-1-2D*	GAGAGAGCATCCCTTCCAGG, TGCCATGGTGACACAGAACA
	TaPar32-2-2B	
	TaPar32-3-2A	
2	*TaPar15-1-5D*	AAGGTGGGGATCTTGGATGG, CTTCCTGCCTTCACAGAGGA
	TaPar15-2-5B	
	TaPar15-3-5A	
3	*TaPar13-1-2D*	CACTCCGACTGCTCCTCC, GATCTCCCCCACCTTGAGAG
	TaPar13-2-2A	
	TaPar13-3-2B	

Table S11. Stress responsive elements in the upstream region (2.0 kb) of genes encoding wheat FKBPs, parvulins and PTPAs (STRE: stress responsive element; HSE: heat shock element).

S. No	Genes	STRE	TTC rich type 1	TTC rich type 2	TTC rich type 3	TTC rich type 4	Gap type 1	Gap type 2	Gap type 3	Perfect HSE
TaFKBPs										
1	*TaFKBP7-1-2B*	5	-	-	-	-	-	-	-	-
2	*TaFKBP15-1-3D*	6	-	-	-	-	-	-	-	-
3	*TaFKBP16-1-5B*	2	-	-	-	3	-	-	-	-
4	*TaFKBP16-2-5D*	2	-	-	-	3	-	-	-	-
5	*TaFKBP16-3-5A*	5	-	-	-	3	-	1	-	-
6	*TaFKBP16-4-3D*	2	-	-	-	5	1	-	-	-
7	*TaFKBP16-5-3A*	2	-	-	-	-	-	-	-	-
8	*TaFKBP16-6-3B*	2	-	-	-	3	-	-	-	-
9	*TaFKBP16-7-2B*	3	-	-	-	1	-	-	-	-
10	*TaFKBP17-1-3A*	-	1	-	-	5	-	1	-	-
11	*TaFKBP17-2-2A*	10	-	-	-	-	-	-	-	-
12	*TaFKBP18-1-6A*	2	-	-	-	1	-	1	-	-
13	*TaFKBP19-1-6A*	4	-	-	-	2	-	-	-	-
14	*TaFKBP20-1-6A*	-	-	-	-	2	-	-	-	1
15	*TaFKBP20-2-6D*	-	-	-	-	-	-	-	-	-
16	*TaFKBP20-3-6B*	2	-	-	-	3	-	-	-	-
17	*TaFKBP20-4-7B*	6	-	-	-	1	-	-	-	-
18	*TaFKBP20-5-7D*	4	-	-	-	1	-	-	-	-
19	*TaFKBP20-6-2D*	3	1	-	-	6	-	-	-	-
20	*TaFKBP20-7-7A*	1	-	-	-	2	-	1	-	-
21	*TaFKBP21-1-2D*	2	-	-	-	3	-	-	-	-
22	*TaFKBP21-2-1D*	7	-	-	-	2	-	-	-	-
23	*TaFKBP21-3-1B*	6	-	-	-	2	-	-	-	-
24	*TaFKBP21-4-6D*	2	-	-	-	2	-	-	-	-
25	*TaFKBP21-5-6A*	4	-	-	-	1	-	-	-	-
26	*TaFKBP21-6-6B*	8	-	-	-	2	-	-	-	-
27	*TaFKBP21-7-7B*	-	-	-	-	-	-	-	-	-
28	*TaFKBP22-1-2B*	2	-	-	-	1	2	1	-	4
29	*TaFKBP22-2-6A*	4	-	-	-	2	-	-	-	-
30	*TaFKBP22-3-6B*	4	-	-	-	2	-	-	-	-
31	*TaFKBP22-4-6D*	-	-	-	-	-	-	-	-	-
32	*TaFKBP22-5-6D*	1	-	-	-	-	-	-	-	-
33	*TaFKBP23-1-6B*	10	-	-	-	-	-	-	1	-
34	*TaFKBP23-2-5D*	8	-	-	-	7	-	-	-	-
35	*TaFKBP23-3-6A*	-	-	-	-	-	-	-	-	-
36	*TaFKBP23-4-5A*	4	-	-	-	2	-	-	-	-
37	*TaFKBP23-5-5B*	6	-	-	-	2	-	-	-	1
38	*TaFKBP23-6-U*	3	-	-	-	2	-	-	-	-
39	*TaFKBP23-7-7D*	-	-	-	-	-	-	-	-	-
40	*TaFKBP24-1-6B*	3	-	-	-	1	-	-	-	-

Continued…

41	*TaFKBP24-2-2B*	3	1	-	-	5	-	-	-	-
42	*TaFKBP24-3-7A*	5	-	-	-	3	-	-	-	-
43	*TaFKBP26-1-4D*	2	-	-	-	1	-	-	-	-
44	*TaFKBP26-2-4A*	4	-	-	-	-	-	-	-	-
45	*TaFKBP26-3-4B*	7	-	-	-	-	-	-	-	-
46	*TaFKBP27-1-2A*	3	-	-	-	6	1	-	-	-
47	*TaFKBP27-2-1A*	2	-	-	-	1	-	-	-	-
48	*TaFKBP29-1-2A*	2	-	-	-	5	1	-	-	-
49	*TaFKBP29-2-2D*	8	-	-	-	2	-	-	-	-
50	*TaFKBP36-1-5A*	6	-	-	-	2	-	-	-	-
51	*TaFKBP41-1-5D*	6	-	-	-	1	-	-	-	-
52	*TaFKBP42-1-5B*	10	-	1	-	1	1	-	-	-
53	*TaFKBP47-1-5A*	3	-	-	-	-	-	-	-	-
54	*TaFKBP47-2-5B*	6	-	-	-	2	-	-	-	-
55	*TaFKBP47-3-5D*	1	-	-	-	-	-	-	-	-
56	*TaFKBP48-1-2A*	3	1	-	-	2	-	-	-	-
57	*TaFKBP53-1-2D*	3	-	-	-	1	1	-	-	-
58	*TaFKBP53-2-2B*	5	-	-	-	2	1	-	-	-
59	*TaFKBP61-1-2D*	-	-	-	-	9	-	-	-	2
60	*TaFKBP61-2-7D*	3	-	-	-	2	-	1	-	-
61	*TaFKBP62-1-7A*	7	1	-	-	2	-	-	-	-
62	*TaFKBP62-2-7B*	4	-	-	-	1	-	-	-	-
63	*TaFKBP63-1-1D*	5	-	-	-	1	-	-	-	-
64	*TaFKBP64-1-2A*	-	-	-	-	-	-	-	-	-
65	*TaFKBP65-1-2B*	-	-	-	-	-	-	-	-	-
66	*TaFKBP67-1-1B*	4	-	-	-	-	-	-	-	-
67	*TaFKBP69-1-1A*	2	-	-	-	4	-	-	-	-
68	*TaFKBP71-1-2B*	4	-	1	-	-	-	1	-	-
69	*TaFKBP71-2-2A*	-	-	-	-	-	-	-	-	-
70	*TaFKBP72-1-2D*	3	-	-	-	-	-	-	-	-
71	*TaFKBP77-1-2B*	3	-	-	-	1	1	-	-	1
TaPars										
1	*TaPar11-1-3B*	9	-	-	-	1	-	-	-	-
2	*TaPar11-2-4A*	3	-	-	-	2	-	-	-	-
3	*TaPar12-1-2D*	10	1	-	-	4	-	-	-	-
4	*TaPar13-1-2D*	8	-	-	-	2	1	-	-	-
5	*TaPar13-2-2A*	4	-	-	-	2	-	-	-	-
6	*TaPar13-3-2B*	9	-	-	-	7	-	-	-	-
7	*TaPar15-1-5D*	6	-	-	-	4	-	-	-	-
8	*TaPar15-2-5B*	23	1	1	-	3	-	-	-	-
9	*TaPar15-3-5A*	16	-	-	-	2	-	-	-	-
10	*TaPar32-1-2D*	11	-	-	-	4	-	-	-	-

Continued…

11	*TaPar32-2-2B*	11	-	-	-	2	-	-	-	-
12	*TaPar32-3-2A*	14	-	-	-	4	-	-	-	-
TaPTPAs										
1	*TaPTPA41-1-7D**	3	-	-	-	6	-	-	-	1
2	*TaPTPA42-1-7B*	6	-	-	1	-	-	-	-	-
3	*TaPTPA42-2-7A*	3	-	-	-	3	-	-	-	1

Table S12: Details of TaFKBP, TaPar and TaPTPA proteins mapping to their orthologue proteins in *Arabidopsis thaliana*.

S. No.	Query protein name	String Id	Identity (%)	Bit score	Name of the corresponding Orthologue in *Arabidopsis*
**TaFKBPs**					
1	TaFKBP7-1-2B	3702.AT5G13410.1	76.1	116.7	AT5G13410
2	TaFKBP15-1-3D	3702.AT3G25220.1	44.7	106.3	FKBP15-1
3	TaFKBP16-1-5B	3702.AT3G25220.1	84.2	223	FKBP15-1
4	TaFKBP16-2-5D	3702.AT3G25220.1	84.3	225.3	FKBP15-1
5	TaFKBP16-3-5A	3702.AT3G25220.1	79.6	229.6	FKBP15-1
6	TaFKBP16-4-3D	3702.AT3G25220.1	72.3	214.5	FKBP15-1
7	TaFKBP16-5-3A	3702.AT3G25220.1	70.6	211.8	FKBP15-1
8	TaFKBP16-6-3B	3702.AT3G25220.1	71.8	213.8	FKBP15-1
9	TaFKBP16-7-2B	3702.AT5G13410.1	80	264.2	AT5G13410
10	TaFKBP17-1-3A	3702.AT3G25220.1	72.2	176.4	FKBP15-1
11	TaFKBP17-2-2A	3702.AT3G25230.2	54.3	87	ROF1
12	TaFKBP18-1-6A	3702.AT5G48570.1	59.2	87.4	ROF2
13	TaFKBP19-1-6A	3702.AT1G20810.1	71.2	94.7	AT1G20810
14	TaFKBP20-1-6A	3702.AT5G64350.1	76.8	196.1	FKBP12
15	TaFKBP20-2-6D	3702.AT5G64350.1	76.8	196.8	FKBP12
16	TaFKBP20-3-6B	3702.AT5G64350.1	76.8	196.4	FKBP12
17	TaFKBP20-4-7B	3702.AT5G45680.1	69.5	196.1	FKBP13
18	TaFKBP20-5-7D	3702.AT5G45680.1	68.8	195.3	FKBP13
19	TaFKBP20-6-2D	3702.AT5G13410.1	68.3	276.2	AT5G13410
20	TaFKBP20-7-7A	3702.AT5G45680.1	68.8	195.3	FKBP13
21	TaFKBP21-1-2D	3702.AT3G60370.1	67.4	192.2	AT3G60370
22	TaFKBP21-2-1D	3702.AT3G55520.1	74.1	228.4	AT3G55520
23	TaFKBP21-3-1B	3702.AT3G55520.1	74.1	227.6	AT3G55520
24	TaFKBP21-4-6D	3702.AT4G19830.1	79.6	261.2	AT4G19830
25	TaFKBP21-5-6A	3702.AT4G19830.1	79.6	261.2	AT4G19830
26	TaFKBP21-6-6B	3702.AT4G19830.1	80.3	262.3	AT4G19830
27	TaFKBP21-7-7B	3702.AT2G43560.1	76.4	198.4	AT2G43560
28	TaFKBP22-1-2B	3702.AT3G60370.1	72	302.8	AT3G60370

29	TaFKBP22-2-6A	3702.AT4G39710.1	69.5	240.4	PnsL4
30	TaFKBP22-3-6B	3702.AT4G39710.1	68.3	238.4	PnsL4
31	TaFKBP22-4-6D	3702.AT4G39710.1	69.5	240.4	PnsL4
32	TaFKBP22-5-6D	3702.AT4G26555.1	73.3	208.8	AT4G26555
33	TaFKBP23-1-6B	3702.AT4G26555.1	63.8	212.6	AT4G26555
34	TaFKBP23-2-5D	3702.AT3G10060.1	91.1	273.5	AT3G10060
35	TaFKBP23-3-6A	3702.AT4G26555.1	71.7	213.4	AT4G26555
36	TaFKBP23-4-5A	3702.AT3G10060.1	83.3	279.3	AT3G10060
37	TaFKBP23-5-5B	3702.AT3G10060.1	83.3	279.3	AT3G10060
38	TaFKBP23-6-U	3702.AT1G20810.1	73.6	232.6	AT1G20810
39	TaFKBP23-7-7D	3702.AT2G43560.1	80.4	262.3	AT2G43560
40	TaFKBP24-1-6B	3702.AT1G20810.1	70.8	232.3	AT1G20810
41	TaFKBP24-2-2B	3702.AT5G13410.1	72.8	309.7	AT5G13410
42	TaFKBP24-3-7A	3702.AT2G43560.1	78	265	AT2G43560
43	TaFKBP26-1-4D	3702.AT1G18170.1	71	269.6	AT1G18170
44	TaFKBP26-2-4A	3702.AT1G18170.1	70.4	267.7	AT1G18170
45	TaFKBP26-3-4B	3702.AT1G18170.1	70.1	263.5	AT1G18170
46	TaFKBP27-1-2A	3702.AT3G60370.1	73	307	AT3G60370
47	TaFKBP27-2-1A	3702.AT3G55520.1	74.1	227.3	AT3G55520
48	TaFKBP29-1-2A	3702.AT5G13410.1	74.5	312	AT5G13410
49	TaFKBP29-2-2D	3702.AT5G13410.1	74.5	312	AT5G13410
50	TaFKBP36-1-5A	3702.AT3G21640.1	68.8	373.2	TWD1
51	TaFKBP41-1-5D	3702.AT3G21640.1	68	481.5	TWD1
52	TaFKBP42-1-5B	3702.AT3G21640.1	66.4	485	TWD1
53	TaFKBP47-1-5A	3702.AT3G12340.1	35.1	163.7	AT3G12340
54	TaFKBP47-2-5B	3702.AT3G12340.1	35.7	164.5	AT3G12340
55	TaFKBP47-3-5D	3702.AT3G12340.1	34.9	162.9	AT3G12340
56	TaFKBP48-1-2A	3702.AT4G25340.1	72.8	157.1	FKBP53
57	TaFKBP53-1-2D	3702.AT4G25340.1	41.1	180.3	FKBP53
58	TaFKBP53-2-2B	3702.AT4G25340.1	39.3	177.6	FKBP53
59	TaFKBP61-1-2D	3702.AT5G48570.1	72.6	800.8	ROF2
60	TaFKBP61-2-7D	3702.AT5G48570.1	73.8	813.9	ROF2
61	TaFKBP62-1-7A	3702.AT5G48570.1	74	813.9	ROF2
62	TaFKBP62-2-7B	3702.AT5G48570.1	71.7	782.7	ROF2
63	TaFKBP63-1-1D	3702.AT3G54010.1	57.4	655.6	PAS1
64	TaFKBP64-1-2A	3702.AT5G48570.1	68.8	773.1	ROF2
65	TaFKBP65-1-2B	3702.AT3G25230.2	74.1	685.6	ROF1
66	TaFKBP67-1-1B	3702.AT3G54010.1	62.9	755	PAS1
67	TaFKBP69-1-1A	3702.AT3G54010.1	68.7	754.2	PAS1
68	TaFKBP71-1-2B	3702.AT5G48570.1	64.9	719.2	ROF2
69	TaFKBP71-2-2A	3702.AT5G48570.1	67.7	734.9	ROF2

70	TaFKBP72-1-2D	3702.AT5G48570.1	71.7	772.3	ROF2
**TaPars**					
1	TaPar11-1-3B	3702.AT2G18040.1	47.5	60.8	PIN1AT
2	TaPar11-2-4A	3702.AT2G18040.1	47.5	60.5	PIN1AT
3	TaPar12-1-2D	3702.AT2G18040.1	61.6	144.4	PIN1AT
4	TaPar13-1-2D	3702.AT2G18040.1	67.8	162.9	PIN1AT
5	TaPar13-2-2A	3702.AT2G18040.1	67.8	162.9	PIN1AT
6	TaPar13-3-2B	3702.AT2G18040.1	67.8	162.2	PIN1AT
7	TaPar15-1-5D	3702.AT1G26550.1	90.6	215.3	AT1G26550
8	TaPar15-2-5B	3702.AT1G26550.1	90.6	215.3	AT1G26550
9	TaPar15-3-5A	3702.AT1G26550.1	89.6	213	AT1G26550
10	TaPar32-1-2D	3702.AT5G19370.1	63.4	302.4	AT5G19370
11	TaPar32-2-2B	3702.AT5G19370.1	61.2	299.7	AT5G19370
12	TaPar32-3-2A	3702.AT5G19370.1	54.9	296.6	AT5G19370
**TaPTPAs**					
1	TaPTPA41-1-7D*	3702.AT4G08960.1	57.8	445.3	AT4G08960
2	TaPTPA42-1-7B	3702.AT4G08960.1	58.1	448.4	AT4G08960
3	TaPTPA42-2-7A	3702.AT4G08960.1	59.1	454.5	AT4G08960

Table S13: Degree of Interaction of TaFKBPs

#node	identifier	node_degree
TaFKBPs		
ABCB1	3702.AT2G36910.1	2
ABCB19	3702.AT3G28860.1	2
CYP38	3702.AT3G01480.1	23
HSFA2	3702.AT2G26150.1	8
HSP90.1	3702.AT5G52640.1	15
LTO1	3702.AT4G35760.1	5
PIN1AT	3702.AT2G18040.1	22
PnsB5	3702.AT5G43750.1	8
Pnsl5	3702.AT5G13120.1	23
TOR	3702.AT1G50030.1	20
TaFKBP17-1-3A	3702.AT3G25220.1	6
TaFKBP20-3-6B	3702.AT5G64350.1	19
TaFKBP20-7-7A	3702.AT5G45680.1	19
TaFKBP21-6-6B	3702.AT4G19830.1	7
TaFKBP22-4-6D	3702.AT4G39710.1	17
TaFKBP23-3-6A	3702.AT4G26555.1	8
TaFKBP23-5-5B	3702.AT3G10060.1	15
TaFKBP24-1-6B	3702.AT1G20810.1	14
TaFKBP24-3-7A	3702.AT2G43560.1	14

TaFKBP26-3-4B	3702.AT1G18170.1	13
TaFKBP27-1-2A	3702.AT3G60370.1	12
TaFKBP27-2-1A	3702.AT3G55520.1	12
TaFKBP29-2-2D	3702.AT5G13410.1	12
TaFKBP42-1-5B	3702.AT3G21640.1	10
TaFKBP47-3-5D	3702.AT3G12340.1	6
TaFKBP53-2-2B	3702.AT4G25340.1	6
TaFKBP65-1-2B	3702.AT3G25230.2	8
TaFKBP69-1-1A	3702.AT3G54010.1	7
TaFKBP72-1-2D	3702.AT5G48570.1	7
TaPars		
AT2G43760	3702.AT2G43760.1	2
AT2G45695	3702.AT2G45695.1	4
AT3G48860	3702.AT3G48860.2	1
AT3G61113	3702.AT3G61113.1	3
AT4G35910	3702.AT4G35910.1	3
FKBP12	3702.AT5G64350.1	4
NRPB1	3702.AT4G35800.1	3
NRPD1A	3702.AT1G63020.2	3
NRPD1B	3702.AT2G40030.1	5
PP2A	3702.AT1G69960.1	3
TaPar13-3-2B	3702.AT2G18040.1	6
TaPar15-3-5A	3702.AT1G26550.1	7
TaPar32-3-2A	3702.AT5G19370.1	4
TaPTPAs		
AT4G10050	3702.AT4G10050.1	10
AT5G16210	3702.AT5G16210.1	7
PP2A	3702.AT1G69960.1	7
PP2A-1	3702.AT1G59830.1	7
PP2A-3	3702.AT2G42500.1	8
PP2A-4	3702.AT3G58500.1	8
PP2AA2	3702.AT3G25800.1	9
PP2AA3	3702.AT1G13320.3	9
RCN1	3702.AT1G25490.1	9
TAP46	3702.AT5G53000.1	10
TaPTPA42-2-7A	3702.AT4G08960.1	10

Table S14: k-means clustering details of Protein-protein interaction network.

Cluster Number	Cluster Color	Gene Count	Protein Name	Protein Identifier	Protein Description (short)
**TaFKBPs**					
1	Red	13	CYP38	3702.AT3G01480.1	Peptidyl-prolyl cis-trans isomerase. Encodes a chloroplast cyclophilin functioning in the assembly and maintenance of photosystem II (PSII) supercomplexes
1	Red	13	LTO1	3702.AT4G35760.1	Nad(p)h dehydrogenase (quinone)s; Thiol-disulfide oxidoreductase catalyzing disulfide bond formation of chloroplast proteins and involved in redox regulation and photosynthetic electron transport.
1	Red	13	PnsB5	3702.AT5G43750.1	Photosynthetic ndh subcomplex b 5; NDH shuttles electrons from NAD(P)H:plastoquinone, via FMN and iron-sulfur (Fe-S) centers, to quinones in the photosynthetic chain and possibly in a chloroplast respiratory chain.
1	Red	13	Pnsl5	3702.AT5G13120.1	Peptidyl-prolyl cis-trans isomerase b (cyclophilin b); NDH shuttles electrons from NAD(P)H:plastoquinone, via FMN and iron-sulfur (Fe-S) centers, to quinones in the photosynthetic chain and possibly in a chloroplast respiratory chain.
1	Red	13	TaFKBP20-7-7A	3702.AT5G45680.1	Peptidyl-prolyl cis-trans isomerase FKBP13, chloroplastic; PPIases accelerate the folding of proteins.
1	Red	13	TaFKBP23-3-6A	3702.AT4G26555.1	
1	Red	13	TaFKBP23-5-5B	3702.AT3G10060.1	
1	Red	13	TaFKBP24-1-6B	3702.AT1G20810.1	
1	Red	13	TaFKBP24-3-7A	3702.AT2G43560.1	
1	Red	13	TaFKBP26-3-4B	3702.AT1G18170.1	
1	Red	13	TaFKBP27-1-2A	3702.AT3G60370.1	Encodes an immunophilin, FKBP20-2 which contains a unique pair of cysteines at the C terminus and was found to be reduced by thioredoxin (Trx).
1	Red	13	TaFKBP27-2-1A	3702.AT3G55520.1	FKBP-like peptidyl-prolyl cis-trans isomerase family protein
1	Red	13	TaFKBP53-2-2B	3702.AT4G25340.1	Peptidyl-prolyl cis-trans isomerase FKBP5, accelerate the folding of proteins. Histone chaperone possibly involved in H3/H4 deposition to the nucleosome. Associates with 18S rDNA

					chromatin and negatively regulates the level of its expression.
2	Green	12	ABCB1	3702.AT2G36910.1	ABC transporter B family member 1; Auxin efflux transporter that acts as a negative regulator of light signaling to promote hypocotyl elongation. Mediates the accumulation of chlorophyll and anthocyanin, as well as the expression of genes in response to light.
2	Green	12	ABCB19	3702.AT3G28860.1	
2	Green	12	PIN1AT	3702.AT2G18040.1	Peptidylprolyl cis/trans isomerase, NIMA-interacting 1 with specificity for phosphoserine-proline bonds
2	Green	12	TOR	3702.AT1G50030.1	Serine/threonine-protein kinase TOR; Essential cell growth regulator that controls development from early embryo to seed production. Controls plant growth in environmental stress conditions. Acts through the phosphorylation of downstream effectors that are recruited by the binding partner RAPTOR.
2	Green	12	TaFKBP17-1-3A	3702.AT3G25220.1	Peptidyl-prolyl cis-trans isomerase FKBP15-1
2	Green	12	TaFKBP20-3-6B	3702.AT5G64350.1	Arabidopsis thaliana fk506-binding protein 12, Mediates rapamycin inactivation of TOR protein kinase activity
2	Green	12	TaFKBP21-6-6B	3702.AT4G19830.1	FKBP-like peptidyl-prolyl cis-trans isomerase family protein
2	Green	12	TaFKBP22-4-6D	3702.AT4G39710.1	NDH shuttles electrons from NAD(P)H:plastoquinone, via FMN and iron-sulfur (Fe-S) centers, to quinones in the photosynthetic chain and possibly in a chloroplast respiratory chain.
2	Green	12	TaFKBP29-2-2D	3702.AT5G13410.1	FKBP-like peptidyl-prolyl cis-trans isomerase family protein
2	Green	12	TaFKBP42-1-5B	3702.AT3G21640.1	FKBP-type peptidyl-prolyl cis-trans isomerase family protein, Modulates the uptake of MRP substrates into the vacuole; reduces metolachlor-GS (MOC-GS) and enhances 17-beta-estradiol 17-(beta-D-glucuronide) (E(2)17betaG) uptake. Regulates cell elongation and orientation. Functions as a positive regulator of PGP1-mediated auxin transport.
2	Green	12	TaFKBP47-3-5D	3702.AT3G12340.1	FKBP-like peptidyl-prolyl cis-trans isomerase family protein
2	Green	12	TaFKBP69-1-1A	3702.AT3G54010.1	FKBP-type peptidyl-prolyl cis-trans isomerase family protein, Essential

					protein regulating cell division, adhesion and elongation throughout the plant development and embryogenesis. Required for the spatial organization of apical meristems. Involved in the hormonal control of cell division and differentiation mediated by cytokinins and auxin.
3	Blue	4	HSFA2	3702.AT2G26150.1	Heat stress transcription factor A-2; Transcriptional activator that specifically binds DNA sequence 5'-AGAAnnTTCT-3' known as heat shock promoter elements (HSE). Involved in heat stress responses
3	Blue	4	HSP90.1	3702.AT5G52640.1	Heat shock protein 90-1; Functions as a holding molecular chaperone (holdase) as well as folding molecular chaperone
3	Blue	4	TaFKBP65-1-2B	3702.AT3G25230.2	Peptidyl-prolyl cis-trans isomerase FKBP62; Co-chaperone that positively modulates thermotolerance by interacting with HSP90.1 and increasing the HSFA2-mediated accumulation of chaperones of the small-HSPs family.
3	Blue	4	TaFKBP72-1-2D	3702.AT5G48570.1	PPIases accelerate the folding of proteins. Co-chaperone that negatively modulates thermotolerance by interacting with FKBP62 and decreasing the HSFA2-mediated accumulation of chaperones of the small-HSPs family. Plays a positive role in tolerance to intracellular acid stress by maintaining the pH homeostasis. May be a part of transcription regulation pathways upon pathogen infection.
**TaPars**					
1	Red	5	AT2G43760	3702.AT2G43760.1	Molybdopterin biosynthesis MoaE family protein; Catalytic subunit of the molybdopterin synthase complex that catalyzes the conversion of precursor Z into molybdopterin.
1	Red	5	AT2G45695	3702.AT2G45695.1	Ubiquitin-related modifier 1 homolog 1; Acts as a sulfur carrier required for 2-thiolation of mcm(5)S(2)U at tRNA wobble positions of cytosolic tRNA(Lys), tRNA(Glu) and tRNA(Gln). Also acts as a ubiquitin-like protein (UBL) that is covalently conjugated via an isopeptide bond to lysine residues of target proteins.
1	Red	5	AT3G61113	3702.AT3G61113.1	

1	Red	5	AT4G35910	3702.AT4G35910.1	Adenine nucleotide alpha hydrolases-like superfamily protein; Plays a central role in 2-thiolation of mcm(5)S(2)U at tRNA wobble positions of tRNA(Lys), tRNA(Glu) and tRNA(Gln)
1	Red	5	TaPar32-3-2A	3702.AT5G19370.1	Rhodanese-like domain-containing protein / PPIC-type PPIASE domain-containing protein
2	Green	6	FKBP12	3702.AT5G64350.1	Arabidopsis thaliana fk506-binding protein 12
2	Green	6	NRPB1	3702.AT4G35800.1	DNA-directed RNA polymerase II subunit 1; DNA-dependent RNA polymerase catalyzes the transcription of DNA into RNA using the four ribonucleoside triphosphates as substrates.
2	Green	6	NRPD1A	3702.AT1G63020.2	DNA-directed RNA polymerase IV subunit 1; DNA-dependent RNA polymerase.
2	Green	6	NRPD1B	3702.AT2G40030.1	DNA-directed RNA polymerase V subunit 1
2	Green	6	PP2A	3702.AT1G69960.1	Serine/threonine-protein phosphatase PP2A-5 catalytic subunit; Associates with the serine/threonine-protein phosphatase PP2A regulatory subunits A and B' to positively regulates beta-oxidation of fatty acids and protoauxins in peroxisomes by dephosphorylating peroxisomal beta-oxidation-related proteins. Involved in the positive regulation of salt stress responses.
2	Green	6	TaPar13-3-2B	3702.AT2G18040.1	Peptidyl-prolyl cis-trans isomerase nima-interacting 1
3	Blue	2	AT3G48860	3702.AT3G48860.2	Stomatal cytokinesis defective 2; Involved in growth and development through its role in cytokinesis and polarized cell expansion.
3	Blue	2	TaPar15-3-5A	3702.AT1G26550.1	FKBP-like peptidyl-prolyl cis-trans isomerase family protein.
**TaPTPAs**					
1	Red	4	PP2A-4	3702.AT3G58500.1	Serine/threonine-protein phosphatase pp2a-3 catalytic subunit; Functions redundantly with PP2A3, and is involved in establishing auxin gradients, apical-basal axis of polarity and root and shoot apical meristem during embryogenesis.
1	Red	4	PP2AA2	3702.AT3G25800.1	Serine/threonine-protein phosphatase 2A 65 kDa regulatory subunit A beta isoform; serves as a scaffolding molecule to coordinate the assembly of the
1	Red	4	PP2AA3	3702.AT1G13320.3	
1	Red	4	RCN1	3702.AT1G25490.1	

					catalytic subunit and a variable regulatory B subunit. Involved during developmental process such as seedling and floral developments.
2	Green	4	PP2A	3702.AT1G69960.1	Serine/threonine-protein phosphatase PP2A-5 catalytic subunit.
2	Green	4	PP2A-1	3702.AT1G59830.1	Encodes one of the isoforms of the catalytic subunit of protein phosphatase 2A.
2	Green	4	PP2A-3	3702.AT2G42500.1	Serine/threonine-protein phosphatase PP2A-3 catalytic subunit; Functions redundantly with PP2A3, and is involved in establishing auxin gradients, apical-basal axis of polarity and root and shoot apical meristem during embryogenesis.
2	Green	4	TAP46	3702.AT5G53000.1	2A phosphatase associated protein of 46 kD; Involved in the positive regulation of the TOR signaling pathway. Acts as a negative regulator of PP2A catalytic activity. Plays a positive role in the ABA-regulated inhibition of germination, probably throught its interaction with ABI5.
3	Blue	3	AT4G10050	3702.AT4G10050.1	Esterase/lipase/thioesterase family protein; Demethylates proteins that have been reversibly carboxymethylated
3	Blue	3	AT5G16210	3702.AT5G16210.1	HEAT repeat-containing protein
3	Blue	3	TaPTPA42-2-7A	3702.AT4G08960.1	Phosphotyrosyl phosphatase activator (PTPA) family protein

A

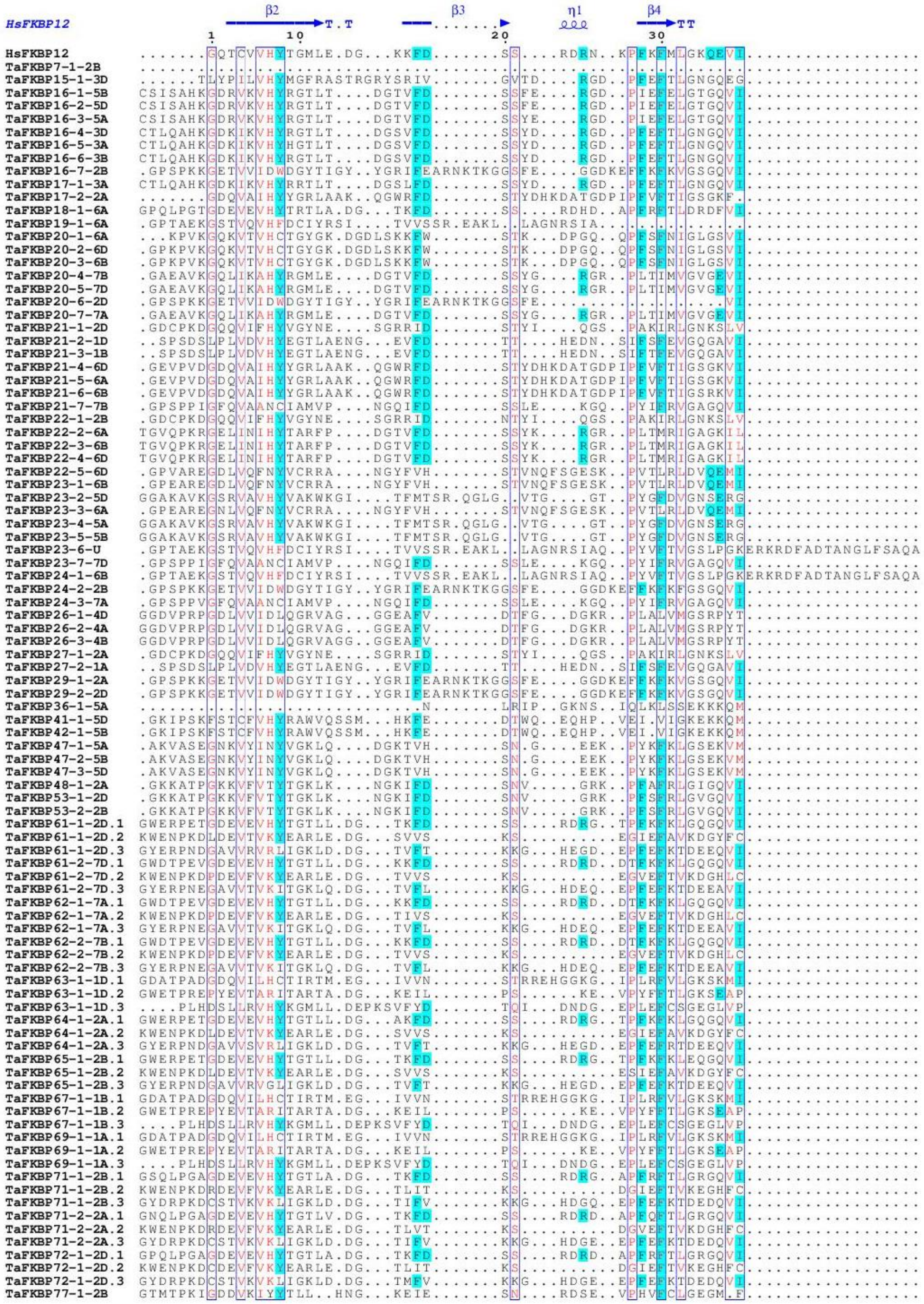
HsFKBP12
β2
β3
η1
β4
1
10
20
30
TaFKBP7-1-2B
TaFKBP15-1-3D
TaFKBP16-1-5B
TaFKBP16-2-5D
TaFKBP16-3-5A
TaFKBP16-4-3D
TaFKBP16-5-3A
TaFKBP16-6-3B
TaFKBP16-7-2B
TaFKBP17-1-3A
TaFKBP17-2-2A
TaFKBP18-1-6A
TaFKBP19-1-6A
TaFKBP20-1-6A
TaFKBP20-2-6D
TaFKBP20-3-6B
TaFKBP20-4-7B
TaFKBP20-5-7D
TaFKBP20-6-2D
TaFKBP20-7-7A
TaFKBP21-1-2D
TaFKBP21-2-1D
TaFKBP21-3-1B
TaFKBP21-4-6D
TaFKBP21-5-6A
TaFKBP21-6-6B
TaFKBP21-7-7B
TaFKBP22-1-2B
TaFKBP22-2-6A
TaFKBP22-3-6B
TaFKBP22-4-6D
TaFKBP22-5-6D
TaFKBP23-1-6B
TaFKBP23-2-5D
TaFKBP23-3-6A
TaFKBP23-4-5A
TaFKBP23-5-5B
TaFKBP23-6-U
TaFKBP23-7-7D
TaFKBP24-1-6B
TaFKBP24-2-2B
TaFKBP24-3-7A
TaFKBP26-1-4D
TaFKBP26-2-4A
TaFKBP26-3-4B
TaFKBP27-1-2A
TaFKBP27-2-1A
TaFKBP29-1-2A
TaFKBP29-2-2D
TaFKBP36-1-5A
TaFKBP41-1-5D
TaFKBP42-1-5B
TaFKBP47-1-5A
TaFKBP47-2-5B
TaFKBP47-3-5D
TaFKBP48-1-2A
TaFKBP53-1-2D
TaFKBP53-2-2B
TaFKBP61-1-2D.1
TaFKBP61-1-2D.2
TaFKBP61-1-2D.3
TaFKBP61-2-7D.1
TaFKBP61-2-7D.2
TaFKBP61-2-7D.3
TaFKBP62-1-7A.1
TaFKBP62-1-7A.2
TaFKBP62-1-7A.3
TaFKBP62-2-7B.1
TaFKBP62-2-7B.2
TaFKBP62-2-7B.3
TaFKBP63-1-1D.1
TaFKBP63-1-1D.2
TaFKBP63-1-1D.3
TaFKBP64-1-2A.1
TaFKBP64-1-2A.2
TaFKBP64-1-2A.3
TaFKBP65-1-2B.1
TaFKBP65-1-2B.2
TaFKBP65-1-2B.3
TaFKBP67-1-1B.1
TaFKBP67-1-1B.2
TaFKBP67-1-1B.3
TaFKBP69-1-1A.1
TaFKBP69-1-1A.2
TaFKBP69-1-1A.3
TaFKBP71-1-2B.1
TaFKBP71-1-2B.2
TaFKBP71-1-2B.3
TaFKBP71-2-2A.1
TaFKBP71-2-2A.2
TaFKBP71-2-2A.3
TaFKBP72-1-2D.1
TaFKBP72-1-2D.2
TaFKBP72-1-2D.3
TaFKBP77-1-2B

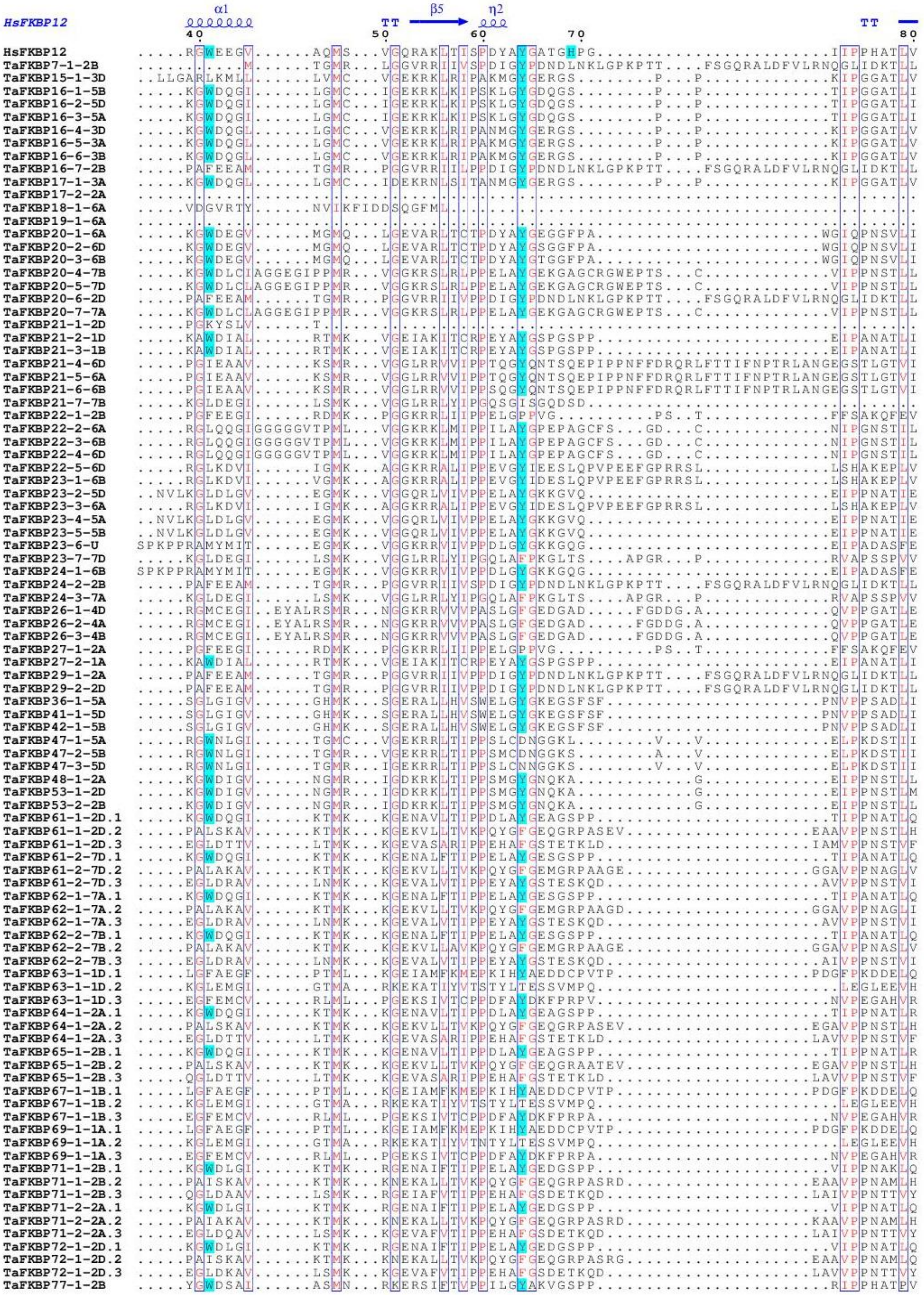

HsFKBP12
α1
β5
η2
TT
TT
40
50
60
70
80
HsFKBP12
TaFKBP7-1-2B
TaFKBP15-1-3D
TaFKBP16-1-5B
TaFKBP16-2-5D
TaFKBP16-3-5A
TaFKBP16-4-3D
TaFKBP16-5-3A
TaFKBP16-6-3B
TaFKBP16-7-2B
TaFKBP17-1-3A
TaFKBP17-2-2A
TaFKBP18-1-6A
TaFKBP19-1-6A
TaFKBP20-1-6A
TaFKBP20-2-6D
TaFKBP20-3-6B
TaFKBP20-4-7B
TaFKBP20-5-7D
TaFKBP20-6-2D
TaFKBP20-7-7A
TaFKBP21-1-2D
TaFKBP21-2-1D
TaFKBP21-3-1B
TaFKBP21-4-6D
TaFKBP21-5-6A
TaFKBP21-6-6B
TaFKBP21-7-7B
TaFKBP22-1-2B
TaFKBP22-2-6A
TaFKBP22-3-6B
TaFKBP22-4-6D
TaFKBP22-5-6D
TaFKBP23-1-6B
TaFKBP23-2-5D
TaFKBP23-3-6A
TaFKBP23-4-5A
TaFKBP23-5-5B
TaFKBP23-6-U
TaFKBP23-7-7D
TaFKBP24-1-6B
TaFKBP24-2-2B
TaFKBP24-3-7A
TaFKBP26-1-4D
TaFKBP26-2-4A
TaFKBP26-3-4B
TaFKBP27-1-2A
TaFKBP27-2-1A
TaFKBP29-1-2A
TaFKBP29-2-2D
TaFKBP36-1-5A
TaFKBP41-1-5D
TaFKBP42-1-5B
TaFKBP47-1-5A
TaFKBP47-2-5B
TaFKBP47-3-5D
TaFKBP48-1-2A
TaFKBP53-1-2D
TaFKBP53-2-2B
TaFKBP61-1-2D.1
TaFKBP61-1-2D.2
TaFKBP61-1-2D.3
TaFKBP61-2-7D.1
TaFKBP61-2-7D.2
TaFKBP61-2-7D.3
TaFKBP62-1-7A.1
TaFKBP62-1-7A.2
TaFKBP62-1-7A.3
TaFKBP62-2-7B.1
TaFKBP62-2-7B.2
TaFKBP62-2-7B.3
TaFKBP63-1-1D.1
TaFKBP63-1-1D.2
TaFKBP63-1-1D.3
TaFKBP64-1-2A.1
TaFKBP64-1-2A.2
TaFKBP64-1-2A.3
TaFKBP65-1-2B.1
TaFKBP65-1-2B.2
TaFKBP65-1-2B.3
TaFKBP67-1-1B.1
TaFKBP67-1-1B.2
TaFKBP67-1-1B.3
TaFKBP69-1-1A.1
TaFKBP69-1-1A.2
TaFKBP69-1-1A.3
TaFKBP71-1-2B.1
TaFKBP71-1-2B.2
TaFKBP71-1-2B.3
TaFKBP71-2-2A.1
TaFKBP71-2-2A.2
TaFKBP71-2-2A.3
TaFKBP72-1-2D.1
TaFKBP72-1-2D.2
TaFKBP72-1-2D.3
TaFKBP77-1-2B

HsFKBP12	β6	
HsFKBP12	FDVELL	KLE
TaFKBP7-1-2B	FDIELI	...
TaFKBP15-1-3D	FDTELI	...
TaFKBP16-1-5B	FDTELV	...
TaFKBP16-2-5D	FDTELV	...
TaFKBP16-3-5A	FDTELV	...
TaFKBP16-4-3D	FDTELI	...
TaFKBP16-5-3A	FDTELI	...
TaFKBP16-6-3B	FDTELI	...
TaFKBP16-7-2B	FDIELI	...
TaFKBP17-1-3A	FDTELI	...
TaFKBP17-2-2A	......	...
TaFKBP18-1-6A	......	...
TaFKBP19-1-6A	......	...
TaFKBP20-1-6A	FEIEVL	...
TaFKBP20-2-6D	FEIEVL	...
TaFKBP20-3-6B	FEIEVL	...
TaFKBP20-4-7B	FDVEYV	...
TaFKBP20-5-7D	FDVEYV	...
TaFKBP20-6-2D	FDIELI	...
TaFKBP20-7-7A	FDVEYV	...
TaFKBP21-1-2D	......	...
TaFKBP21-2-1D	FEVELV	...
TaFKBP21-3-1B	FEVELV	...
TaFKBP21-4-6D	FDIELI	...
TaFKBP21-5-6A	FDIELI	...
TaFKBP21-6-6B	FDIELI	...
TaFKBP21-7-7B	......	...
TaFKBP22-1-2B	FDVELL	...
TaFKBP22-2-6A	YDILLV	...
TaFKBP22-3-6B	YDILLV	...
TaFKBP22-4-6D	YDILLV	...
TaFKBP22-5-6D	FEVQLL	...
TaFKBP23-1-6B	FEVQLL	...
TaFKBP23-2-5D	LDVELL	...
TaFKBP23-3-6A	FEVQLL	...
TaFKBP23-4-5A	LDVELL	...
TaFKBP23-5-5B	LDVELL	...
TaFKBP23-6-U	LNIELL	...
TaFKBP23-7-7D	FDVNLL	...
TaFKBP24-1-6B	LDIELL	...
TaFKBP24-2-2B	FDIELI	...
TaFKBP24-3-7A	FDVNLL	...
TaFKBP26-1-4D	YVVQVD	...
TaFKBP26-2-4A	YVVQVD	...
TaFKBP26-3-4B	YVVQVD	...
TaFKBP27-1-2A	FDVELL	...
TaFKBP27-2-1A	FEVELL	...
TaFKBP29-1-2A	FDIELI	...
TaFKBP29-2-2D	FDIELI	...
TaFKBP36-1-5A	YEVELI	...
TaFKBP41-1-5D	YEVELI	...
TaFKBP42-1-5B	YEVELI	...
TaFKBP47-1-5A	YEVELV	...
TaFKBP47-2-5B	YEVELV	...
TaFKBP47-3-5D	YEVELV	...
TaFKBP48-1-2A	FDVEL.	...
TaFKBP53-1-2D	FDVELM	...
TaFKBP53-2-2B	FDVELM	...
TaFKBP61-1-2D.1	FDVELL	...
TaFKBP61-1-2D.2	IDLEL.	...
TaFKBP61-1-2D.3	YEVDLV	...
TaFKBP61-2-7D.1	FDVELL	...
TaFKBP61-2-7D.2	IDLELV	...
TaFKBP61-2-7D.3	YEVELV	...
TaFKBP62-1-7A.1	FDVELL	...
TaFKBP62-1-7A.2	IDLELV	...
TaFKBP62-1-7A.3	YEVELV	...
TaFKBP62-2-7B.1	FDVELL	...
TaFKBP62-2-7B.2	IDLELV	...
TaFKBP62-2-7B.3	YEVELV	...
TaFKBP63-1-1D.1	FEVEML	...
TaFKBP63-1-1D.2	FEVELV	...
TaFKBP63-1-1D.3	WEIELL	...
TaFKBP64-1-2A.1	FDVELL	...
TaFKBP64-1-2A.2	IDLELV	...
TaFKBP64-1-2A.3	YEVEIV	...
TaFKBP65-1-2B.1	FDVELL	...
TaFKBP65-1-2B.2	IDLQLV	...
TaFKBP65-1-2B.3	YEVELV	...
TaFKBP67-1-1B.1	FEVEML	...
TaFKBP67-1-1B.2	FEVELV	...
TaFKBP67-1-1B.3	WEIELL	...
TaFKBP69-1-1A.1	FEVEML	...
TaFKBP69-1-1A.2	FEVELV	...
TaFKBP69-1-1A.3	WEIELL	...
TaFKBP71-1-2B.1	FDVELL	...
TaFKBP71-1-2B.2	IDLQLV	...
TaFKBP71-1-2B.3	YDVELV	...
TaFKBP71-2-2A.1	FDVELL	...
TaFKBP71-2-2A.2	IDLQLV	...
TaFKBP71-2-2A.3	YDVELV	...
TaFKBP72-1-2D.1	FDVELL	...
TaFKBP72-1-2D.2	IDLQLV	...
TaFKBP72-1-2D.3	YDVELV	...
TaFKBP77-1-2B	......	...

B

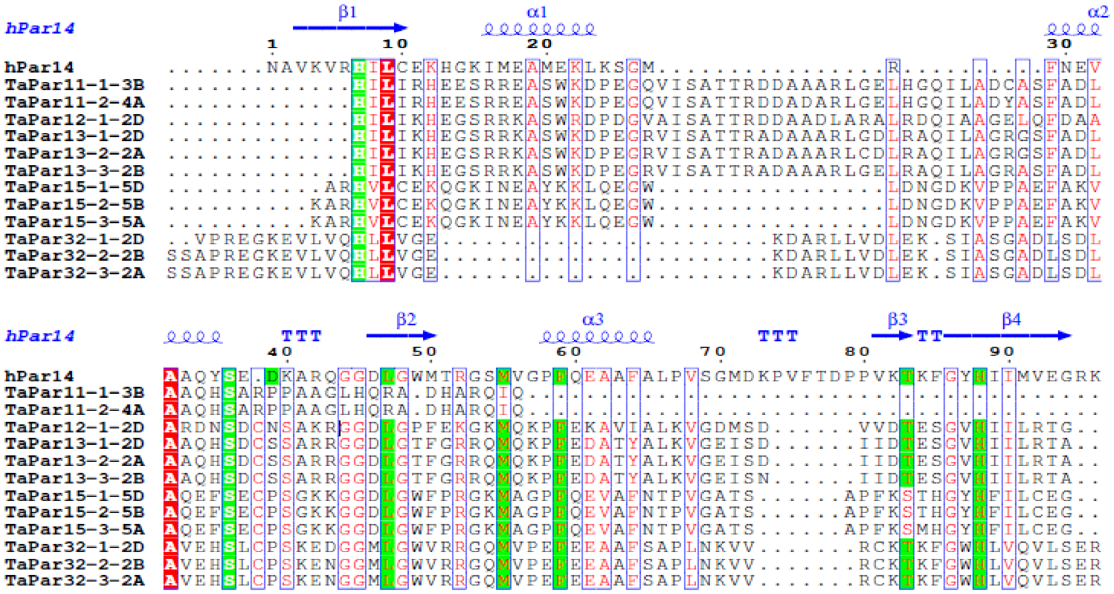

Figure S1. Multiple sequence alignment of amino acid sequences of FKBPs and parvulin proteins of *T. aestivum*. (A) Alignment of FKBPs with reference to the mammalian FKBP12 (Van Duyne et al., 1993). (B) Alignment of parvulins with reference to human parvulin hPar14 (Sekerina et al., 2000). The active site residues of FKBPs and parvulins are shaded with blue and green colors, respectively. The secondary structure elements from the reference structures are shown at the top.

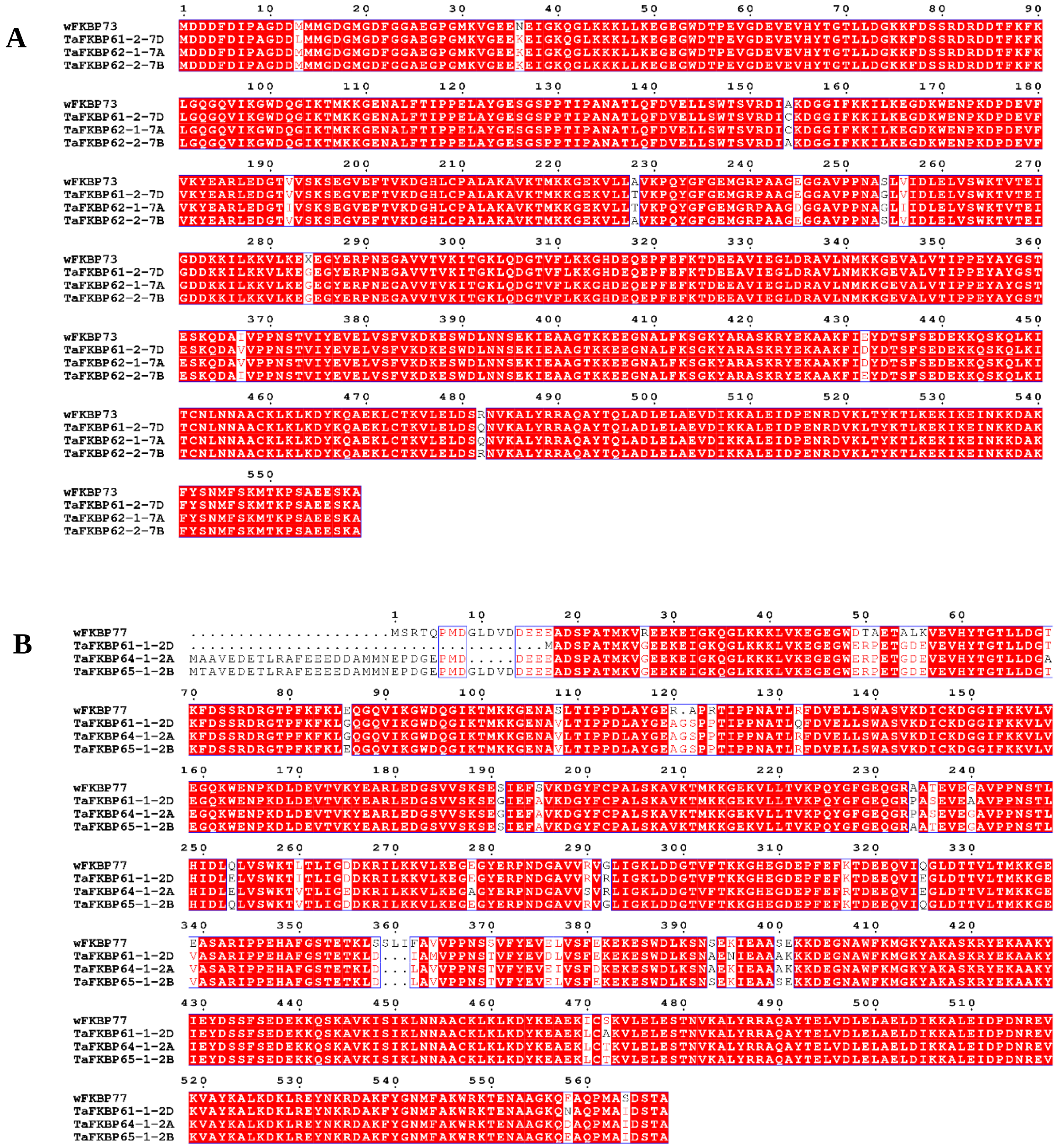

Figure S2. A comparison of multi-FK506-binding domain (FKBD) containing homeolog triplets with their experimentally characterized members (wFKBP73 and wFKBP77). A) Alignment of wFKBP73 (Q43207), TaFKBP-61-2-7D, TaFKBP-62-1-7A and TaFKBP-62-2-7B. B) Alignment of wFKBP77 (CAA68913.1), TaFKBP-61-1-2D, TaFKBP-64-1-2A andTaFKBP-65-1-2B.
